# CD4^+^ mucosal-associated invariant T cells express highly diverse T cell receptors

**DOI:** 10.1093/jimmun/vkaf260

**Published:** 2025-11-09

**Authors:** Rimanpreet Kaur, Danielle Xie, Atul Pradhan, Nezar Mehanna, Kelin Li, Jeffrey Aubé, Barbara Rosati, David Carlson, Christina Y Lee, Charles Kyriakos Vorkas

**Affiliations:** Division of Infectious Diseases, Department of Medicine, Renaissance School of Medicine at Stony Brook University, Stony Brook, NY, USA; Department of Microbiology and Immunology, Renaissance School of Medicine at Stony Brook University, Stony Brook, NY, USA; Center for Infectious Diseases, Renaissance School of Medicine at Stony Brook University, Stony Brook, NY, USA; Division of Infectious Diseases, Department of Medicine, Renaissance School of Medicine at Stony Brook University, Stony Brook, NY, USA; Department of Microbiology and Immunology, Renaissance School of Medicine at Stony Brook University, Stony Brook, NY, USA; Center for Infectious Diseases, Renaissance School of Medicine at Stony Brook University, Stony Brook, NY, USA; Division of Infectious Diseases, Department of Medicine, Renaissance School of Medicine at Stony Brook University, Stony Brook, NY, USA; Department of Microbiology and Immunology, Renaissance School of Medicine at Stony Brook University, Stony Brook, NY, USA; Center for Infectious Diseases, Renaissance School of Medicine at Stony Brook University, Stony Brook, NY, USA; Division of Infectious Diseases, Department of Medicine, Renaissance School of Medicine at Stony Brook University, Stony Brook, NY, USA; Department of Microbiology and Immunology, Renaissance School of Medicine at Stony Brook University, Stony Brook, NY, USA; Center for Infectious Diseases, Renaissance School of Medicine at Stony Brook University, Stony Brook, NY, USA; Division of Chemical Biology and Medicinal Chemistry, UNC Eshelman School of Pharmacy, University of North Carolina at Chapel Hill, Chapel Hill, NC, USA; Division of Chemical Biology and Medicinal Chemistry, UNC Eshelman School of Pharmacy, University of North Carolina at Chapel Hill, Chapel Hill, NC, USA; Department of Physiology and Biophysics, Renaissance School of Medicine at Stony Brook University, Stony Brook, NY, USA; Single Cell and Spatial Transcriptomics Facility, Renaissance School of Medicine at Stony Brook University and Northport Veterans Affairs Medical Center, Stony Brook and Northport, NY, USA; Institute for Advanced Computational Science, Stony Brook University, Stony Brook, NY, USA; Division of Hematology and Medical Oncology, Department of Medicine, Renaissance School of Medicine at Stony Brook University, Stony Brook, NY, USA; Division of Infectious Diseases, Department of Medicine, Renaissance School of Medicine at Stony Brook University, Stony Brook, NY, USA; Department of Microbiology and Immunology, Renaissance School of Medicine at Stony Brook University, Stony Brook, NY, USA; Center for Infectious Diseases, Renaissance School of Medicine at Stony Brook University, Stony Brook, NY, USA

**Keywords:** CD4^+^ MAIT cells, TCR diversity, CITE-Seq, single cell genomics, innate lymphocytes

## Abstract

Mucosal-associated invariant T (MAIT) cells are highly conserved innate-like T cells in mammals recognized for their high baseline frequency in human blood and cytotoxic effector functions during infectious diseases, autoimmunity, and cancer. While the majority of these cells in humans express a conserved CD8αβ^+^ TRAV1-2 T cell receptor (TCR) recognizing microbially derived vitamin B2 intermediates presented by the evolutionarily conserved major histocompatibility complex class I–related molecule, MR1, there is an emerging appreciation for diverse MAIT cell subsets that possess distinct functions including CD4^+^ MAIT cells that remain underexplored. In this study, we adopted an unbiased single-cell TCR-sequencing approach in MR1-5-OP-RU-tetramer–reactive T cells. We discovered that CD4^+^ MAIT cells are enriched with highly diverse TRAV1-2^−^ TCRs. To specifically characterize this TCR repertoire, we analyzed VDJ sequences across 2 datasets and identified distinct TCR usage among CD4^+^ MAIT cells including TRAV21, TRAV8 (TRAV8-1, TRAV8-2, TRAV8-3), and TRAV12 families (TRAV12-2, TRAV12-3), as well as more variable J segment, CDR3α, and TRBV sequences. TRAV1-2^−^ MAIT cell TCRs were also enriched after in vitro culture with interleukin-2 and *Mycobacterium tuberculosis*. These results indicate that mature human CD4^+^ MAIT cells adopt distinct TCR usage from the canonical TRAV1-2^+^ CD8^+^ subset and suggest that alternative MR1 ligands in addition to riboflavin intermediates may select for them.

## Teaser

The Editors have selected this article as a highlight of the issue.

## Introduction

Mucosal-associated invariant T (MAIT) cells are innate-like lymphocytes and are among the first responders during infectious or noninfectious inflammatory diseases, including tuberculosis.^1–4^ MAIT cells recognize microbial antigens of the vitamin B2 (riboflavin) pathway bound to the major histocompatibility complex class I–related protein 1 (MR1) receptors on antigen-presenting cells.^5–9^ They are phenotypically and functionally distinct from conventional peptide-specific T lymphocytes, including high surface expression of C-type lectin receptor, CD161, and conserved T cell receptors (TCRs) (most are TRAV1-2^+^ in *Homo sapiens*).^10^ The advent of MR1 tetramers^11–15^ has enabled the identification of antigen-specific MAIT cells and the most commonly used are MR1-5-OP-RU tetramers, synthesized using the potent activating ligand 5-OP-RU derived from a riboflavin biosynthetic intermediate, that can specifically identify MAIT cells by flow cytometry.^1^^,^^3^^,^^11^^,^^12^^,^^14^^,^^16^ The biochemical distinction conferring riboflavin intermediate ligand capacity to bind and activate MAIT cells is a ribityl tail, which is necessary for MAIT cell TCR engagement^17^ and is highly biased toward canonical MAIT cell TCRs comprising a TRAV1-2 α chain paired with TRAJ33/12/20 and TRBV6/20.^7^^,^^18^ However, variation in the TRAV and TRBV usage has also been reported to confer pathogen specificity.^7^^,^^19^

In contrast to mature murine MAIT cells that are largely CD4^−^CD8^−^ (double negative [DN])^2^ or nonhuman primate MAIT cells that are primarily CD8^+^,^20^^,^^21^ mature human MAIT cells coexpress CD8 or CD4 molecules like conventional T cells.^1^^,^^3^ While CD8 stabilizes MR1 binding to enhance antigen responsiveness analogous to major histocompatibility complex class I,^22^ the role of CD4 coexpression on MAIT cells is less understood. Murine studies demonstrate loss of CD4 expression on MAIT cells during thymic development and conclude that CD4 may be a marker of immaturity in mice.^23–25^ The increased presence of mature CD4^+^ MAIT cells in human blood and tissues generates an alternative hypothesis that they may be selected after thymic egress.^3^^,^^21^ We and others previously showed that human circulating CD4^+^ MAIT cells adopt distinct phenotypic states from their CD8^+^ counterparts in healthy states including during asymptomatic human *Mycobacterium tuberculosis* (*Mtb*) exposure and infection.^1^^,^^26–28^ Distinctions include differential expression of IL2R, FOXP3, CCR7, CD62L, TNFRSF4, and TNFRSF13 proteins, indicating selection by alternative costimulatory signals and potential to migrate into lymphatic tissue. Interestingly, CD4^+^ MAIT cells demonstrate striking similarity both transcriptionally and immunophenotypically to regulatory T cells.^1^ As CD4^+^ MAIT cells demonstrate a weaker response to in vitro stimulation with canonical riboflavin-derived activating ligands relative to CD8^+^ MAIT cells, it is also hypothesized that they may respond to distinct classes of MR1 ligands from the riboflavin pathway that remain undiscovered.^1^^,^^29^ Prior studies of human MAIT cell TCR diversity have focused on CD8 and TRAV1-2 coexpression and characterization of TRBV gene usage.^30^^,^^31^ In the present study, we tested the hypothesis that CD4 or CD8 coexpression in MAIT cells correlates with distinct TCR usage by employing unbiased single cell TCR sequencing analysis of unfractionated MR1-5-OP-RU tetramer^+^ MAIT cell subpopulations. We discovered that CD4^+^ MAIT cells express unexpectedly diverse VDJ genes and resembled private TCRs, including significant variation in TCRα, J segment, CDR3α, and TCRβ sequences compared with CD8^+^ MAIT cells. Unique TRAV1-2^−^ CD4^+^ MAIT cells TCRs were also detected after in vitro incubation with interleukin-2 (IL-2) and *Mtb* lysates. Together, our results suggest that human CD4^+^ MAIT cell TCRs may be selected by alternative MR1 ligands in addition to canonical riboflavin intermediates.

## Methods

### Human subject recruitment and peripheral blood mononuclear cell isolation

Healthy donors were recruited at Stony Brook University (SBU) under IRB2021-00478 (principal investigator: C.K.V.) or purchased from the New York Blood Center for use in single-cell transcriptomic and flow cytometric experiments. Peripheral blood mononuclear cells (PBMCs) were isolated using Ficoll (Fisher Scientific) or SepMate tubes (STEMCELL Technologies) using the manufacturer’s protocol. The isolated PBMCs were stored in Bambanker serum-free media (GC-Lymphotec) in liquid nitrogen and thawed as needed for immunology assays.

### Flow cytometric PBMC stimulation assay

Cryopreserved healthy donor PBMCs (n = 13) were thawed and 200,000 cells per well were cultured in U-bottom plates at 37 °C, 5% CO_2_ for either 16 h or 7 d ± stimulation conditions detailed subsequently. Cells were maintained in complete RPMI 1640 media (Quality Biologicals) supplemented with 10% (v/v) heat-inactivated fetal bovine serum, penicillin/streptomycin (100 U/mL), L-glutamine (2 mM), sodium pyruvate (1 mM), nonessential amino acids (0.1 mM), HEPES buffer (10 mM), and 2-mercaptoethanol (50 µM), and supplemented with recombinant human IL-2 (50 U/mL; PeproTech). Stimulation conditions included MR1 ligands 5-OP-RU (2 mM) and folate catabolites Ac-6-FP (2 µM) and 6-FP (2 µM) in addition to nonspecific T cell stimulation with anti-CD3/CD28 magnetic beads (Dynabeads; Invitrogen) at a 1:100 bead-to-cell ratio (Dynabeads), PMA 10 ng/mL, IL-12 (1 µg/mL), IL-15 (100 ng/mL), IL-18 (100 ng/mL), and *Mtb* Erdman whole cell lysate (1:100). *Mtb* whole cell lysate was prepared from freshly cultured *Mtb* Erdman strain grown to optical density (OD600) of 0.8 in 7H9 liquid media in 50 mL prior to bead-mediated lysis in a Biosafety level 3 facility at SBU.

### Flow cytometry staining and analysis

**Figure 1.**
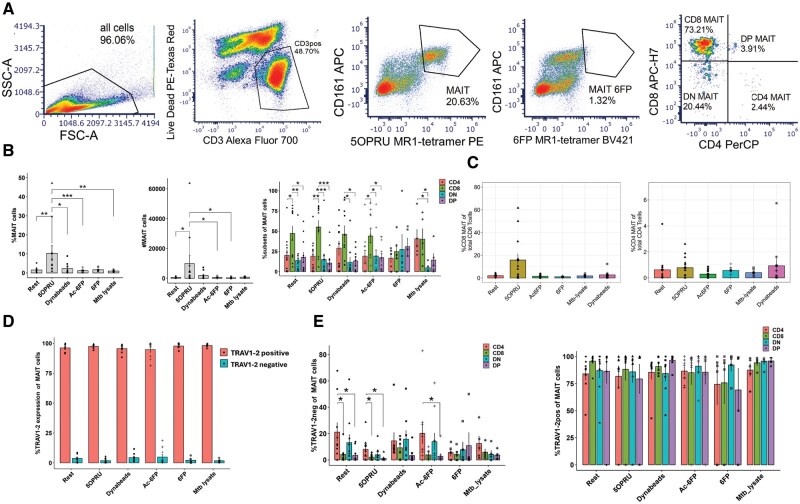
CD4^+^ MAIT cells are more likely to express TRAV1-2^−^ TCRs. (A) Representative flow cytometric gating strategy of MAIT cells and CD4^+^/CD8^+^ subsets after 7 d of in vitro incubation with 5-OP-RU. (B) Total MAIT cells and subset frequency after 7 d of incubation in vitro with various stimuli. Absolute counts (#MAIT cells) were normalized per 10,000 live cells. (C) Frequency of CD8^+^ and CD4^+^ MAIT cells of total CD8^+^ and CD4^+^ T cells after 7 d of in vitro stimulation. (D) MAIT cell frequency stratified by TRAV1-2 expression. (E) Frequency of TRAV1-2^−^ (left) and TRAV1-2^+^ (right) MAIT cells stratified by subset. Statistical comparisons made by unpaired *t* test. *P < 0.05, **P < 0.005, ***P < 0.001. DN, double negative; DP, double positive; FSC-A, forward scatter area; SSC-A, side scatter area.

PBMCs were F_c_R-blocked (1:20 dilution; BD Pharmingen) for 15 min at room temperature (RT), stained for cell surface fluorescent antibody cocktail for 15 min at RT, followed with IC fixation (Invitrogen) (see Table S1 for a complete list of reagents). Cells were then acquired on an Aurora spectral analyzer (Cytek), and flow cytometry data were analyzed using FCS Express 7(De Novo Software) with manual gating. Graphs were generated on RStudio using packages ggplot2, rstatix, and dplyr. MAIT cells were identified as Live CD3^+^MR1-5-OP-RU tetramer^+^CD161^++^ MAIT cells in all experiments (gating strategy in Fig. 1A). Absolute counts (#MAIT cells) were normalized per 10,000 live cells.

### Single-cell sorting and CITE-Seq staining

Cryopreserved PBMCs from 2 healthy donors were thawed and either sorted directly ex vivo or cultured for 7 d in IL-2± *Mtb* whole cell lysate for CITE-Seq (Cellular Indexing of Transcriptomes and Epitopes by sequencing). Cells were cultured in flat, nontreated sterile 24-well plates (1M cells/well) using complete RPMI 1640 media supplemented with IL-2 (50 U/mL; PeproTech) at 37 °C and 5% CO_2_.

PBMCs were prepared for CITE-Seq by F_c_R-block (eBioscience) incubation for 15 min at RT, then stained with fluorescent antibody cocktail for 15 min at RT. The following populations were then sorted using a BD FACSAria in the SBU Flow Cytometry Core Facility: MAIT cells (CD3^+^, CD161^++^, 5-OP-RU MR1 tetramer^+^), natural killer cells (CD3^−^, CD14^−^, CD19^−^, CD16^+/-^CD56^+/−^), γδ T cells (CD3^+^, TCR γδ^+^), and invariant natural killer T cells (CD3^+^, CD161^+^, Vα24Jα18^+^) (see Table S1 for a complete list of reagents). Sorted cells were collected in complete RPMI media and washed with cold phosphate-buffered saline, blocked with Human Truestain F_c_X (BioLegend) for 10 min at 4 °C, and then incubated with hashtag and TotalSeq-C antibody cocktail for 30 min at 4 °C (Table S1). Following incubation, cells were centrifuged at 400 rcf for 5 min to remove TotalSeq-C antibodies and resuspended in sterile cold phosphate-buffered saline + 0.04% bovine serum albumin for downstream 5′ single-cell immune profiling assay.

### Single-cell immune profiling assay and analysis

The experimental single cell immune profiling procedure and analysis used in this study is similar to our previous work.^32^ Cell viability and count of each sample was measured by Countess 3FL with trypan blue staining. Approximately 30,000 cells (5,000 cells per sample) were loaded on a 10x Genomics Next GEM Single Cell 5′ Gel Bead Kit in the SBU Single-Cell Genomics Core Facility. Gene expression, surface antigen, and VDJ (TCR) libraries were prepared using Chromium Next GEM 5′ Single Cell with Feature Barcode and VDJ assay, according to the manufacturer’s instructions. The libraries were sequenced at a depth of approximately 25,000 (Gene expression), 10,000 (surface antigen), and 10,000 (VDJ) paired reads per cell on an Illumina NovaSeqX sequencer (Novogene). The gene expression, surface feature barcode raw FASTQ files were analyzed on Cell Ranger (v.7.0; 10x Genomics) using multipipeline with human reference transcriptome (GRCh38) downloaded from the 10x Genomics website and hashtag configuration file, which contained the barcodes related to each sample. The obtained BAM files were converted to FASTQ files and again run on Cell Ranger for surface antigen and VDJ analysis.

The VDJ output was merged with the gene expression matrix using djvdj package on R (v.4.3.2; R Foundation for Statistical Computing) and then preprocessed using the *Seurat* (v.4.0) package. Quality control steps included removal of cells with more than 6,000 gene counts and more than 5% mitochondrial content. The top 3,000 variable features were identified using *FindVariableFunction*() and normalized independently for each sample prior to integration with the *Harmony* R package. After integration of all samples, principal component analysis was performed, and the first 20 principal components were selected to perform clustering with *FindCluster*() at a resolution of 0.8. Dimensionality reduction and visualization was performed with Uniform Manifold Approximation and Projection map using default Louvain algorithm. We first analyzed cells with a single paired TCR α and β chain transcript and then stratified by TRAV1-2 transcript using VDJ sequences to identify alternative TCR usage. Single cells with dual TCR α chains were also analyzed.

### Validation of MAIT single-cell transcriptome in a separate dataset

To augment the sample size of single MAIT cells and validate our results, we also used a publicly available single-cell transcriptome and VDJ raw dataset generated by CITE-Seq of MAIT cells that were similarly defined as MR1-5-OP-RU tetramer^+^ in Garner et al annotated experiment #1.^30^ The data were retrieved and analyzed using an earlier version of the pipeline in RStudio with Seurat package. Cells with >8% mitochondrial gene content and gene count above 3,000 were removed during quality control. Scaling and log normalization were performed to preprocess data for principal component analysis, and the first 20 principal components were chosen for clustering and visualization of cells in Uniform Manifold Approximation and Projection (UMAP) or t-distributed stochastic neighbor embedding (t-SNE). VDJ data were combined with single-cell transcriptome data using the djvdj package, and paired TCRs comprising single α and β chains were selected for further analysis.

### Gene Ontology resource annotations and TCR functional analysis

The epitope prediction for *TRAV1-2^+/-^* MAIT cells was performed using McPAS-TCR (Pathology-associated TCR database) (http://friedmanlab.weizmann.ac.il/McPAS-TCR/). This database comprises TCR sequences associated with different pathological conditions and can be queried by TCR composition, CDR3 sequence, epitope, tissue, major histocompatibility complex restriction, and other criteria.^33^ The CDR3 sequences detected in our study and present in the McPAS-TCR dataset were then used to predict putative epitope specificities, including analyses following in silico amino acid insertions (+1, +2) or deletions (−1, −2) within the CDR3α or CDR3β region. Further, we performed Kyoto Encyclopedia of Genes and Genomes (KEGG) analysis for functional predictions and Gene Ontology annotations to predict *TRAV1-2*^+/-^ MAIT cell biological functions using the KEGG library (KEGGREST package) in R.

## Results

### CD4^+^ MAIT cells are enriched with TRAV1-2^−^ TCRs

To identify the proportion of MAIT cells with noncanonical TCR expression and define their reactivity to MR1 ligands, we stained healthy donor PBMCs and analyzed MR1-5-OP-RU tetramer^+^CD161^++^ cell frequency using spectral flow cytometry (n = 13). MAIT cells (defined as Live CD3^+^MR1-5-OP-RU tetramer^+^ CD161^++^) were then stratified by CD4 or CD8 surface expression into 4 subsets: CD8^+^, CD4^+^, DN (CD8^−^CD4^−^), and double positive (CD8^+^CD4^+^) (Fig. 1A).

We assessed the frequency of each subset at baseline and after 7 d in vitro expansion using MR1 ligands, anti-CD3/CD28 Dynabeads or *Mtb* lysates. We observed that total MAIT cells significantly expanded with the activating MR1 ligand, 5-OP-RU, but not with folate derivatives (6-FP and Ac-6-FP), nonspecific T cell stimulation with anti-CD3/CD28, or *Mtb* whole cell lysates (Fig. 1B). We confirmed our previous findings that predominantly CD8^+^ MAIT cells expanded with 5-OP-RU antigen relative to CD4^+^ MAIT cells^1^ and did not observe any significant effect of folate catabolites Ac-6-FP or 6-FP on the relative distribution of MAIT cell subsets (Fig. 1B, C). We found that CD8^+^ MAIT cells composed 5 ± 3% (SEM) of CD8^+^ T cells at rest in IL2-alone and expanded to 20 ± 10% (SEM) after 5-OP-RU induction with variability across donors. In contrast, CD4^+^ MAIT cells composed <1% of CD4^+^ T cells at baseline with nominal expansion after 5-OP-RU or anti-CD3/CD28 among CD4^+^ T cells in some donors (Fig. 1C).

Next, we stratified our analyses by TRAV1-2 surface staining and found that most 5-OP-RU–induced MAIT cells were TRAV1-2^+^ (Fig. 1D; Fig. S1A). The frequency of TRAV1-2^−^ TCRs was significantly higher among CD4^+^ MAIT cells than CD8^+^ MAIT cells in resting and 5-OP-RU conditions (Fig. 1E). TRAV1-2^−^ MAIT cells were detectable across subsets, and their expansion was observed in the CD8^+^ subset after 5-OP-RU induction in some donors (Fig. S1B and C).

### CITE-Seq defines functionally distinct CD4^+^ and CD8^+^ MAIT cell populations

**Figure 2.**
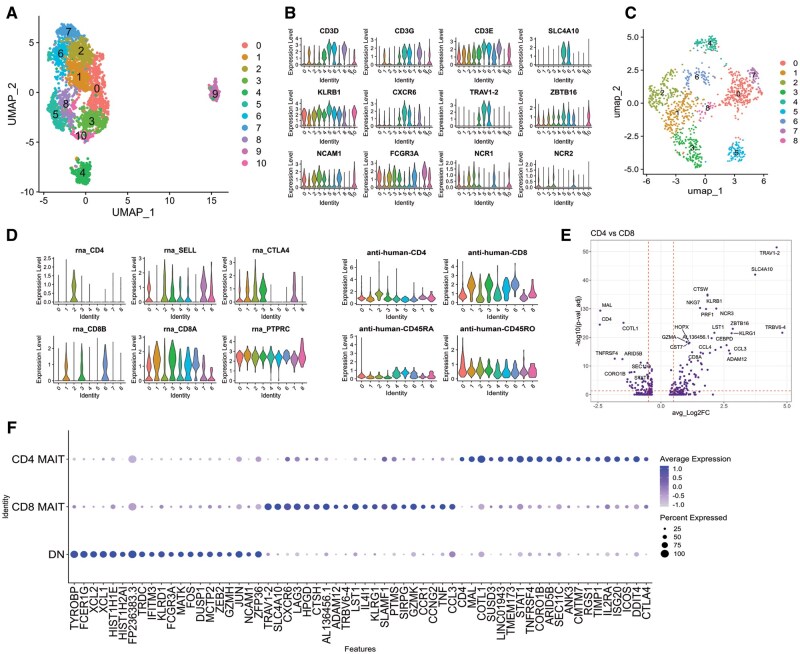
MAIT cell identification and visualization by CITE-Seq reveals distinct CD4^+^ and CD8^+^ MAIT subsets. (A) Uniform Manifold Approximation and Projection (UMAP) visualization of all sequenced cells. (B) Violin plots demonstrating differential gene expression between clusters. (C) UMAP visualization of MAIT cell subclusters. (D) Violin plots displaying the expression of CD4 and CD8 gene transcripts and surface proteins detected by anti-human-CD4, anti-human-CD8, anti-human-CD45RO, and anti-human-CD45RA barcodes in MAIT cell subclusters. (E) Differentially expressed genes in CD8^+^ (+log fold change) and CD4^+^ MAIT cells (−log fold change). The y-axis uses log false discovery rate–adjusted *P* value with a significance level of false discovery rate–adjusted *P* < 0.05. (F) Dot plot displaying differential gene analysis between MAIT cell subsets; color intensity represents the average gene expression per cell and the dot size represents the frequency of cells expressing each gene.

To define the transcriptional signature and alternative TCR usage of these CD4^+^ and CD8^+^ MAIT cell subpopulations during homeostasis and activation, we performed CITE-Seq with single-cell VDJ sequencing of sorted MAIT cells (CD3^+^MR1-5-OP-RU tetramer^+^CD161^++^ from 2 healthy donors directly ex vivo after thaw and after in vitro incubation for 7 d with IL-2± *Mtb* whole cell lysate to select for *Mtb*-reactive MAIT cells. First, we defined the transcriptional and surface barcode antibody profile of all sequenced cells in 11 clusters (Fig. 2A; Fig. S2A) and performed differential gene expression analysis between clusters (Fig. S2B). The presence of *CD3D*, *CD3G*, and *CD3E* gene transcripts, CD3 surface protein expression, and differential expression of canonical genes *KLRB1*, *ZBTB16*, *IL18R1*, *CXCR6*, *SLC4A10*, and *TRAV1-2* distinguished MAIT cells from other innate lymphocytes sorted in the same batch (Fig. 2B; Fig. S2C).^34^

**Figure 3.**
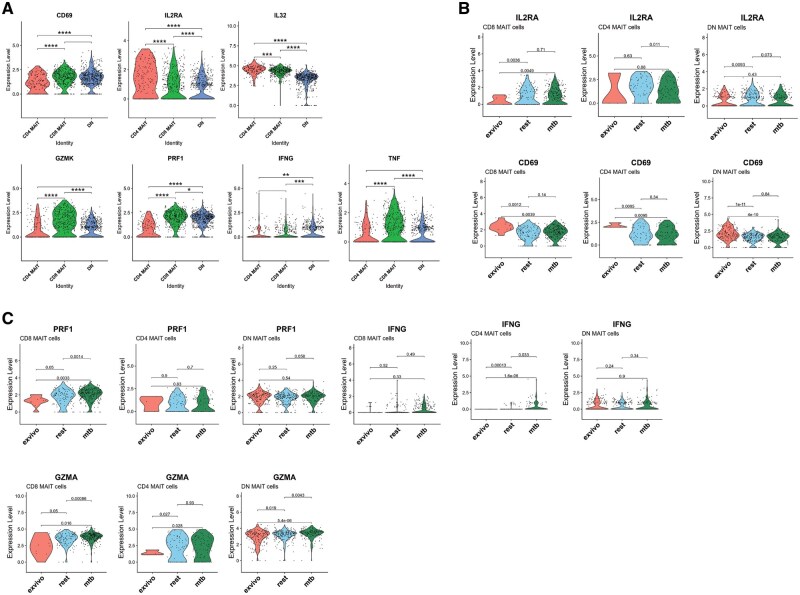
CD4^+^ and CD8^+^ MAIT cell gene expression analysis. (A) Violin plots displaying gene expression levels of activation and effector transcripts in MAIT cell subsets in pooled experimental conditions. (B, C) The expression of activation (*CD69* and *IL2RA*) (B) and effector (*PRF1*, *GZMA*, *IFNG*) (C) genes stratified by in vitro stimulation conditions and MAIT cell subsets. Statistical comparisons made by unpaired Wilcoxon test, *P* < 0.05 significant, *P* value adjusted. *P < 0.05, **P < 0.005, ***P < 0.001, ****P < 0.0001.

In our CITE-Seq dataset, we found 9 MAIT cell subclusters referred to from here on as C0 to C8 and annotated CD8^+^, CD4^+^, and DN MAIT cells (Fig. 2C, D). We stratified CD4^+^ and CD8^+^ MAIT cells based on the presence of CD4/CD8 transcript and surface protein expression detected by antibody barcode. We observed that 50% of the sequenced MAIT cells were DN, 35% CD8^+^, and 15% CD4 (Fig. S2D, E). We unexpectedly found that all MAIT cells had detectable *CD8A* transcript regardless of CD4 or CD8 surface protein expression, whereas *CD8B* transcript expression correlated with CD8 protein expression to define CD8^+^ MAIT cells and thus considered C1, C3, and C6 as CD8^+^ MAIT cells based on *CD8B* transcript and anti-CD8 barcode (Fig. 2D). C2 expressed CD4 surface protein and *CD4* transcript and was considered a CD4^+^ MAIT cell cluster (Fig. 2D). The CITE-Seq technology also enables accurate annotation of DN MAIT cells that were CD4/CD8 surface barcode and *CD8B*/*CD4* transcript negative, in contrast to prior work that relied on surface protein or transcript alone and was potentially subject to “dropout.”^35^^,^^36^ C0, C4, C5, C7, and C8 were considered DN MAIT cells. Double positive MAIT cells (CD4^+^CD8^+^ surface barcodes) were not detected in our dataset (Fig. 2D). We confirm and extend previous findings^1^ that CD4^+^ and CD8^+^ MAIT cells have distinct transcriptional signatures; CD4^+^ MAIT cells differentially expressed costimulatory receptors *CTLA4*, *TNFRSF4*, the cytokine IL-32 and the IL-2 receptor α (*IL2RA*), while CD8^+^ MAIT cells differentially expressed a cytotoxic program including *NKG7*, *KLRG1*, *EOMES*, *GNLY*, PRF, *GZMK*, and *TNF* (Figs. 2E and 3A; Fig. S3 and Table S2). DN MAIT cells differentially expressed activation-associated genes such as *JUN*, *FOS*, *DUSP1*, *XCL1*, *XCL2*, and *IFITM3* (Fig. 2F). We next stratified our analyses by *Mtb* stimulation condition and found that CD8^+^ MAIT cells significantly upregulated transcription of *GZMA*, *GZMK*, *PRF1*, and IL32; CD4^+^ MAIT cells upregulated *IL2RA* and *IFNG;* and DN MAIT cells upregulated *GZMA* relative to IL-2-alone resting conditions (Fig. 3B, C; Fig. S3).

**Figure 4.**
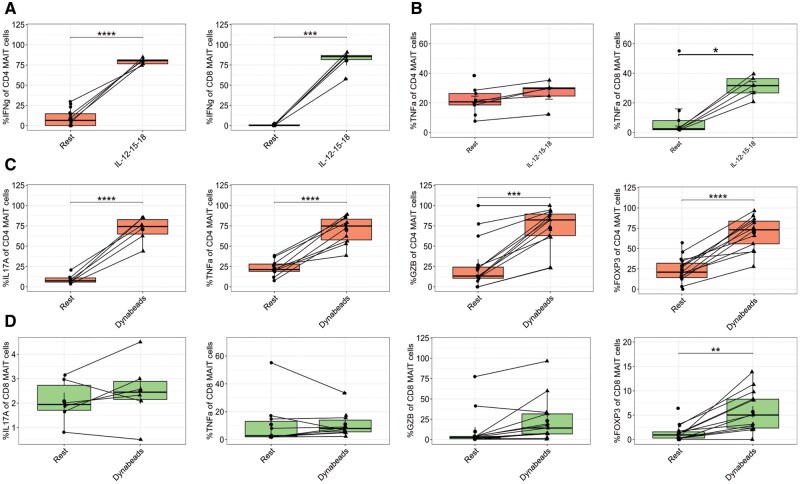
CD8^+^ and CD4^+^ MAIT cell effector function after stimulation with cytokines or anti-CD3/CD28 Dynabeads. Bar plots display the percent expression of (A) IFNγ and (B) TNFα in CD4^+^ and CD8^+^ MAIT cells after 16 h of incubation with IL-12/15/18. (C) IL-17A, TNFα, granzyme B (GZB), and FOXP3 expression after incubation with anti-CD3/28 Dynabeads in CD4^+^ MAIT cells and (D) in CD8^+^ MAIT cells. Orange color represents CD4^+^ MAIT cells and green color represents CD8^+^ MAIT cells. Statistical comparisons made by paired *t* test. *P < 0.05, **P < 0.005, ***P < 0.001, ****P < 0.0001.

We next interrogated differences in function between CD8^+^ and CD4^+^ MR1-5-OP-RU tetramer^+^ CD161^++^ cells after incubation in vitro with TCR-dependent and TCR-independent activation conditions followed by intracellular antibody staining for flow cytometry (Fig. 4; Figs. S4 and S5). We observed CD8^+^ MAIT cells displayed significant granzyme B upregulation in response to purified 5-OP-RU antigen, not observed in CD4^+^ MAIT cells (Fig. S4D).^1^ In response to combined cytokine stimulation with IL-12/15/18, CD8^+^ MAIT cells upregulated GZMB, IFNγ, and TNFα while CD4^+^ MAIT cells only upregulated IFNγ (Fig. 4A, B; Fig. S4D). Unexpectedly, anti-CD3/CD28 induction with Dynabeads significantly upregulated IL-17, TNFα, granzyme B, and FOXP3 in CD4^+^ MAIT cells, whereas CD8^+^ MAIT cells upregulated FOXP3 and to a lesser extent IFNγ (Fig. 4C, D; Figs. S4C, D and S5). Together, these results support distinct effector functions of CD4^+^ and CD8^+^ MAIT cells following TCR-dependent and independent stimuli and highlight that the CD4^+^ subset is less responsive to 5-OP-RU and is poised to respond to cytokines and TCR engagement with alternative ligands. However, we could not find consistent expansion of TRAV1-2^−^ MAIT cells with TCR independent (IL-12/15/18) or nonspecific T cell stimulation (Dynabeads) after 7 d of in vitro stimulation. The frequency of TRAV1-2^−^ CD4^+^ MAIT cells demonstrated donor variability (Fig. S5C).

### CD4^+^ MAIT cells express diverse TCRs

**Figure 5.**
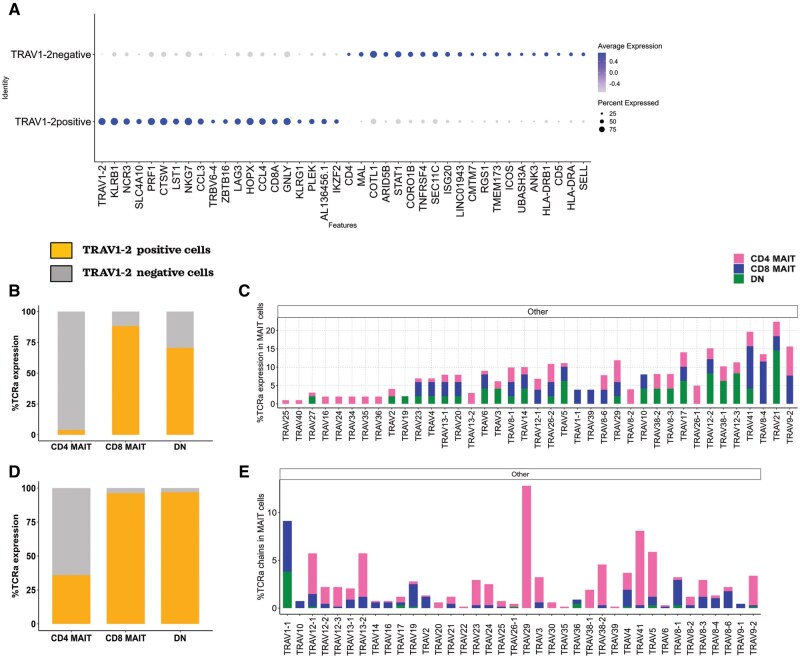
CD4^+^ MAIT cells predominantly express *TRAV1-2*^−^ TCRs in Kaur et al and Garner et al^28^ datasets. (A) Dot plot displaying the top differentially expressed genes between MR1-5-OP-RU tetramer^+^  *TRAV1-2*^+^and *TRAV1-2*^-^ MAIT cells in Kaur et al. (B) Bar plot showing the proportion of cells expressing the canonical *TRAV1-2* TCR (yellow) or *TRAV1-2*^−^ TCRs (gray) in each MAIT cell subset. (C) TCRα diversity measured in pooled experimental conditions stratified by MAIT cell subset in Kaur et al. (D) Bar plot showing the proportion of cells expressing the canonical *TRAV1-2* TCR (yellow) or *TRAV1-2*^−^ TCRs (gray) in each MAIT cell subset in Garner et al. (E) TCRα diversity measured in pooled experimental conditions stratified by MAIT cell subset in Garner et al. Pink, blue, and green colors represent CD4^+^, CD8^+^, and DN MAIT cells, respectively.

We next tested the hypothesis that these functionally distinct CD4^+^ and CD8^+^ MAIT cells adopt different TCR usage by integrating VDJ sequencing analyses of sorted MAIT cells from our laboratory (2 donors) with one published and publicly available dataset from Garner et al^30^ that also employed VDJ sequencing of MR1-5-OP-RU-tetramer^+^ cells (3 donors) but restricted most of the published analysis to *CD8^+^TRAV1-2*^+^ MAIT cells (Fig. S6 and S7). MAIT cells were identified in both datasets using a combination of transcripts including *KLRB1*, *IL18R1*, and *ZBTB16* (Figs. S2 and S6) and then stratified by coexpression of *CD4* and *CD8B* (Figs. S2 and S7). After integration, we observed that *TRAV1-2*^+^ MAIT cells differentially expressed cytotoxic gene signatures similar to CD8^+^ MAIT cells including *KLRB1*, *SLC4A10*, *ZBTB16*, *NKG7*, and *CCL3*, whereas *TRAV1-2^−^* MAIT cells differentially expressed *CD4* in addition to noncytotoxic and costimulatory genes, including *ICOS*, *SELL*, *MAL*, *TNFRSF4*, and *HLA-DRA* (Fig. 5A).

We identified more than 7,587 paired TCRs in MAIT cells from 5 donors. First, we performed VDJ analysis of all MAIT cells irrespective of experimental condition and observed that 90% of CD4^+^ MAIT cells had noncanonical *TRAV1-2*^−^ TCRs relative to 70% from Garner et al.^30^ In contrast, only 15% of CD8^+^ and 35% of DN MAIT cells expressed noncanonical TRAV chains (Fig. 5B–E). The proportion of TRAV1-2^−^ TCRs increased after in vitro incubation with IL-2± *Mtb* lysates predominantly in CD4^+^ MAIT cells (Fig. S8A) and resembled private TCRs. CD4^+^ MAIT cells expressed *TRAV18-1*, *TRAV16*, *TRAV25*, *TRAV26-1*, *TRAV34*, *TRAV35*, *TRAV36*, *TRAV40*, and *TRAV8-2* (Kaur et al) and *TRAV29*, *TRAV22*, *TRAV30*, *TRAV35*, and *TRAV39* (Garner et al). Only *TRAV35^+^* CD4^+^ MAIT cells were observed in both datasets. A minority of CD8^+^ MAIT cells also expressed noncanonical TRAV chains including *TRAV1-1* and *TRAV39* (Kaur et al) and *TRAV10* and *TRAV9-1* (Garner et al) that were not observed in CD4^+^ MAIT cells. The majority of DN MAIT cells expressed shared noncanonical TRAVs with CD8^+^ and CD4^+^ MAIT cells in both datasets such as *TRAV21*, *TRAV8-1*, *TRAV12-2*, *TRAV12-3*, *TRAV41*, *TRAV13-1*, *TRAV14*, *TRAV20*, *TRAV17*, and *TRAV5* (Kaur et al and Garner et al). In the Kaur et al dataset, DN MAIT cells expressed 1 distinct TCR α chain, *TRAV19*, not observed in CD4^+^ or CD8^+^ MAIT cells (Fig. 5C, E). We also detected noncanonical α chains *TRAV8-1*, *TRAV21*, *TRAV12-2*, *TRAV12-3*, and *TRAV4* in MAIT cells that were identified in prior studies that used targeted single-cell TCR amplification (Fig. 5C, E).^31^^,^^37^

Next, we tested the hypothesis that in vitro stimulation with *Mtb* lysates could select for *TRAV1-2*^−^ chain usage and observed diverse expression of variable (V) and J segments (J) in the α chain in both IL-2± *Mtb* lysates conditions compared with ex vivo (Fig. S8A–D). *Mtb* lysate induction was associated with unique TCR usage including *TRAV34*, *TRAV35*, *TRAV1-1*, *TRAV8-2*, and *TRAV25* in predominantly CD4^+^ MAIT cells (Fig. S8A).

Additionally, we found that 112 MAIT cells in our dataset (9%) expressed dual TCR α chains (Fig. S9), consistent with one report.^26^ In contrast to that prior study in which all dual TCRα-expressing MAIT cells expressed at least 1 *TRAV1-2* chain, we found that 35% of dual TCRα-expressing MAIT cells had 2 isoforms of *TRAV1-2* chains with different J segments and CDR3α sequences, 40% coexpressed *TRAV1-2* with a different α chain, and 25% expressed 2 noncanonical α chains (Fig. S9A). The majority of CD8^+^ and DN MAIT cells expressed 2 *TRAV1-2* chains, whereas fewer CD4^+^ MAIT cells with dual TCRs expressed 1 *TRAV1-2* chain (Fig. S9A, B).

Next we analyzed TRBV chain expression in *TRAV1-2^+/-^* MAIT cells and found that 90% of *TRAV1-2*^+^ MAIT cells paired with canonical β chains such as *TRBV6-4*, *TRBV6-1*, or *TRBV20-1*, consistent with previous reports.^38^^,^^39^ The remaining 10% paired with noncanonical TRBV chains including *TRBV25-1*, *TRBV24-1*, *TRBV28*, and *TRBV11-1* (Fig. S10A). In contrast, *TRAV1-2*^−^ MAIT cells demonstrated significantly more diverse TRBV usage (Fig. S10B), resembling private TCRs.

**Figure 6.**
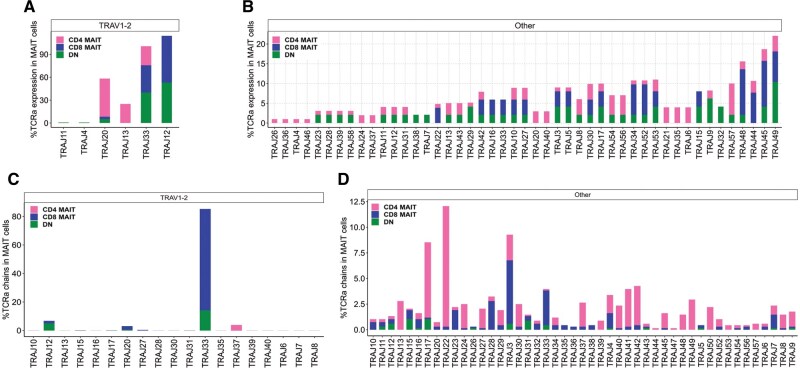
TCR J segment diversity in MAIT cell subsets sequenced in Kaur et al and Garner et al.^28^ Bar plot displaying the frequency of J segments in *TRAV1-2*^+^ (left) and *TRAV1-2*^−^ (right) MAIT cell subsets in Kaur et al (A, B) and Garner et al (C, D). Pink, blue, and green colors represent CD4^+^, CD8^+^, and DN MAIT cells, respectively.

Next, we analyzed MAIT cell J segment diversity and observed that 90% of *TRAV1-2^+^* MAIT cells had canonical J usage (*TRAJ-33/20/12*), as previously reported,^38^ whereas *TRAV1-2*^−^ MAIT cells demonstrated significantly more diverse J gene expression (Fig. 6), resembling private TCRs. While *TRAJ33* and *TRAJ12* were the predominant J chains expressed by *TRAV1-2*^+^ MAIT cells in both datasets, we found that CD4^+^  *TRAV1-2^+^* MAIT cells largely expressed *TRAJ33* and *TRAJ20* (Kaur et al) (Fig. 6A and C, Fig. S8B). Only 8% of *TRAV1-2*^−^ MAIT cells demonstrated canonical J gene expression (*TRAJ33/20/12*) in both datasets consistent with previous studies.^30^^,^^38^ CD4^+^  *TRAV1-2*^−^ MAIT cells also expressed more diverse J genes relative to CD8^+^ and DN *TRAV1-2^−^* MAIT cells (Fig. 6B). In vitro incubation with IL-2± *Mtb* lysates selected for noncanonical TRAJ usage (Fig. S8C), similar to our observations for TRAV selection (Fig. 5C). CD4^+^ and DN MAIT cells expressed various shared J segments such as *TRAJ8*, *TRAJ23*, *TRAJ28*, *TRAJ39*, *TRAJ9*, *TRAJ58*, *TRAJ57*, *TRAJ56*, *TRAJ54*, *TRAJ43*, *TRAJ11*, *TRAJ12*, *TRAJ13*, and *TRAJ29* while CD8^+^ and CD4^+^ MAIT cells only shared two J segments, *TRAJ22* and *TRAJ44*. We observed some J segments were commonly expressed in all three MAIT cell subsets, including *TRAJ30*, *TRAJ10*, *TRAJ53*, *TRAJ27*, *TRAJ42*, *TRAJ49*, *TRAJ3*, *TRAJ5*, *TRAJ48*, *TRAJ45*, *TRAJ34*, and *TRAJ52.* CD4^+^ MAIT cells expressed unique J genes including *TRAJ20*, *TRAJ21*, *TRAJ24*, *TRAJ26*, *TRAJ35*, *TRAJ36*, *TRAJ37*, *TRAJ4*, *TRAJ6*, *TRAJ40*, and *TRAJ46*, while DN *TRAV1-2*^−^ MAIT cells expressed more restricted chains including *TRAJ32*, *TRAJ38*, and *TRAJ7* (Kaur et al). CD8^+^ MAIT cells shared noncanonical *TRAJ* segments with CD4^+^ and DN MAIT cells, and we did not detect any unique J segment enrichment in CD8^+^  *TRAV1*-*2*^−^ MAIT cells (Fig. 6B; Fig. S8E, F). This contrasted with the Garner et al^30^ dataset in which CD4^+^ and CD8^+^  *TRAV1-2^−^* MAIT cells demonstrated similar J segment usage, likely explained by the study’s limited sequencing and analysis of *CD4*^+^ and *TRAV1-2*^−^ MAIT cells (Fig. 6D).

### *TRAV1-2* ^−^ MAIT cells have more variable CDR3α sequences

**Figure 7.**
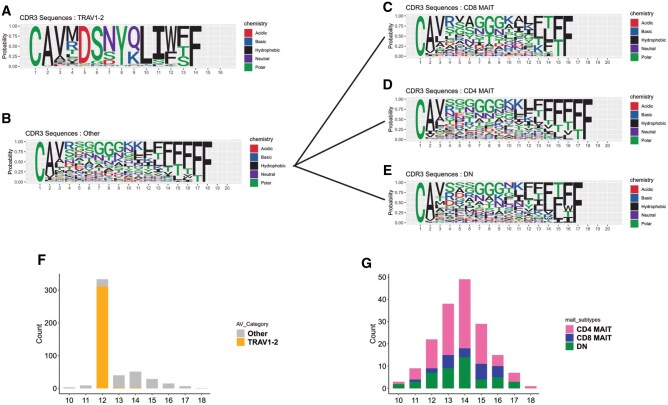
*TRAV1-2* ^−^ MAIT cell CDR3α diversity in Kaur et al. Sequence logo plots displaying the CDR3α sequences from Kaur et al expressed by (A) *TRAV1-2*^+^ MAIT cells, (B) *TRAV1-2*^−^ MAIT cells, and (C–E) MAIT cell subsets. (F) Bar plot displaying the length of CDR3α sequence in *TRAV1-2*^+/−^ MAIT cells in Kaur et al. (G) Bar plot displaying the amino acid length of CDR3α sequence in *TRAV1-2*^−^ MAIT cells stratified by subset in Kaur et al.

**Figure 8.**
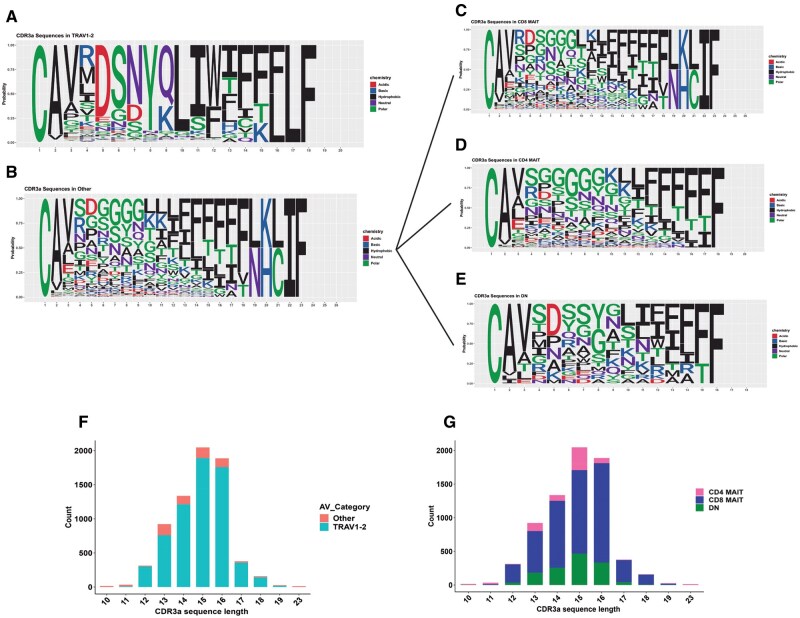
*TRAV1-2* ^−^ MAIT cell CDR3α diversity in Garner et al.^28^ Sequence logo plot displaying CDR3α sequences from Garner et al expressed by (A) *TRAV1-2*^+^ MAIT cells, (B) *TRAV1-2*^−^ MAIT cells, and (C–E) *TRAV1-2*^−^ MAIT cell subsets. (F) Bar plot displaying the length of CDR3α sequence stratified by MAIT cell subset in Garner et al. (G) Bar plot displaying the amino acid length of CDR3α sequence in *TRAV1-2*^−^ MAIT cell subsets in Garner et al.

Next, we compared the complementarity determining regions CDR3α and β of *TRAV1-2^+/-^* MAIT cells that confer antigen specificity to MR1-bound ligands. Ninety percent of *TRAV1-2*^+^ MAIT cells had CDR3α sequences that were 12 to 14 amino acids long with a conserved tyrosine (Y) residue at the 95th position in both datasets, as previously reported to be essential for MR1-presented riboflavin intermediate binding.^16^^,^^40^ In contrast, CDR3α sequences of *TRAV1-2*^−^ MAIT cells demonstrated more variation in the central region from positions 6 to 9, and about 25% of *TRAV1-2*^−^ MAIT cells from both datasets expressed triglycine (GGG) or tetraglycine (GGGG) motifs (Figs. 7A–E and 8A–E). There was also more variance in CDR3α sequence length from 7 to 23 amino acids in *TRAV1-2*^−^ MAIT cells (Fig. 7F, G and 8F, G). Despite variable lengths, the CDR3α sequences maintained conserved terminals consisting of cysteine (C) and alanine (A) at the first and second positions, respectively, with 60% to 70% containing valine (V) at the third position in both datasets. We found that the CDR3α sequences of all MAIT cells maintained conserved N-terminal amino acids including leucine (L), isoleucine (I), threonine (T), or phenylalanine (F). Next, we compared the CDR3α amino acid sequences of CD4^+^ and CD8^+^  *TRAV1-2*^−^ MAIT cells and observed that the CD4^+^ subset predominantly expressed tetraglycine (GGGG) and tetra-asparagine (NNNN) from the sixth to ninth position, whereas the CD8^+^ subset had triglycine (GGG) expression from seventh to ninth position (Figs. 7C–E and 8C–E). The CDR3α length of both CD4^+^ and CD8^+^  *TRAV1-2^−^* MAIT cells varied from 7 to 18 amino acids in Kaur et al dataset and up to 23 amino acids in the Garner et al^28^ dataset (Figs. 7F, G and 8F, G).

Next, we compared the CDR3β sequences and did not detect significant differences in CDR3β sequences between *TRAV1-2^+/-^* MAIT cells in either dataset (Figs. S11A, B and S12A, B). The CDR3β sequence of *TRAV1-2^+/-^* MAIT cells demonstrated conserved terminals consisting of cysteine (C) and alanine (A) at the first and second positions and a sequence 4 to 5 phenylalanine (F) residues at the N-terminal end, except 1 CDR3β sequence with extended length in Garner et al (Figs. S11A, B and S12A, B).^28^ We also compared the CDR3β sequences between MAIT cell subsets and again did not detect significant differences in CD4^+^, CD8^+^, or DN MAIT cells in either dataset (Figs. S11C–E and S12C–E). The majority of CDR3β chains of *TRAV1-2^+/-^* MAIT cells ranged between 14 to 16 amino acids (Figs. S11F, G and S12F, G).

### Functional annotation of MAIT cell TCRs and epitope prediction

As *TRAV1-2*^−^ CD4^+^ MAIT cells demonstrated unexpected TCR diversity, we hypothesized that these TCRs may bind alternative epitopes beyond canonical riboflavin intermediates. We performed similarity searches against a publicly available database using Mc-TCR software map (http://friedmanlab.weizmann.ac.il/McPAS-TCR/) to compare *TRAV1-2^+/-^* MAIT cell CDR3α and β sequence homology with a reference library. We found that CDR3α and β sequences of CD8^+^, CD4^+^, and DN *TRAV 1-2^+^* MAIT cells showed 100% similarity with published canonical CDR3α and β sequences associated with *Mtb* infection datasets,^39,40^ while CDR3α and β sequences of CD8^+^, CD4^+^, and DN *TRAV1-2^−^* MAIT cells had only 60% to 80% similarity at the central region with *Mtb*-specific MAIT cells, likely due to restricted definitions of MAIT cells as TRAV1-2^+^ T cells in prior studies.^41^^,^^42^ These *TRAV1-2*^−^ MAIT cells also demonstrated 60% to 80% similarity with peptide-specific epitopes derived from cytomegalovirus pp65 antigen and DQ2.5-glia antigen of celiac autoimmune disease signatures (Table S3). Modification of one amino acid in the CDR3α or CDR3β sequence in silico increased the similarity to >90% with published CDR3α and CDR3β sequences associated with *Mtb* infection. Modification of 2 amino acids in the CDR3α or CDR3β sequence further increased the similarity to 100% with published CDR3α and CDR3β sequences, that also included peptide cancer antigens hemoglobin-like protein (HbO), EphA2, NY-ESO-1, MART1, and T72 (Tn), hepatitis, COVID, cytomegalovirus, and Epstein-Barr, influenza and Yellow fever viral antigens (Table S3).^41–45^

**Figure 9.**
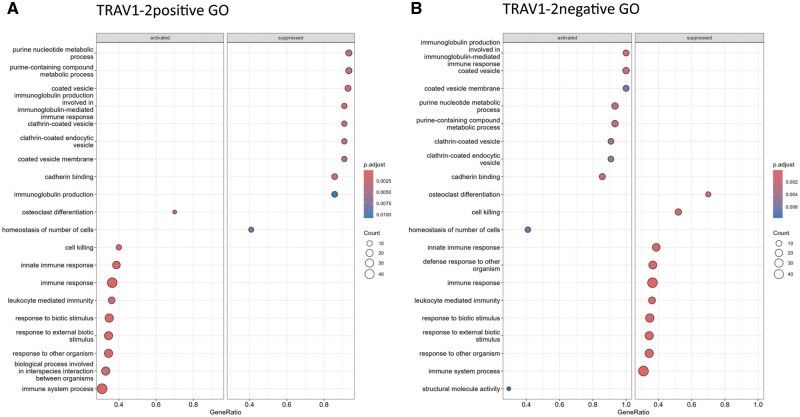
Functional annotations of *TRAV1-2*^+/−^ MAIT cells. Dot plot displaying predicted functional phenotypes in silico of (A) *TRAV1-2*^+^ and (B) *TRAV1-2*^*−*^ MAIT cells annotated in the Gene Ontology (GO) database using RStudio.

We next performed Gene Ontology annotations using KEGG and string networking analysis that demonstrated that *TRAV1-2*^+^ and *TRAV1-2*^−^ MAIT cells associated with different functional pathways (Fig. 9). Our analysis revealed that *TRAV1-2*^+^ MAIT cells expressed signatures of cell-mediated immunity whereas *TRAV1-2^−^* MAIT cells expressed genes involved in metabolic control and immunoglobulin production pathways.

## Discussion

Despite highly conserved CD8^+^TRAV1-2^+^ TCRs expressed by most human MR1-5-OP-RU–reactive MAIT cells, there is increasing appreciation of diverse TRAV1-2^−^ MAIT cells^40^ with distinct functional phenotypes.^1^^,^^3^^,^^28^ Our major finding in the present study is that the TCR coreceptor, CD4, is a major feature of these noncanonical MAIT cells in humans and that these CD4^+^ TRAV1-2^−^ MAIT cells resemble conventional T cells with private TCRs. Several outstanding questions remain about how CD4^+^ and other TRAV1-2^−^ MAIT cells are selected in vivo as they have lower affinity for riboflavin intermediates such as 5-OP-RU and are not frequently studied in animal models in which murine or macaque CD4 expression is less prevalent in mature MAIT cells and thought to be a thymic marker of immaturity in mice.^2^^,^^24^^,^^25^

Together, our data suggest that CD4^+^ MAIT cells may undergo post-thymic selection and warrant further study in epidemiologically relevant models.^1^^,^^3^^,^^28^^,^^46^ While canonical TRAV1-2^+^CD8^+^ MAIT cells are well studied and known to strongly bind and respond to intermediates of riboflavin metabolism through oligoclonal J (*TRAJ33/12/20*) and TRBV chain (*TRBV20/6*) usage,^47^ the TCR repertoire of TRAV1-2^−^ CD4^+/−^ and CD8^+/−^ MAIT cells is not well understood. To address this gap in knowledge, we employed unbiased single-cell TCR sequencing of total human blood MAIT cells defined by MR1-5-OP-RU tetramers and stratified analyses by CD4 and CD8 surface epitope mapping coupled to transcript expression. Our analyses revealed unexpected diversity in TCRα, J segment, CDR3α, and TCRβ usage enriched within CD4^+^*TRAV1-2*^−^ MAIT cells that was also detectable in CD8^+^ and DN MAIT cells at lower frequencies. This work presents the first unbiased analysis of human MAIT cell TCR diversity and provides evidence to support distinct TCR usage by noncanonical CD4^+^ TRAV1-2^−^ MAIT cells that indicates the potential for alternative ligand recognition beyond 5-OP-RU or other riboflavin precursor ligands.

While CD4^+^ MAIT cells compose a minor subset of MR1-5-OP-RU tetramer^+^ cells (e.g. 5-10%),^1^ our findings are of high clinical significance, as CD4^+^ MAIT cells are enriched during *Mtb* infection and cancer^1^^,^^3^^,^^28^^,^^46^^,^^48^ and express distinct costimulatory molecules including TNFRSF receptors. CD4^+^ MAIT cells also demonstrate constitutive expression of CD25 and the master regulatory transcription factor FOXP3, with striking resemblance to conventional CD4^+^ regulatory T cells at both the transcript and protein levels.^1^^,^^46^ Our results significantly advance prior work by elucidating the differences in antigen-specific binding sequences of TRAV1-2^−^ CD4^+^ MR1-5-OP-RU tetramer^+^ T cells that underlie their decreased reactivity to 5-OP-RU relative to TRAV1-2^+^CD8^+^ MAIT cells, but also their potential to recognize alternative antigens.^16^^,^^19^ While one study reported MR1-6-FP tetramer reactive TRAV1-2^−^ T cells with distinct binding geometry in the MR1 pocket compared with TRAV1-2^+^ MAIT cells,^16^ we could not detect significant expansion of TRAV1-2^+/−^ 5-OP-RU tetramer^+^ cells or MR1-6-FP tetramer^+^ cells in human blood with folate-derived MR1 ligands.

Our study also advances our understanding of TCR-dependent and TCR-independent activation of MAIT cells through stratification of analysis by CD4 and CD8 coexpression after in vitro stimulation.^49–51^ 5-OP-RU primarily induced CD8^+^ MAIT cell responses, while combined cytokine stimulation or nonspecific TCR stimulation drove distinct CD8^+^ and CD4^+^ MAIT cell responses. We highlight that CD4^+^ MAIT cells upregulated IL17-A, TNFα and GZMB after anti-CD3/CD28 stimulation, which was not observed in CD8^+^ MAIT cells and indicates that CD4^+^ MAIT cells may be more poised to respond to other ligands in addition to canonical riboflavin metabolites or TCR-independent signals.

Our study also provides important insights into an underappreciated and diverse TCR repertoire of human CD4^+^ and CD8^+^ MAIT cells that raises critical questions regarding how noncanonical TRAV1-2^−^ MAIT cells are selected. Despite distinctions in transcriptional signature and immune phenotype of CD4^+^ MAIT cells—including constitutive expression of *IL2RA* (CD25), *FOXP3*, *MAL*, *CTLA4*, and *TNFRSF4* during homeostasis relative to CD8^+^ and DN MAIT cells^1^—the CD4^+^ subset is not considered a distinct developmental lineage as defined by differential expression of transcription factors associated with conventional T cell ontogeny in the thymus (eg, *ThPOK*, *RUNX3*). Studies to date have concluded that CD4 is a marker of MAIT cell immaturity that is downregulated prior to thymic egress.^25^ However, the persistence of mature CD4^+^ MAIT cells in blood and tissues in humans, nonhuman primates, and mice indicates that they undergo post-thymic selection.^1^^,^^2^^,^^12^^,^^26^ A recent study in colorectal cancer found tumor-infiltrating MAIT cells enriched with CD4^+^FOXP3^+^CD39^+^ MAIT cells,^46^ which along with our studies during *Mtb* infection^1^ suggest specialized functions for CD4^+^ MAIT cells beyond a marker of early development or cytotoxicity that may be disease and tissue specific.

Prior structural modeling studies of noncanonical MAIT cell TCRs indicate alternative binding geometry and lower affinity for the MR1 receptor.^14^^,^^47^ The *TRAV1-2*^+^ MAIT cell TCR binds to the A′ pocket of the MR1 receptor, whereas TRAV1-2^−^ MAIT cell TCRs adopt alternative binding positions closer to the F’ pocket.^40^ These alternative binding models of noncanonical MAIT cell TCRs indicate that alternative ligands or costimulatory signals may be required to expand TRAV1-2^−^ MAIT cell clones, as they have lower affinity for riboflavin precursor antigens typically used to stimulate canonical TRAV1-2^+^CD8^+^ MAIT cells in vitro and in vivo.^1^^,^^2^

Importantly, a recent study further supports the clinical significance of TRAV1-2^−^ MAIT cells that were significantly associated with durable tuberculosis resistance.^28^ The authors reported that among all innate-like T cells assayed, only TRAV1-2^+/−^ MAIT cells significantly expanded in high-risk contacts of tuberculosis cases who never develop evidence for latent infection or active tuberculosis disease after long-term follow-up. While this study also observed that TRAV1-2^−^ MAIT cells resembled private TCRs, the investigators did not further interrogate TRAV1-2^−^ chain diversity or its association with CD4.^28^ We speculate that TRAV1-2^−^ MAIT cells with highly variable triglycine or tetraglycine (G) motifs enhance TCR promiscuity for other ligands independent of vitamin B biosynthesis and that this alternative ligand recognition may enhance the host response and protect against disease.^7^

We acknowledge the limitation of analyzing a small sample size of donors using CITE-Seq. We address this limitation by performing high-input sorted MAIT cell sequencing and integrating 2 independent datasets to support our conclusions and generalizability across distinct human donors and experimental settings. In the present study, we did not significantly expand CD4^+^ TRAV1-2^−^ MAIT cells for primary cell cloning or engineer cell lines for further functional validation. We also acknowledge that this study did not identify alternative classes of MR1 ligands that specifically induced CD4^+/−^ TRAV1-2^−^ MAIT cells. Future studies that address these limitations will be critical to understanding TRAV1-2^−^ MAIT cell selection and their role in MR1-restricted immunity.

Our ongoing work investigates human blood and tissue-specific CD4^+^ and CD8^+^ TRAV1-2^+/−^ MAIT cells across diverse human populations during healthy and disease states to inform the mechanisms by which noncanonical MAIT cells are selected.

## Supplementary Material

vkaf260_Supplementary_Data

## Data Availability

All data, code, and materials used in the analysis will be available to any researcher for purposes of reproducing or extending the analysis. Transcriptomic data has been deposited on the National Center for Biotechnology Information Gene Expression Omnibus database under the bioproject accession ID PRJNA1219683 and the Sequence Read Archive IDs SRR32309740 to SRR32309745.
