# Supplementary material for: CD4+ mucosal-associated invariant T cells express highly diverse T cell receptors
Source: J Immunol. 2025 Nov 9;214(12):3260–72. doi: 10.1093/jimmun/vkaf260 (PMC12726071; doi:10.1093/jimmun/vkaf260)
Supplement: vkaf260_Supplementary_Data [file vkaf260_supplementary_data.zip › vkaf260_Supplementary_Data/JI_Supplemental_Table2.pdf]

Supplemental Table 2: Differential gene expression analysis betweeen CD8^{+} (+ log2 fold change) and CD4^{+} (-log2 fold change) MAIT cells

	p_val	avg_log2FC	pct.1	pct.2	p_val_adj	genes
TRAV1-2	8.90E-57	4.588588	0.868	0.167	3.26E-52	TRAV1-2
SLC4A10	3.04E-47	3.738179	0.813	0.179	1.11E-42	SLC4A10
CTSW	2.99E-40	1.846581	0.959	0.643	1.09E-35	CTSW
KLRB1	6.31E-40	1.847105	0.964	0.53	2.31E-35	KLRB1
NKG7	1.41E-35	1.551821	0.976	0.732	5.16E-31	NKG7
NCR3	2.24E-35	2.19454	0.856	0.435	8.20E-31	NCR3
PRF1	3.19E-35	1.788724	0.935	0.583	1.17E-30	PRF1
MAL	1.20E-34	-2.40856	0.23	0.726	4.41E-30	MAL
COTL1	2.16E-30	-1.48587	0.746	0.952	7.91E-26	COTL1
CD4	9.53E-30	-2.4261	0.113	0.542	3.49E-25	CD4
ZBTB16	2.71E-28	2.849718	0.669	0.173	9.92E-24	ZBTB16
TRBV6-4	6.34E-27	4.815175	0.535	0.054	2.32E-22	TRBV6-4
LST1	6.73E-27	2.119348	0.803	0.429	2.46E-22	LST1
KLRG1	9.53E-27	2.803763	0.655	0.19	3.49E-22	KLRG1
AL136456.1	5.18E-25	2.010919	0.803	0.423	1.90E-20	AL136456.1
GZMA	1.07E-23	1.019275	0.983	0.792	3.90E-19	GZMA
HOPX	2.07E-23	1.131374	0.945	0.69	7.58E-19	HOPX
CST7	3.59E-23	1.097199	0.969	0.798	1.31E-18	CST7
CEBPD	1.43E-22	2.603052	0.633	0.196	5.22E-18	CEBPD
IKZF2	7.56E-22	2.379737	0.619	0.214	2.77E-17	IKZF2
CCL4	6.51E-21	2.162744	0.794	0.411	2.38E-16	CCL4
CCL3	1.09E-20	2.698654	0.724	0.363	3.97E-16	CCL3
CD8A	4.18E-20	1.235775	0.787	0.333	1.53E-15	CD8A
LAG3	5.89E-20	1.577714	0.837	0.601	2.15E-15	LAG3
PLEK	7.21E-20	1.942859	0.691	0.31	2.64E-15	PLEK
GNLY	1.21E-19	1.666099	0.923	0.714	4.41E-15	GNLY
ADAM12	1.26E-19	2.736983	0.53	0.131	4.61E-15	ADAM12
TNFRSF4	7.54E-18	-1.83563	0.463	0.738	2.76E-13	TNFRSF4
ARID5B	1.04E-17	-1.52793	0.319	0.655	3.81E-13	ARID5B
HPGD	2.11E-17	1.131856	0.693	0.327	7.74E-13	HPGD
GZMK	3.62E-17	1.465812	0.787	0.423	1.32E-12	GZMK
ZFP36L1	1.01E-16	1.196243	0.863	0.673	3.71E-12	ZFP36L1
NME2	1.83E-16	-0.8022	0.871	0.94	6.71E-12	NME2
RPS2	2.33E-16	-0.53702	1	1	8.52E-12	RPS2
LYAR	2.98E-16	1.587704	0.705	0.381	1.09E-11	LYAR
IL4I1	7.24E-16	1.613083	0.607	0.25	2.65E-11	IL4I1
GZMB	9.02E-16	1.038248	0.921	0.72	3.30E-11	GZMB
CD81	1.70E-15	0.776296	0.959	0.875	6.24E-11	CD81
RPS8	4.05E-15	-0.44475	1	1	1.48E-10	RPS8
LDHB	1.02E-14	-0.63695	0.928	0.964	3.75E-10	LDHB
LINC01871	1.30E-14	0.861828	0.923	0.744	4.77E-10	LINC01871
RPL8	3.83E-14	-0.35869	1	1	1.40E-09	RPL8

TMIGD2	4.38E-14	1.624688	0.559	0.214	1.60E-09	TMIGD2
CCR1	6.00E-14	1.85107	0.523	0.19	2.20E-09	CCR1
CCL5	8.08E-14	0.40291	0.971	0.732	2.96E-09	CCL5
CTSH	1.14E-13	1.194026	0.729	0.423	4.16E-09	CTSH
RPLP2	1.40E-13	-0.36188	1	1	5.13E-09	RPLP2
GBP5	2.34E-13	0.753383	0.894	0.75	8.55E-09	GBP5
ID2	2.67E-13	0.839094	0.902	0.744	9.76E-09	ID2
STAT1	3.42E-13	-1.05245	0.743	0.869	1.25E-08	STAT1
RORA	3.62E-13	0.840625	0.935	0.768	1.33E-08	RORA
SEC11C	4.13E-13	-1.1722	0.659	0.804	1.51E-08	SEC11C
CORO1B	5.28E-13	-1.24422	0.511	0.738	1.93E-08	CORO1B
RPS18	8.37E-13	-0.45446	1	1	3.06E-08	RPS18
LYST	1.83E-12	1.121799	0.794	0.548	6.68E-08	LYST
LINC00299	2.00E-12	1.329578	0.624	0.333	7.30E-08	LINC00299
TMSB10	3.20E-12	-0.54762	1	1	1.17E-07	TMSB10
TNF	4.62E-12	1.384895	0.7	0.429	1.69E-07	TNF
GYG1	9.39E-12	0.922433	0.837	0.667	3.44E-07	GYG1
RPL12	1.33E-11	-0.50395	1	1	4.88E-07	RPL12
PLCB1	1.81E-11	1.375482	0.602	0.292	6.63E-07	PLCB1
CXCR6	1.83E-11	0.768224	0.868	0.577	6.68E-07	CXCR6
SIRPG	2.51E-11	1.483811	0.561	0.268	9.18E-07	SIRPG
PRDX1	3.69E-11	-0.86747	0.878	0.946	1.35E-06	PRDX1
EEF2	5.04E-11	-0.51783	0.935	0.988	1.85E-06	EEF2
BTF3	8.15E-11	-0.38562	0.988	1	2.98E-06	BTF3
CD69	9.08E-11	0.747316	0.916	0.786	3.32E-06	CD69
LY6E	9.42E-11	-0.74948	0.863	0.935	3.45E-06	LY6E
TMEM173	1.18E-10	-1.32622	0.412	0.655	4.32E-06	TMEM173
RPS16	1.68E-10	-0.36996	1	1	6.14E-06	RPS16
RPS6	2.06E-10	-0.40762	1	1	7.54E-06	RPS6
PARP8	2.20E-10	0.966744	0.791	0.571	8.04E-06	PARP8
TXK	2.60E-10	1.159449	0.633	0.375	9.50E-06	TXK
KIAA0319L	3.75E-10	1.113741	0.652	0.429	1.37E-05	KIAA0319L
GSTP1	5.00E-10	-0.68559	0.787	0.905	1.83E-05	GSTP1
RBM3	7.71E-10	-0.61388	0.818	0.887	2.82E-05	RBM3
CMTM7	9.14E-10	-1.33877	0.264	0.506	3.35E-05	CMTM7
RPL18A	9.27E-10	-0.35726	1	1	3.39E-05	RPL18A
LINC01943	1.03E-09	-1.20997	0.446	0.673	3.79E-05	LINC01943
PTMS	1.12E-09	0.937632	0.767	0.595	4.11E-05	PTMS
RPL19	1.22E-09	-0.35328	1	1	4.46E-05	RPL19
LTB	1.26E-09	0.561497	0.998	1	4.60E-05	LTB
TSPO	1.32E-09	-0.73555	0.647	0.821	4.85E-05	TSPO
SYTL2	1.62E-09	0.778156	0.758	0.524	5.95E-05	SYTL2
SUPT3H	1.89E-09	1.002707	0.621	0.363	6.92E-05	SUPT3H
GBP1	2.27E-09	-0.99969	0.338	0.577	8.32E-05	GBP1
SUB1	3.17E-09	-0.51403	0.952	0.988	0.000116	SUB1
APOBEC3G	3.35E-09	0.769494	0.803	0.667	0.000122	APOBEC3G

PHACTR2	3.36E-09	1.051561	0.693	0.518	0.000123	PHACTR2
RPSA	3.84E-09	-0.38285	1	1	0.000141	RPSA
ALOX5AP	4.14E-09	0.504623	0.89	0.702	0.000151	ALOX5AP
CCNG2	5.08E-09	1.273223	0.513	0.25	0.000186	CCNG2
RPL4	5.81E-09	-0.46802	0.94	0.982	0.000213	RPL4
PITPNC1	5.96E-09	0.931527	0.734	0.5	0.000218	PITPNC1
RPL10A	7.77E-09	-0.35924	0.99	0.994	0.000284	RPL10A
CLEC2B	8.02E-09	0.581997	0.868	0.673	0.000294	CLEC2B
HMGB2	8.84E-09	1.317762	0.779	0.708	0.000324	HMGB2
ARL6IP5	1.09E-08	-0.48978	0.978	0.988	0.0004	ARL6IP5
ETFB	1.24E-08	-0.90845	0.518	0.685	0.000455	ETFB
MALAT1	1.26E-08	0.441939	1	1	0.000462	MALAT1
RPL22	1.28E-08	-0.37728	0.986	1	0.000467	RPL22
TIMP1	1.30E-08	-1.1432	0.338	0.565	0.000475	TIMP1
RUNX2	1.61E-08	1.002096	0.595	0.327	0.00059	RUNX2
NPM1	2.05E-08	-0.46062	0.964	0.994	0.000752	NPM1
MKI67	2.18E-08	1.746633	0.523	0.315	0.000799	MKI67
RPS11	2.20E-08	-0.39957	0.976	1	0.000805	RPS11
RGS1	2.32E-08	-1.23533	0.353	0.571	0.00085	RGS1
RPL5	2.51E-08	-0.40822	0.99	0.988	0.000918	RPL5
PIP4K2A	2.62E-08	0.840976	0.739	0.542	0.000957	PIP4K2A
SMAP2	2.87E-08	-0.7363	0.703	0.833	0.00105	SMAP2
ARL3	3.74E-08	1.009834	0.561	0.315	0.001371	ARL3
COX6B1	4.18E-08	-0.36224	0.957	0.994	0.001532	COX6B1
LAT	6.11E-08	-0.70832	0.775	0.845	0.002235	LAT
DDIT4	7.17E-08	-1.16669	0.616	0.768	0.002625	DDIT4
DNAJC1	1.01E-07	0.753641	0.77	0.631	0.00368	DNAJC1
CELF2	1.05E-07	0.623962	0.918	0.833	0.003844	CELF2
PTPN22	1.19E-07	0.677932	0.753	0.589	0.004362	PTPN22
SUSD3	1.25E-07	-1.0229	0.305	0.512	0.004581	SUSD3
LCP1	1.34E-07	0.439917	0.964	0.905	0.004916	LCP1
ISG20	1.47E-07	-1.12747	0.528	0.69	0.005384	ISG20
PSMA2	1.66E-07	-0.45478	0.813	0.917	0.006063	PSMA2
PHLDA1	1.68E-07	0.931302	0.621	0.405	0.006162	PHLDA1
HCST	2.11E-07	0.422512	0.988	0.964	0.007718	HCST
GAB3	2.17E-07	1.021031	0.501	0.274	0.007931	GAB3
EEF1G	3.13E-07	-0.36926	0.969	0.988	0.011457	EEF1G
TRAPPC1	3.45E-07	-0.40376	0.923	0.923	0.012624	TRAPPC1
HSPD1	3.49E-07	-0.74961	0.65	0.78	0.012761	HSPD1
FKBP1A	3.70E-07	-0.57144	0.779	0.863	0.013552	FKBP1A
MYO1F	3.92E-07	0.580405	0.791	0.607	0.014365	MYO1F
CBLB	4.06E-07	0.662562	0.791	0.643	0.01487	CBLB
ITGB2	4.09E-07	0.559678	0.892	0.792	0.014961	ITGB2
DDX39A	5.09E-07	0.886913	0.655	0.476	0.01863	DDX39A
RPL9	5.90E-07	-0.40578	0.998	1	0.021584	RPL9
SLC25A3	5.96E-07	-0.4494	0.866	0.917	0.021802	SLC25A3

IL2RA	6.01E-07	-0.90817	0.604	0.762	0.021992	IL2RA
TNFRSF1B	6.03E-07	-0.78365	0.76	0.821	0.022086	TNFRSF1B
TRG-AS1	6.21E-07	0.828521	0.65	0.464	0.022745	TRG-AS1
TUBA1A	6.32E-07	0.825737	0.679	0.524	0.023126	TUBA1A
GSTK1	6.97E-07	-0.46684	0.916	0.935	0.025505	GSTK1
ENTPD1	7.23E-07	0.791855	0.7	0.565	0.026467	ENTPD1
FYB1	7.56E-07	-0.66052	0.751	0.875	0.027685	FYB1
ATP8B4	7.78E-07	1.059799	0.58	0.375	0.028477	ATP8B4
ANK3	9.25E-07	-0.97908	0.336	0.53	0.033865	ANK3
EFHD2	1.22E-06	0.518594	0.868	0.792	0.044731	EFHD2
MAPRE2	1.23E-06	0.856832	0.566	0.357	0.045055	MAPRE2
CKLF	1.32E-06	0.374091	0.974	0.929	0.04822	CKLF
LINC01138	1.50E-06	0.73088	0.679	0.482	0.054934	LINC01138
CCDC107	1.58E-06	0.599097	0.779	0.619	0.058004	CCDC107
S100A11	1.64E-06	-0.36144	0.988	0.976	0.060085	S100A11
SOS1	1.74E-06	-0.60864	0.76	0.851	0.063714	SOS1
NDUFS5	1.75E-06	-0.43506	0.868	0.911	0.064086	NDUFS5
KLRK1	2.04E-06	0.789879	0.635	0.423	0.074582	KLRK1
VDAC1	2.07E-06	-0.5121	0.741	0.815	0.075672	VDAC1
EEF1B2	2.30E-06	-0.46084	0.957	0.994	0.084285	EEF1B2
TUBA1B	2.34E-06	1.526324	0.813	0.756	0.085714	TUBA1B
RNF157	2.39E-06	0.875367	0.59	0.399	0.087489	RNF157
PABPC1	2.60E-06	-0.46388	0.918	0.97	0.095321	PABPC1
ACTR3	2.67E-06	-0.45367	0.873	0.946	0.097778	ACTR3
NFKBIA	2.90E-06	0.445794	0.918	0.845	0.106118	NFKBIA
PSMB4	3.26E-06	-0.61798	0.652	0.762	0.119439	PSMB4
RPS6KA3	3.35E-06	0.662391	0.767	0.601	0.122438	RPS6KA3
DPP4	3.58E-06	0.656536	0.657	0.452	0.130974	DPP4
COX5B	3.85E-06	-0.36956	0.926	0.946	0.140946	COX5B
LMAN1	4.04E-06	-0.61345	0.638	0.786	0.147696	LMAN1
BCL7C	4.40E-06	0.676105	0.691	0.518	0.161178	BCL7C
JAK1	4.42E-06	0.385853	0.942	0.899	0.161923	JAK1
PRR13	4.45E-06	-0.3605	0.885	0.935	0.162809	PRR13
UBE2S	4.82E-06	1.345029	0.609	0.5	0.176319	UBE2S
TUBB	5.34E-06	1.306603	0.868	0.839	0.195413	TUBB
TGFBR2	5.68E-06	0.756435	0.643	0.452	0.20775	TGFBR2
CISH	5.71E-06	-0.88656	0.441	0.583	0.20907	CISH
CTSD	5.80E-06	0.519443	0.861	0.726	0.212137	CTSD
FAM3C	5.89E-06	0.86022	0.54	0.327	0.215505	FAM3C
TAGLN2	6.09E-06	-0.44035	0.911	0.94	0.222943	TAGLN2
MKNK2	6.11E-06	-0.8801	0.384	0.565	0.223689	MKNK2
CCND2	6.20E-06	-0.88518	0.71	0.804	0.227008	CCND2
CARS	6.52E-06	-0.95703	0.355	0.518	0.23849	CARS
RPS17	6.52E-06	-0.52034	0.866	0.887	0.23877	RPS17
CDC42EP3	6.92E-06	0.761634	0.58	0.375	0.253245	CDC42EP3
SAMD3	7.15E-06	0.796737	0.588	0.405	0.261614	SAMD3

MDH2	7.40E-06	-0.51403	0.7	0.798	0.270876	MDH2
C1QBP	7.61E-06	-0.76062	0.511	0.637	0.278523	C1QBP
H2AFV	8.09E-06	0.551382	0.897	0.875	0.296121	H2AFV
TMPO	8.44E-06	1.015891	0.607	0.488	0.309035	TMPO
HP1BP3	8.79E-06	0.535376	0.82	0.685	0.321577	HP1BP3
TRAF3IP3	8.80E-06	-0.42343	0.82	0.887	0.322065	TRAF3IP3
ARL6IP1	8.87E-06	0.742887	0.918	0.905	0.324507	ARL6IP1
PPA1	9.83E-06	-0.81697	0.54	0.649	0.359642	PPA1
PYHIN1	1.03E-05	0.55153	0.719	0.536	0.375297	PYHIN1
NAP1L4	1.14E-05	-0.52469	0.794	0.899	0.417065	NAP1L4
SSBP1	1.35E-05	-0.47799	0.71	0.792	0.494775	SSBP1
H1FX	1.40E-05	0.537649	0.902	0.857	0.513615	H1FX
CHD9	1.44E-05	0.824993	0.535	0.351	0.525353	CHD9
FAF1	1.47E-05	0.756253	0.633	0.47	0.538365	FAF1
TAGAP	1.47E-05	0.845765	0.561	0.381	0.538858	TAGAP
NUCB2	1.52E-05	0.830041	0.547	0.369	0.556214	NUCB2
SNU13	1.64E-05	-0.48674	0.794	0.851	0.602026	SNU13
FAM174C	1.66E-05	-0.67162	0.429	0.601	0.60847	FAM174C
SIPA1L1	1.80E-05	0.91222	0.585	0.411	0.658157	SIPA1L1
IER5	1.96E-05	0.599805	0.614	0.423	0.717293	IER5
NCALD	1.97E-05	0.515458	0.676	0.494	0.719834	NCALD
COX14	1.99E-05	-0.58258	0.604	0.756	0.728862	COX14
FKBP11	2.04E-05	0.468904	0.861	0.756	0.746837	FKBP11
SMC4	2.04E-05	1.163487	0.549	0.446	0.747089	SMC4
RNASET2	2.11E-05	-0.79365	0.434	0.583	0.770535	RNASET2
STMN1	2.23E-05	1.104935	0.693	0.601	0.816626	STMN1
EVA1B	2.31E-05	0.795181	0.513	0.345	0.847104	EVA1B
TALDO1	2.34E-05	-0.55171	0.763	0.821	0.855965	TALDO1
MRPS6	2.36E-05	0.446627	0.791	0.696	0.863321	MRPS6
TUBA4A	2.41E-05	0.726114	0.727	0.637	0.880547	TUBA4A
OCIAD2	2.41E-05	-0.53078	0.715	0.798	0.882847	OCIAD2
UBE2F	2.52E-05	-0.92164	0.405	0.554	0.920923	UBE2F
SLFN12L	2.53E-05	0.625987	0.578	0.399	0.927476	SLFN12L
DLEU2	2.62E-05	0.702018	0.612	0.452	0.960417	DLEU2
BUB3	2.71E-05	0.602424	0.775	0.685	0.993532	BUB3
