# Supplementary material for: CD4+ mucosal-associated invariant T cells express highly diverse T cell receptors
Source: J Immunol. 2025 Nov 9;214(12):3260–72. doi: 10.1093/jimmun/vkaf260 (PMC12726071; doi:10.1093/jimmun/vkaf260)
Supplement: vkaf260_Supplementary_Data [file vkaf260_supplementary_data.zip › vkaf260_Supplementary_Data/JI_Supplemental_Table3.pdf]

Supplemental Table 3: CDR3α/β sequence similarity with published CDR3 sequences of T cells.

**TRAV1-2 negative CDR3alpha sequence similarity**																	
Query	Dist	CDR3.alpha.aa	Pathology	Pathology.Mesh.ID	Single.cell	Antigen.protein	Epitope.peptide	Epitope.ID	Tissue	T.Cell.Type	T.cell.characteristics	TRAV	TRAJ	TRBV	TRBD	TRBJ	PubMed.ID
CAVARSGGYQKVTF	2	CAASGGYQKVTF	Human immunodeficiency virus (HIV)	D006782	No	Gag p24	KAFSPEVIPMF	29804	PBMC	CD8	NA	TRAV5	NA	TRBV19	NA	TRBJ 1-2	17121793
CAEPGRRALTF	2	CAVSEPGRRALTF	M.Tuberculosis	D009169	Yes	NA	NA	NA	PBMC	CD4	NA	TRAV8-4	TRAJ5	TRBV29-1	NA	TRBJ 2-5	32341563
CAMSAGTGNQFYF	2	CAASGGTGNQFYF	Influenza	D009980	Yes	Matrix protein (M1)	GILGFVFTL	20354	Bronchoalveolar	CD8	NA	TRAV29/DV5:01	TRAJ4-01	TRBV1-01	NA	TRBJ 2-7:01	28636592
CLVRFGGFKTIF	2	CAVRGGGFKTIF	Parkinson disease	D010301	Yes	NA	NA	NA	CSF	CD8	NA	NA	TRAJ9	TRBV2	NA	TRBJ 2-3	31915375
CAENNAGKSTF	2	CAVNAGKSTF	Yellow fever virus	D015005	Yes	YFV-17D	LLWNGPMAV	121572	NA	CD8	NA	TRAV12-2	TRAJ27	TRBV6-06	TRBD1, TRBD2	TRBJ 2-7	28103239
CATDKAGTALIF	1	CATDAKAGTALIF	Human immunodeficiency virus (HIV)	D006678	No	RF10 Protein Nef	RYPLTFGWCF	56620	PBMC	CD8	NA	TRAV17	TRAJ15	TRBV28	TRBD2	TRBJ 2-2	24899498
CAVRDRDGGFKTIF	2	CAVRDDTGGFKTIF	Melanoma	D008545	Yes	GP100-IMD	IMDQVPFSV	27469	NA	NA	NA	NA	NA	TRBV19	NA	NA	30418433
CALDMMDSNYQLIW	2	CAADMDSNYQLIW	Influenza	D009980	Yes	PB1	LSLRNPILV	39494	Bronchoalveolar	CD8	NA	TRAV5N-4:01	TRAJ3-01	TRBV1-01	NA	TRBJ 1-1:01	28636592
CAVSGGGFKTIF	2	CAVNTGGFKTIF	Melanoma	D008545	No	Melan-A/MART-1	EAAGIGILTV	10987	Tumor Tissue	NA	NA	TRAV2	TRAJ9	NA	NA	NA	7777568
CAFSTAFGNEKLTF	2	CAFSTAAGNKLTF	Neoantigen	C050269	Yes	BAIAP3-ILN	ILNVDVFTL	606794	NA	NA	NA	TRAV5	NA	TRBV30	NA	NA	30418433
CAVTNQAGTALIF	2	CAVVDQAGTALIF	Cytomegalovirus (CMV)	D003586	Yes	pp65	NLVPMVATV	44920	PBMC	CD8	NA	TRAV2-01	TRAJ1-01	TRBV4-01	NA	TRBJ 2-1:01	28636592

CAVTLVW	2	CAVELVF	Neoantigen	C050269	Yes	EXOC3L4-ILL	ILLDWAANV	NA	NA	NA	NA	TRAV14/DV4	NA	TRBV7-9	NA	NA	30418433
CARRSGGGADGLTF	2	CAVNSGGGADGLTF	Influenza	D009980	Yes	Matrix protein (M1)	GILGFVFTL	20354	PBMC	CD8	NA	TRAV21	NA	TRBV6-1	NA	NA	28636589
CAANSSASKIIF	2	CAEYSSASKIIF	Epstein Barr virus (EBV)	D020031	No	BMLF-1	GLCTLVAML	20788	PBMC	CD8	NA	TRAV15	TRAJ3	TRBV14	NA	TRBJ2-3	10925283
CASRRPNTGNQFYF	2	CAYRSPNTGNQFYF	M.Tuberculosis	D009169	Yes	NA	NA	NA	PBMC	CD4	NA	mTRDV2-2	TRAJ49	TRBV11-3	NA	TRBJ2-1	32341563
CAFMKPNYGGATNKLIF	2	CAFLPPNYGGATNKLIF	M.Tuberculosis	D009169	Yes	NA	NA	NA	PBMC	CD4	NA	mTRDV2-2	TRAJ32	TRBV5-1	NA	TRBJ1-2	32341563
CAVVSYSGGGADGLTF	2	CAVPYSGGGADGLTF	Influenza	D009980	Yes	Matrix protein (M1)	GILGFVFTL	20354	PBMC	CD8	NA	TRAV12-2:01	TRAJ4-01	TRBV2-01	NA	TRBJ2-5:01	28636592
CAVSVAGGGADGLTF	2	CAVSRGGGADGLTF	Melanoma	D008545	No	Melan-A/MART-1	EAAGIGILTV	10987	Tumor Tissue	NA	NA	TRAV2	TRAJ45	NA	NA	TRBJ1-5	7777568
CATASSGNTPLVF	2	CAETPSSGNTPLVF	Neoantigen	C050269	Yes	OR6F1-VLN	VLNPFIYTL	NA	NA	NA	NA	TRAV12-2	NA	TRBV4-1	NA	NA	30418433
CAVNVTGGGNKLTF	2	CAVIFTGGGNKLTF	Human immunodeficiency virus (HIV)	D006678	No	RF10 Protein Nef	RYPLTFGWCF	56620	PBMC	CD8	NA	TRAV8-1	TRAJ10	TRBV7-09	TRBD1	TRBJ2-5	24899498
CAVSDTNAGKSTF	2	CAVRDTNTGKSTF	Herpes simplex virus 2 (HSV2)	D018258	No	HSV-2 VP22	RPRGEVRFL	55320	PBMC	CD8	NA	TRAV19	NA	TRBV9	NA	TRBJ2-1	20139278
CALKRQGGKLIF	2	CALSNQGGKLIF	Influenza	D009980	Yes	Matrix protein (M1)	GILGFVFTL	20354	PBMC	CD8	NA	TRAV19	NA	TRBV19	NA	NA	28636589
CAYRSARVKTSGSRLTF	2	CAYRSARTSGSRLTF	M.Tuberculosis	D009169	Yes	NA	NA	NA	PBMC	CD4	NA	mTRDV2-2	TRAJ58	TRBV7-2	NA	TRBJ1-4	32341563
CAESRMAAGNKLTF	2	CAASMAAGNKLTF	HTLV-1	D015368	No	NA	NA	NA	PBMC	CD4	NA	NA	TRAJ17	NA	TRBD1	TRBJ2-3	31069115
CAVQLGENAGNMLTF	2	CAVQGANAGNMLTF	M.Tuberculosis	D009169	Yes	NA	NA	NA	PBMC	CD4	NA	TRAV2	TRAJ39	TRBV4-1	NA	TRBJ1-4	32341563

CVVRALSGAGSYQLTF	2	CVVVAYSGAGSYQLTF	Cytomegalovirus (CMV)	D003586	Yes	pp65	NLVPMVATV	44920	PBMC	CD8	NA	TRAV10	NA	TRBV7-2	NA	NA	28636589
CASSYNTDKLIF	2	CAVSGSYNTDKLIF	Cytomegalovirus (CMV)	D003586	Yes	pp65	NLVPMVATV	44920	PBMC	CD8	NA	TRAV8-4:01	TRAJ3-01	TRBV2-01	NA	TRBJ 2-7:01	28636592
CVVRVTGGGNKLTF	2	CAVRRTGGGNKLTF	M.Tuberculosis	D009169	Yes	NA	NA	NA	PBMC	CD4	NA	TRAV3	TRAJ10	TRBV3-1	NA	TRBJ 1-2	32341563
CAASAPGGTSYGKLTF	1	CAASASGGTSYGKLTF	Influenza	D009980	Yes	Matrix protein (M1)	GILGFVFTL	20354	PBMC	CD8	NA	NA	NA	TRBV6-5:01	TRBD	TRBJ 2-3:01	28300170
CAVGVVGSQGNLIF	2	CAVGGSQGNLIF	Influenza	D009980	Yes	Matrix protein (M1)	GILGFVFTL	20354	Bronchoalveolar	CD8	NA	TRAV8-6:02	TRAJ4-01	TRBV1-01	NA	TRBJ 2-7:01	28636592
CATGDSKLTF	2	CAGGDSTLTF	Yellow fever virus	D015005	Yes	YFV-17D	LLWNGPMAV	121572	NA	CD8	NA	TRAV12-2	TRAJ11	TRBV3-01	TRBD1, TRBD2	TRBJ 1-5	28103239
CAVNKAAGNKLTF	2	CVVFKAAGNKLTF	Epstein Barr virus (EBV)	D020031	Yes	BMLF-1	GLCTLVAML	20788	PBMC	CD8	NA	TRAV10-01	TRAJ1-01	TRBV1-01	NA	TRBJ 2-5:01	28636592
CAMREVNSGGYQKVTF	2	CAMRDLNSGGYQKVTF	Neoantigen	C050269	Yes	PGM5-AVG-H5Y	AVGSYVYSV	NA	NA	NA	NA	TRAV12-2	NA	TRBV11-2	NA	NA	30418433
CAVEENDYKLSF	2	CAVSSNDYKLSF	Yellow fever virus	D015005	Yes	YFV-17D	LLWNGPMAV	121572	NA	CD8	NA	TRAV12-2	TRAJ20	TRBV6-01	TRBD2	TRBJ 1-5	28103239
CAVSLNRDDKIIF	2	CAVRNRDDKIIF	Neoantigen	C050269	Yes	WDR46	FLTYLDVSV	NA	NA	NA	NA	NA	NA	TRBV9	NA	NA	30418433
CAVPHNFGNEKLTF	2	CAVRANFGNEKLTF	Influenza	D009980	Yes	Matrix protein (M1)	GILGFVFTL	20354	PBMC	CD8	NA	TRAV41:01	TRAJ4-01	TRBV6-01	NA	TRBJ 2-2:01	28636592
CAMSERGFGNVLHC	2	CAVSEGFGNVLHC	M. tuberculosis	D009169	Yes	NA	NA	NA	PBMC	CD8	NA	NA	TRAJ35	TRBV20-1	NA	TRBJ 2-7	30992377
CATHNTDKLIF	2	CAALNTDKLIF	Diabetes Type 1	D003922	No	GAD65	NFIRMVISNPAAT	101069	PBMC	CD4	NA	NA	NA	NA	NA	TRBJ 1-1	25681349
CAVGSAGGTSYGKLTF	2	CAMSSAGGTSYGKLTF	Epstein Barr virus (EBV)	D020031	Yes	BMLF-1	GLCTLVAML	20788	PBMC	CD8	NA	TRAV12-3	NA	TRBV14	NA	NA	28636589
CAVNGLGFGNVLHC	2	CAVEDLGFGNVLHC	Neoantigen	C050269	Yes	SEC24A	FLYNPLTRV	NA	NA	NA	NA	TRAV12-3	NA	TRBV28	NA	NA	30418433

CAVNVVGTSYGKLTF	2	CAVNAGGTSYGKLTF	Diabetes Type 1	D003922	Yes	NA	NA	NA	Pancreatic islets	CD4	NA	TRAV12-2	TRAJ52	TRBV6-5	NA	TRBJ 1-1	27920090
CAVGNQGGSEKLVF	2	CAGTQGGSEKLVF	Tumor	D009370	No	NY-ESO-1	NA	NA	PBL	CD4	NA	TRAV38-1	TRAJ57	TRBV2	NA	TRBJ 2-3	30530988
CAVTPRANDYKLSF	2	CAVPRNDYKLSF	Neoantigen	C050269	Yes	OR14C36-FML-V6L	FMLYLLTLM	NA	NA	NA	NA	TRAV19	NA	TRBV5-1	NA	NA	30418433
CAVRGSGSARQLTF	2	CAVSGSARQLTF	Yellow fever virus	D015005	Yes	YFV-17D	LLWNGPMAV	121572	NA	CD8	NA	TRAV12-2	TRAJ22	TRBV9	TRBD1, TRBD2	TRBJ 2-7	28103239
CATDASGYSTLTF	2	CATLSGYSTLTF	M.Tuberculosis	D009169	Yes	NA	NA	NA	PBMC	CD4	NA	TRAV17	TRAJ11	TRBV25-1	NA	TRBJ 2-2	32341563
CACYDMNRDDKIIF	2	CADDMNRDDKIIF	Neoantigen	C050269	Yes	FLNA-HIA	HIAKSPFEV	NA	NA	NA	NA	NA	NA	TRBV6-2,6-3	NA	NA	30418433
CAVKRGNEKLTF	2	CAVTGNEKLTF	Yellow fever virus	D015005	Yes	YFV-17D	LLWNGPMAV	121572	NA	CD8	NA	TRAV12-2	TRAJ48	TRBV6-05	TRBD2	TRBJ 2-7	28103239
CALTKASGGSNYKLTF	2	CALTQNSGGSNYKLTF	M.Tuberculosis	D009169	Yes	NA	NA	NA	PBMC	CD4	NA	TRAV9-1	TRAJ53	TRBV20-1	NA	TRBJ 1-2	32341563
CAVCNNNDMRF	2	CAVTRNNDMRF	Yellow fever virus	D015005	Yes	YFV-17D	LLWNGPMAV	121572	NA	CD8	NA	TRAV12-2	TRAJ43	TRBV5-04	TRBD2	TRBJ 2-7	28103239
CALCPTGANSKLTF	2	CATGPTGANSKLTF	M.Tuberculosis	D009169	Yes	NA	NA	NA	PBMC	CD4	NA	TRAV17	TRAJ56	TRBV20-1	NA	TRBJ 1-4	32341563
CALSGGSNYKLTF	2	CAVSGGINYKLTF	Tumor	D009369	No	T72(Tn)	VITAFTEGLK	693671	NA	NA	NA	TRAV1	TRAJ45	TRBV1	NA	TRBJ 2-1	10508250
CLLATYSGAGSYQLTF	2	CAATYSGAGSYQLTF	M. tuberculosis	D009169	Yes	NA	NA	NA	PBMC	CD8	NA	NA	TRAJ28	TRBV2	NA	TRBJ 2-3	30992377
CAVSEWGGSEKLVF	2	CAVSSGGGSEKLVF	M.Tuberculosis	D009169	Yes	NA	NA	NA	PBMC	CD4	NA	TRAV21	TRAJ57	TRBV3-1	NA	TRBJ 1-6	32341563
CLVGSLSGGYNKLIF	2	CLVGDSGGYNKLIF	M.Tuberculosis	D009169	Yes	NA	NA	NA	PBMC	CD4	NA	TRAV4	TRAJ4	TRBV20-1	NA	TRBJ 2-2	32341563
CAFTTNTGNQFYF	2	CATKNTGNQFYF	Cytomegalovirus (CMV)	D003586	No	pp65	NLVPMVATV	44920	PBMC	CD8	NA	TRAV2-2	TRAJ49	TRBV14	NA	TRBJ 2-3	16237109
CAVSSNRDDKIIF	2	CAVRNRDDKIIF	Neoantigen	C050269	Yes	WDR46	FLTYLDVSV	NA	NA	NA	NA	NA	NA	TRBV9	NA	NA	30418433
CAVLYSGNTGKLIF	2	CAVDGSGNTGKLIF	Diabetes Type 1	D003922	Yes	NA	NA	NA	Pancreatic islets	CD4	NA	TRAV2	TRAJ37	TRBV4-1	NA	TRBJ 2-7	27920090

CATDEGAQKLVF	2	CATDHQGAQKLVF	Yellow fever virus	D015005	Yes	YFV-17D	LLWNGPMAV	121572	NA	CD8	NA	TRAV17	TRAJ54	TRBV9	TRBD2	TRBJ2-1	28103239
CASFRIQGAQKLVF	2	CAFIQGAQKLVF	Influenza	D009980	Yes	Matrix protein (M1)	GILGFVFTL	20354	PBMC	CD8	NA	TRAV38-1	NA	TRBV27	NA	NA	28636589
CAALPYSGGGADGLTF	2	CAVPYSGGGADGLTF	Influenza	D009980	Yes	Matrix protein (M1)	GILGFVFTL	20354	PBMC	CD8	NA	TRAV12-2:01	TRAJ4-01	TRBV2-01	NA	TRBJ2-5:01	28636592
CAATNTGTASKLTF	2	CALNTGTASKLTF	Neoantigen	C050269	Yes	NSDHL-A9V	KLVALGINAV	NA	NA	NA	NA	TRAV38-2/DV8	NA	TRBV7-8	NA	NA	30418433
CANGGGADGLTF	2	CAVNSGGGADGLTF	Influenza	D009980	Yes	Matrix protein (M1)	GILGFVFTL	20354	PBMC	CD8	NA	TRAV21	NA	TRBV6-1	NA	NA	28636589
CAVIPSGGYQKVTF	2	CAVSGGYQKVTF	Human immunodeficiency virus (HIV)	D006783	No	Gag p24	KAFSPEVIPMF	29804	PBMC	CD8	NA	TRAV5	NA	TRBV19	NA	TRBJ1-2	17121793
CAMREGVTGNQFYF	2	CAMREGLQTGNQFYF	M. tuberculosis	D009169	Yes	NA	NA	NA	PBMC	CD8	NA	NA	TRAJ49	TRBV6-1	NA	TRBJ2-7	30992377
CAMSEAAGNKLTF	2	CAFSTAAGNKLTF	Neoantigen	C050269	Yes	BAIAP3-ILN	ILNVDVFTL	606794	NA	NA	NA	TRAV5	NA	TRBV30	NA	NA	30418433
CALVYSSASKIIF	2	CAEYSSASKIIF	Epstein Barr virus (EBV)	D020031	No	BMLF-1	GLCTLVAML	20788	PBMC	CD8	NA	TRAV15	TRAJ3	TRBV14	NA	TRBJ2-3	10925283
CAFMRGAQKLVF	1	CAFGRGAQKLVF	Herpes simplex virus 2 (HSV2)	D018258	No	HSV-2 VP22	RPRGEVRFL	55320	PBMC	CD8	NA	TRAV5	NA	TRBV30	NA	TRBJ1-4	20139278
CAVNLQGAQKLVF	2	CAVIIQGAQKLVF	Tumor	D009370	No	NY-ESO-1	NA	NA	PBL	CD4	NA	TRAV12-2	TRAJ54	TRBV2	NA	TRBJ1-5	30530988
CGAAFDSWGKLQF	2	CAATDSWGKLQF	Yellow fever virus	D015005	Yes	YFV-17D	LLWNGPMAV	121572	NA	CD8	NA	TRAV25	TRAJ24	TRBV9	TRBD1, TRBD2	TRBJ2-1	28103239
CAVRYNNAGNMLTF	2	CAVREDNAGNMLTF	Cytomegalovirus (CMV)	D003586	Yes	pp65	NLVPMVATV	44920	PBMC	CD8	NA	TRAV1-2:01	TRAJ3-01	TRBV1-01	NA	TRBJ2-1:01	28636592
CAVNAYNDMRF	2	CAVNMGNDMRF	Neoantigen	C050269	Yes	MRM1-9	LLFGMTPCL	NA	NA	NA	NA	NA	NA	TRBV4-2	NA	NA	30418433

CAVSPPGSARQLTF	2	CAVSGSARQLTF	Yellow fever virus	D015005	Yes	YFV-17D	LLWNGPMAV	121572	NA	CD8	NA	TRAV12-2	TRAJ22	TRBV9	TRBD1, TRBD2	TRBJ2-7	28103239
CVVRSYNFNKFYF	2	CVVIVYNFNKFYF	M.Tuberculosis	D009169	Yes	NA	NA	NA	PBMC	CD4	NA	TRAV8-4	TRAJ21	TRBV2	NA	TRBJ2-7	32341563
CATSGNTPLVF	2	CAVDSGNTPLVF	Yellow fever virus	D015005	Yes	YFV-17D	LLWNGPMAV	121572	NA	CD8	NA	TRAV12-2	TRAJ29	TRBV12-04	TRBD1	TRBJ2-7	28103239
CVALSGTYKYIF	2	CAVSGTYKYIF	Yellow fever virus	D015005	Yes	YFV-17D	LLWNGPMAV	121572	NA	CD8	NA	TRAV12-2	TRAJ40	TRBV4-03	TRBD2	TRBJ2-1	28103239
CAATDTGNQFYF	2	CAVADTGNQFYF	Cytomegalovirus (CMV)	D003586	Yes	pp65	NLVPMVATV	44920	PBMC	CD8	NA	TRAV22:01	TRAJ4-01	TRBV1-01	NA	TRBJ2-3:01	28636592
CALASNTGNQFYF	2	CAENSNTGNQFYF	Epstein Barr virus (EBV)	D020031	No	BMLF-1	GLCTLVAML	20788	PBMC	CD8	NA	TRAV15	TRAJ49	TRBV14	NA	TRBJ2-3	10925283
CVVNVGGGGNKLTF	1	CVVNLGGGGNKLTF	M.Tuberculosis	D009169	Yes	NA	NA	NA	PBMC	CD4	NA	TRAV12-1	TRAJ10	TRBV5-1	NA	TRBJ1-4	32341563
CVVTSNYGQNFVF	2	CAVTDNYGQNFVF	Neoantigen	C050269	Yes	HAUS3-ILN-T7A	ILNAMIAKI	NA	NA	NA	NA	TRAV29/DV5	NA	TRBV6-5	NA	NA	30418433
CAVGDRNTGFQKLVF	2	CAVRDNTGFQKLVF	Diabetes Type 1	D003922	Yes	NA	NA	NA	Pancreatic islets	CD4	NA	TRAV8-4	TRAJ8	TRBV6-1	NA	TRBJ2-5	27920090
CAPAGADKLIF	2	CAGADKLIF	Neoantigen	C050269	Yes	GNL3L-R4C	NLNCCSVPV	NA	NA	NA	NA	TRAV3	NA	TRBV6-5	NA	NA	30418433
CAVLVSNFGNEKLTF	2	CAVSPVSNFGNEKLTF	Influenza	D009980	Yes	Matrix protein (M1)	GILGFVFTL	20354	PBMC	CD8	NA	TRAV8-6:02	TRAJ4-01	TRBV5-01	NA	TRBJ2-1:01	28636592
CASQRYGGSQGNLIF	2	CAVRYGGSQGNLIF	Breast Cancer	D001943	No	NA	NA	NA	Breast	CD8	TIL	TRAV41	TRAJ42	TRBV7-03	NA	TRBJ2-4	27307436
CVVSGSTYKYIF	2	CAVSGTYKYIF	Yellow fever virus	D015005	Yes	YFV-17D	LLWNGPMAV	121572	NA	CD8	NA	TRAV12-2	TRAJ40	TRBV4-03	TRBD2	TRBJ2-1	28103239
CAVSSSGGSYIPTF	2	CAEISSGGSYIPTF	Influenza	D009980	Yes	Matrix protein (M1)	GILGFVFTL	20354	PBMC	CD8	NA	TRAV13-2:01	TRAJ6-01	TRBV9-01	NA	TRBJ2-3:01	28636592
CAARSYNTDKLIF	2	CAARANTDKLIF	Yellow fever virus	D015005	Yes	YFV-17D	LLWNGPMAV	121572	NA	CD8	NA	TRAV29/DV5	TRAJ34	TRBV15	TRBD1	TRBJ1-4	28103239
CAYRSGLTGANSKLTF	2	CAYRSTTGANSKLTF	M.Tuberculosis	D009169	Yes	NA	NA	NA	PBMC	CD4	NA	mTRDV2-2	TRAJ56	TRBV20-1	NA	TRBJ1-6	32341563

CAGPSGGTYKYIF	1	CAGPSGTYKYIF	M.Tuberculosis	D009169	Yes	NA	NA	NA	PBMC	CD4	NA	TRAV35	TRAJ40	TRBV11-3	NA	TRBJ 2-5	32341563
CAVIRTTGNQFYF	2	CAVRLTGNQFYF	M.Tuberculosis	D009169	Yes	NA	NA	NA	PBMC	CD4	NA	TRAV21	TRAJ49	TRBV29-1	NA	TRBJ 1-3	32341563
CAVQRRGFQKLVF	2	CAVRRGAQKLVF	M.Tuberculosis	D009169	Yes	NA	NA	NA	PBMC	CD4	NA	TRAV21	TRAJ54	TRBV12-3	NA	TRBJ 1-6	32341563
CAGRNSGGYQKVTF	2	CAGSGGYQKVTF	Diabetes Type 1	D003922	No	GAD65	NA	NA	PBMC	CD4	NA	NA	NA	NA	NA	TRBJ 2-1	25681349
CVVNGGGSNYKLTF	2	CAVGGGGSNYKLTF	M.Tuberculosis	D009169	Yes	NA	NA	NA	PBMC	CD4	NA	TRAV22	TRAJ53	TRBV6-1	NA	TRBJ 1-1	32341563
CAMREANTNAGKSTF	1	CAMREAYNTNAGKSTF	Diabetes Type 1	D003922	Yes	NA	NA	NA	Pancreatic islets	CD4	NA	TRDV1	TRAJ19	TRBV18	NA	TRBJ 1-3	27920090
CLVGPAAGNKLTF	1	CLVGEAAGNKLTF	Celiac disease	D002507	Yes	DQ-a-II	PQPELPYPQ	49047	Small intestine	CD4	NA	TRAV12-2	TRAJ17-1	TRBV7-2*01	TRBD2*01	TRBJ 2-7*01	24777060
CAFHGSSNTGKLIF	2	CAGAHGSSNTGKLIF	Influenza	D009980	Yes	Matrix protein (M1)	GILGFVFTL	20354	PBMC	CD8	NA	TRAV2-01	TRAJ3-01	TRBV1-01	NA	TRBJ 2-7:01	28636592
CAVRSEDSSYKLIF	2	CAVRATDSSYKLIF	Cytomegalovirus (CMV)	D003586	Yes	pp65	NLVPMVATV	44920	PBMC	CD8	NA	TRAV1-2:01	TRAJ1-01	TRBV7-01	NA	TRBJ 2-7:01	28636592
CAGDQAGTALIF	2	CAAPQAGTALIF	Melanoma	D008545	No	BAGE	AARAVFLAL	NA	PBMC	CD8	NA	TRAV8-2	TRAJ15	TRBV12	NA	TRBJ 2-1	8921424
CAFVPQGGSEKLVF	2	CAVHPQGGSEKLVF	Alzheimer's disease	D000544	Yes	NA	NA	NA	CSF	CD8	effector memory CD45RA+	TRAV13-1	TRAJ57/58	TRBV7-9	NA	TRBJ 2-2	31915375
CALRTNNNDMRF	2	CALLYNNNDMRF	Influenza	D009980	Yes	Matrix protein (M1)	GILGFVFTL	20354	PBMC	CD8	NA	TRAV19	NA	TRBV27	NA	NA	28636589
CAERMDTGRRALTF	2	CAGYMDTGRRALTF	Yellow fever virus	D015005	Yes	YFV-17D	LLWNGPMAV	121572	NA	CD8	NA	TRAV25	TRAJ5	TRBV15	TRBD2	TRBJ 2-7	28103239
CIVRDYKLSF	2	CAVRIDYKLSF	Cytomegalovirus (CMV)	D003586	No	pp65	NLVPMVATV	44920	PBMC	CD8	NA	TRAV1-4	TRAJ20	TRBV6-02	NA	TRBJ 1-1	16237109
CIRGIYGGSQGNLIF	2	CIRYGGSQGNLIF	Celiac disease	D002446	Yes	DQ2.5-glia-?2	NA	NA	PBMC	CD4	NA	TRAV12-3	TRAJ22	NA	NA	NA	33927715

CALSDRGGTASKLTF	2	CALSENRGGTASKLTF	M. tuberculosis	D009169	Yes	NA	NA	NA	PBMC	CD8	NA	NA	TRAJ44	TRBV20-1	NA	TRBJ1-305	30992377
CAASAFHQTGANNLFF	2	CAASAQTGANNLFF	M.Tuberculosis	D009169	Yes	NA	NA	NA	PBMC	CD4	NA	TRAV29/DV5	TRAJ36	TRBV5-1	NA	TRBJ2-1	32341563
CAVGETGGFKTIF	2	CAVNTGGFKTIF	Melanoma	D008545	No	Melan-A/MART-1	EAAGIGILTV	10987	Tumor Tissue	NA	NA	TRAV2	TRAJ9	NA	NA	NA	7777568
CAFIGGNTPLVF	2	CAVRGGNTPLVF	M.Tuberculosis	D009169	Yes	NA	NA	NA	PBMC	CD4	NA	TRAV8-1	TRAJ29	TRBV3-1	NA	TRBJ1-1	32341563
CAAPNLYSGGGADGLTF	2	CAAPYSGGGADGLTF	Influenza	D009980	Yes	Matrix protein (M1)	GILGFVFTL	20354	Bronchoalveolar	CD8	NA	TRAV12-2:01	TRAJ4-01	TRBV2-01	NA	TRBJ2-5:01	28636592
CAAFGGSNYKLTF	2	CALGGGSNYKLTF	Influenza	D009980	Yes	PA	SSLENFRAYV	61151	Bronchoalveolar	CD8	NA	TRAV6D-6:02	TRAJ5-01	TRBV2-01	NA	TRBJ1-4:02	28636592
CAERYNQGGKLIF	2	CAYRSYNQGGKLIF	Diabetes Type 1	D003922	Yes	NA	NA	NA	Pancreatic islets	CD4	NA	TRAV38-2/DV8	TRAJ23	TRBV7-9	NA	TRBJ2-6	27920090
CAVGRPFGNVLHC	2	CAVSPFGNVLHC	Influenza	D009980	Yes	Matrix protein (M1)	GILGFVFTL	20354	NA	NA	NA	TRAV14/DV4	NA	TRBV2	NA	NA	30418433
CAYRSENRDDKIIF	2	CAYSGNRDDKIIF	M.Tuberculosis	D009169	Yes	NA	NA	NA	PBMC	CD4	NA	mTRDV2-2	TRAJ30	TRBV19	NA	TRBJ2-1	32341563
CAASGSDGQKLLF	2	CAMRGSDGQKLLF	Neoantigen	C050269	Yes	NSDHL-A9V	KLVALGINAV	NA	NA	NA	NA	TRAV27	NA	TRBV9	NA	NA	30418433
CAENRSGGGNKLTF	2	CAENWSGGGGNKLTF	M. tuberculosis	D009169	Yes	NA	NA	NA	PBMC	CD8	NA	NA	TRAJ10	TRBV12-3	NA	TRBJ2-4	30992377
CIVRGTDSWGKLQF	2	CIVMTTDSWGKLQF	M.Tuberculosis	D009169	Yes	NA	NA	NA	PBMC	CD4	NA	TRAV26-1	TRAJ24	TRBV30	NA	TRBJ1-3	32341563
CAVRTNTGNQFYF	2	CARNTGNQFYF	Cytomegalovirus (CMV)	D003586	No	pp65	NLVPMVATV	44920	PBMC	CD8	NA	TRAV18	TRAJ49	TRBV6-03	NA	TRBJ1-4	16237109
CAMNLLQGAQKLVF	2	CAASLLQGAQKLVF	Diabetes Type 1	D003922	Yes	IGRP	VLFGLGFAI	103705	Pancreatic islets	CD8	NA	TRAV29/DV5:01	TRAJ54:01	TRBV3-1:01	TRBD1:01	TRBJ2-4:01	28300170
CAVPKGDTGRRALTF	2	CAVRGDTGRRALTF	M.Tuberculosis	D009169	Yes	NA	NA	NA	PBMC	CD4	NA	TRAV8-1	TRAJ5	TRBV12-5	NA	TRBJ1-2	32341563

CAVNNNARLMF	2	CAEDNNARLMF	Epstein Barr virus (EBV)	D020031	No	BMLF-1	GLCTLVAML	20788	Synovial Fluid	CD8	NA	TRAV15	TRAJ31	TRBV20-01	NA	TRBJ 1-2	10925283
CAANDHNNARLMF	2	CAASDNNARLMF	M.Tuberculosis	D009169	Yes	NA	NA	NA	PBMC	CD4	NA	TRAV29/DV5	TRAJ31	TRBV29-1	NA	TRBJ 1-1	32341563
CALTTGANSKLTF	2	CALIAGANSKLTF	Influenza	D009980	Yes	Matrix protein (M1)	GILGFVFTL	20354	PBMC	CD8	NA	TRAV9-2:01	TRAJ5-01	TRBV19:02	NA	TRBJ 2-3:01	28636592
CAVEYGGSQGNLIF	2	CIVYGGSQGNLIF	Human immunodeficiency virus (HIV)	D006678	No	RF10 Protein Nef	RYPLTFGWCF	56620	PBMC	CD8	NA	TRAV26-1	TRAJ42	TRBV23-01	TRBD1	TRBJ 1-5	24899498
CAVYTGGGNKLTF	2	CAEVSTGGGNKLTF	Human immunodeficiency virus (HIV)	D006678	No	RF10 Protein Nef	RYPLTFGWCF	56620	PBMC	CD8	NA	TRAV13-2	TRAJ10	TRBV4-01	TRBD1	TRBJ 1-1	24899498
CAVGTNAGKSTF	2	CAVNAGKSTF	Yellow fever virus	D015005	Yes	YFV-17D	LLWNGPMAV	121572	NA	CD8	NA	TRAV12-2	TRAJ27	TRBV6-06	TRBD1, TRBD2	TRBJ 2-7	28103239
CAVRAARLMF	2	CAVTSARLMF	Yellow fever virus	D015005	Yes	YFV-17D	LLWNGPMAV	121572	NA	CD8	NA	TRAV12-2	TRAJ31	TRBV4-03	TRBD1	TRBJ 2-7	28103239
CAYGVNRDDKIIF	2	CAGVGRDDKIIF	Breast Cancer	D001943	No	NA	NA	NA	Breast	CD8	TIL	TRAV25	TRAJ30	TRBV7-02	NA	TRBJ 1-5	27307436

TRAV1-2 negative CDR3beta sequence similarity

Query	Dist	CDR3.beta.aa	Pathology	Pathology.Mesh.ID	Single.cell	Antigen.protein	Epitope.peptide	Epitope.ID	Tissue	T.Cell.Type	T.cell.characteristics	TRAV	TRAJ	TRBV	TRBD	TRBJ	PubMed.ID
CATSRDLPGNSPLHF	2	CATSRDRGGNSPLHF	M.Tuberculosis	D009169	Yes	NA	NA	NA	PBMC	CD4	NA	TRAV9-1	TRAJ7	TRBV15	NA	TRBJ 1-6	32341563
CSARDGTSGDTGELFF	1	CSARDGTSDTGELFF	M. tuberculosis	D009169	Yes	NA	NA	NA	PBMC	CD8	NA	NA	TRAJ33	TRBV20-1	NA	TRBJ 2-2	30992377
CASSLRPSNQPQHF	2	CASSLGPGNQPQHF	M.Tuberculosis	D009169	Yes	NA	NA	NA	PBMC	CD4	NA	mTRDV2-2	TRAJ40	TRBV11-1	NA	TRBJ 1-5	32341563
CASSLTGGLGTEAFF	2	CASSLDGGGGTEAFF	M.Tuberculosis	D009169	Yes	NA	NA	NA	PBMC	CD4	NA	TRAV21	TRAJ15	TRBV5-1	NA	TRBJ 1-1	32341563
CSARQGATEAFF	2	CSARPGDTEAFF	M. tuberculosis	D009169	Yes	NA	NA	NA	PBMC	CD8	MAIT	NA	TRAJ33	TRBV20-1	NA	TRBJ 1-1	30992377

CASSQDGSTDTQYF	2	CASSAPGSTDTQYF	M. tuberculosis	D009169	Yes	NA	NA	NA	PBMC	CD8	iNKT	TRAV3	TRAJ18	TRBV25-1	NA	TRBJ2-3	30992377
CASSYSNSGSGANVLTF	2	CASSYSGGSGANVLTF	M. tuberculosis	D009169	Yes	NA	NA	NA	PBMC	CD8	NA	NA	TRAJ33	TRBV6-2	NA	TRBJ2-6	30992377
CASSELAGGQETQYF	2	CASLLAGGQETQYF	M.Tuberculosis	D009169	Yes	NA	NA	NA	PBMC	CD4	NA	TRAV26-1	TRAJ39	TRBV2	NA	TRBJ2-5	32341563
CASSPRPLYEQYF	2	CASSIRPSYEQYF	Influenza	D009980	Yes	Matrix protein (M1)	GILGFVFTL	20354	PBMC	CD8	NA	TRAV8-2:01	TRAJ41:01	TRBV19:01	TRBD2:01	TRBJ2-7:01	28300170
CASSPGLADIDTQYF	2	CASSLGLAGIDTQYF	M.Tuberculosis	D009169	Yes	NA	NA	NA	PBMC	CD4	NA	TRAV19	TRAJ53	TRBV5-1	NA	TRBJ2-3	32341563
CASSEQGNTGELFF	2	CASSAGNTGELFF	M. tuberculosis	D009169	Yes	NA	NA	NA	PBMC	CD8	NA	NA	TRAJ20	TRBV6-4	NA	TRBJ2-2	30992377
CASSRSQGNTEAFF	2	CASSPSQGVTEAFF	M.Tuberculosis	D009169	Yes	NA	NA	NA	PBMC	CD4	NA	TRAV12-3	TRAJ40	TRBV18	NA	TRBJ1-1	32341563
CASSVTGYEQFF	2	CASSHSGYEQFF	Yellow fever virus	D015005	Yes	YFV-17D	LLWNGPMAV	121572	NA	CD8	NA	TRAV12-1	TRAJ50	TRBV4-01	TRBD1, TRBD2	TRBJ2-1	28103239
CASSLTGEYNEQFF	2	CARSTGEYNEQFF	M. tuberculosis	D009169	Yes	NA	NA	NA	PBMC	CD8	NA	NA	TRAJ33	TRBV30	NA	TRBJ2-1	30992377
CASSLRHLNTEAFF	2	CASSLHMNTEAFF	M.Tuberculosis	D009169	Yes	NA	NA	NA	PBMC	CD4	NA	TRAV3	TRAJ15	TRBV7-2	NA	TRBJ1-1	32341563
CASSLDGTSGVTDTQYF	2	CASSDGTSGGTDTQYF	M. tuberculosis	D009169	Yes	NA	NA	NA	PBMC	CD8	NA	NA	TRAJ20	TRBV6-4	TRBD2	TRBJ2-3	30992377
CASRNGGYEQYF	2	CASGDGGYEQYF	Influenza	D009980	Yes	NP	ASNENMETM	4602	Bronchoalveolar	CD8	NA	TRAV14D-3/DV8:08	TRAJ2-01	TRBV1-01	NA	TRBJ2-7:01	28636592
CASSPLGTGNNEQYF	2	CASSFLGTGLNEQYF	Hepatitis C virus (HCV)	D006526	Yes	HCV-KLV(PE)	KLVALGINAV	32208	NA	NA	NA	TRAV19	NA	TRBV28	NA	NA	30418433
CASSEASGGADTQYF	2	CASSDSGGADTQYF	M. tuberculosis	D009169	Yes	NA	NA	NA	PBMC	CD8	NA	NA	TRAJ9	TRBV6-4	NA	TRBJ2-3	30992377
CASSESGGGETQYF	2	CASSDASGGGETQYF	M. tuberculosis	D009169	Yes	NA	NA	NA	PBMC	CD8	NA	NA	TRAJ20	TRBV6-1	NA	TRBJ2-5	30992377
CASSLGSRNEQFF	2	CASSILGSYNEQFF	Tumor associated antigen (TAA)	D018290	Yes	EphA2	IMNDMPIYM	NA	PBMC	CD8	NA	TRAV19	TRAJ23	TRBV19	NA	TRBJ2-1	33562731

CASSLGGLDGYTF	2	CASSDGGKDGYTF	Hepatitis C virus (HCV)	D006526	Yes	HCV-KLV	KLVALGINAV	32208	NA	NA	NA	NA	NA	TRBV25-1	NA	NA	30418433
CASSQVSGNTEAFF	2	CASSLVSENTEAFF	M.Tuberculosis	D009169	Yes	NA	NA	NA	PBMC	CD4	NA	TRAV8-6	TRAJ36	TRBV5-1	NA	TRBJ1-1	32341563
CASSVRLSTDTQYF	2	CASSARSTDTQYF	Influenza	D009980	Yes	Matrix protein (M1)	GILGFVFTL	20354	PBMC	CD8	NA	TRAV6-01	TRAJ4-01	TRBV19:02	NA	TRBJ2-3:01	28636592
CASSDGQGREKLFF	2	CASSAGQGGEKLFF	M.Tuberculosis	D009169	Yes	NA	NA	NA	PBMC	CD4	NA	TRAV10	TRAJ18	TRBV25-1	NA	TRBJ1-4	32341563
CASSSGTGDTGELFF	2	CASSAGTGHTGELFF	M. tuberculosis	D009169	Yes	NA	NA	NA	PBMC	CD8	NA	NA	TRAJ12	TRBV6-1	NA	TRBJ2-2	30992377
CASKLGQGGYEQYF	2	CASSFGQGGYEQYF	Influenza	D009980	Yes	Matrix protein (M1)	GILGFVFTL	20354	PBMC	CD8	NA	TRAV8-2	NA	TRBV13	NA	NA	28636589
CASSFGDRGREQYF	2	CASSQDRGREQYF	M.Tuberculosis	D009169	Yes	NA	NA	NA	PBMC	CD4	NA	TRAV17	TRAJ8	TRBV4-3	NA	TRBJ2-7	32341563
CASSQAAGGNTDTQYF	2	CASSEAAGTGNTDTQYF	M. tuberculosis	D009169	Yes	NA	NA	NA	PBMC	CD8	NA	NA	TRAJ33	TRBV6-1	NA	TRBJ2-3	30992377
CASSSRQGHTGELFF	2	CASSDRGHTGELFF	M. tuberculosis	D009169	Yes	NA	NA	NA	PBMC	CD8	NA	NA	TRAJ49	TRBV6-4	NA	TRBJ2-2	30992377
CASSEMGTGELFF	2	CASSAQGTGELFF	M.Tuberculosis	D009169	Yes	NA	NA	NA	PBMC	CD4	NA	TRAV21	TRAJ56	TRBV3-1	NA	TRBJ2-2	32341563
CASSDGSGGYGYTF	2	CASSDGTGPYGYTF	Diabetes Type 1	D003922	Yes	NA	NA	NA	Pancreatic islets	CD4	NA	TRAV22	TRAJ28	TRBV6-1	NA	TRBJ1-2	27920090
CASSLGLAGTTDTQYF	2	CASSGLAGGTDTQYF	M. tuberculosis	D009169	Yes	NA	NA	NA	PBMC	CD8	MAIT	NA	TRAJ33	TRBV6-4	TRBD2	TRBJ2-3	30992377
CASSQHDGYTQYF	2	CASSQVDGYEQYF	M.Tuberculosis	D009169	Yes	NA	NA	NA	PBMC	CD4	NA	TRAV9-1	TRAJ44	TRBV3-1	NA	TRBJ2-7	32341563
CASSTGQGNTIYF	2	CASSAGGGNTIYF	M.Tuberculosis	D009169	Yes	NA	NA	NA	PBMC	CD4	NA	TRAV13-1	TRAJ45	TRBV5-1	NA	TRBJ1-3	32341563
CASSDSSGGANEQFF	2	CASSDASGGAYNEQFF	M. tuberculosis	D009169	Yes	NA	NA	NA	PBMC	CD8	NA	NA	TRAJ33	TRBV6-4	NA	TRBJ2-1	30992377
CASSDPERTEAFF	2	CASSDAENTEAFF	Cytomegalovirus (CMV)	D003586	Yes	pp65	NLVPMVATV	44920	PBMC	CD8	NA	TRAV1-2:01	TRAJ3-01	TRBV6-01	NA	TRBJ1-1:01	28636592

CASSPGTGLSYEQYF	2	CASSEGTGGSYEQYF	M. tuberculosis	D009169	Yes	NA	NA	NA	PBMC	CD8	MAIT	NA	TRAJ33	TRBV6-1	TRBD1	TRBJ2-7	30992377
CASSAPLNNEQFF	2	CASSHPLGNEQFF	M.Tuberculosis	D009169	Yes	NA	NA	NA	PBMC	CD4	NA	TRAV8-1	TRAJ31	TRBV4-1	NA	TRBJ2-1	32341563
CASSLQGQGADEQFF	2	CASSLEGQGASEQFF	Celiac disease	D002503	Yes	DQ2-a-I	PFPQPELPY	47539	Small intestine	CD4	NA	TRAV9-1	TRAJ33-1	TRBV5-5*01	TRBD2*01	TRBJ2-1	24777060
CASSLNGGYNEQFF	2	CASSALAGGYNEQFF	Neoantigen	C050269	Yes	KIF20B-YTS-S6L	YTSEILSPI	NA	NA	NA	NA	NA	NA	TRBV2	NA	NA	30418433
CASSSMLSETQYF	2	CASSQLSETQYF	M.Tuberculosis	D009169	Yes	NA	NA	NA	PBMC	CD4	NA	TRAV12-1	TRAJ45	TRBV3-1	NA	TRBJ2-5	32341563
CSARGLGNTEAFF	2	CSARDLGNLEAFF	M.Tuberculosis	D009169	Yes	NA	NA	NA	PBMC	CD4	NA	TRAV9-1	TRAJ23	TRBV20-1	NA	TRBJ1-1	32341563
CASSHRNTGELFF	2	CASSAGNTGELFF	M. tuberculosis	D009169	Yes	NA	NA	NA	PBMC	CD8	NA	NA	TRAJ20	TRBV6-4	NA	TRBJ2-2	30992377
CASSLELLVEQFF	2	CASSLTLLEQFF	M.Tuberculosis	D009169	Yes	NA	NA	NA	PBMC	CD4	NA	TRAV26-1	TRAJ54	TRBV13	NA	TRBJ2-1	32341563
CSASLSDYGYTF	2	CSASRTDYGYTF	M.Tuberculosis	D009169	Yes	NA	NA	NA	PBMC	CD4	NA	mTRAV14D-1	TRAJ8	TRBV20-1	NA	TRBJ1-2	32341563
CATSRDKGVNQPQHF	2	CATSRDPGPNQPQHF	M.Tuberculosis	D009169	Yes	NA	NA	NA	PBMC	CD4	NA	TRAV26-1	TRAJ24	TRBV15	NA	TRBJ1-5	32341563
CASSLAMGLYEQYF	2	CASKSLAGGLYEQYF	M.Tuberculosis	D009169	Yes	NA	NA	NA	PBMC	CD4	NA	TRAV17	TRAJ57	TRBV2	NA	TRBJ2-7	32341563
CASSLDPSDSGANVLTF	2	CASSLDGDSGANVLTF	M. tuberculosis	D009169	Yes	NA	NA	NA	PBMC	CD8	NA	NA	TRAJ33	TRBV6-2	NA	TRBJ2-6	30992377
CASSEDPTDTQYF	2	CASSEGITDTQYF	M.Tuberculosis	D009169	Yes	NA	NA	NA	PBMC	CD4	NA	TRAV13-1	TRAJ33	TRBV7-2	NA	TRBJ2-3	32341563
CASSPQHPQETQYF	2	CASSPWQHQETQYF	M.Tuberculosis	D009169	Yes	NA	NA	NA	PBMC	CD4	NA	TRAV21	TRAJ13	TRBV18	NA	TRBJ2-5	32341563
CASSFGSDSNQPQHF	2	CASSFADSNQPQHF	M.Tuberculosis	D009169	Yes	NA	NA	NA	PBMC	CD4	NA	TRAV25	TRAJ22	TRBV11-2	NA	TRBJ1-5	32341563
CASSQTGNQPQHF	2	CASQGNQPQHF	M.Tuberculosis	D009169	Yes	NA	NA	NA	PBMC	CD4	NA	TRAV26-1	TRAJ5	TRBV28	NA	TRBJ1-5	32341563
CASSRGGGSEKLFF	2	CASSGRGGGEKLFF	M. tuberculosis	D009169	Yes	NA	NA	NA	PBMC	CD8	iNKT	NA	TRAJ18	TRBV25-1	NA	TRBJ1-4	30992377

CASSFGGGRSSYNEQFF	2	CASSDSGGRSSYNEQFF	M.Tuberculosis	D009169	Yes	NA	NA	NA	PBMC	CD4	NA	TRAV10	TRAJ18	TRBV25-1	NA	TRBJ2-1	32341563
CASSGTGRVDTQYF	2	CASSGTGQDTQYF	mCMV	D018146	Yes	m139	TVYGFCLL	67227	Spleen	CD8	NA	TRAV7-2:02	TRAJ5-01	TRBV4-01	NA	TRBJ2-5:01	28636592
CASSLGATGRASGNTIYF	2	CASSLGHTGASGNTIYF	M.Tuberculosis	D009169	Yes	NA	NA	NA	PBMC	CD4	NA	TRAV5	TRAJ34	TRBV5-1	NA	TRBJ1-3	32341563
CASSESSTTNEKLFF	2	CASSESETGNEKLFF	M.Tuberculosis	D009169	Yes	NA	NA	NA	PBMC	CD4	NA	TRAV10	TRAJ18	TRBV25-1	NA	TRBJ1-4	32341563
CASSLVASGGANEQFF	2	CASSGASGGANEQFF	M. tuberculosis	D009169	Yes	NA	NA	NA	PBMC	CD8	NA	NA	TRAJ33	TRBV6-1	NA	TRBJ2-1	30992377
CASSYDRPNTEAFF	2	CASSDRGNTEAFF	M.Tuberculosis	D009169	Yes	NA	NA	NA	PBMC	CD4	NA	TRAV8-4	TRAJ40	TRBV6-1	NA	TRBJ1-1	32341563
CASSLLGTETQYF	2	CASSLAGTDTQYF	M.Tuberculosis	D009169	Yes	NA	NA	NA	PBMC	CD4	NA	TRAV12-2	TRAJ54	TRBV5-6	NA	TRBJ2-3	32341563
CASSFGGGLGNTEAFF	2	CASSSGGGLNTEAFF	M.Tuberculosis	D009169	Yes	NA	NA	NA	PBMC	CD4	NA	TRAV19	TRAJ39	TRBV7-2	NA	TRBJ1-1	32341563
CASSQDGTSGYNEQFF	2	CASSDGTSGNEQFF	M. tuberculosis	D009169	Yes	NA	NA	NA	PBMC	CD8	MAIT	NA	TRAJ33	TRBV6-4	NA	TRBJ2-1	30992377
CASSLGQLNQPQHF	2	CASSEGLNQPQHF	M.Tuberculosis	D009169	Yes	NA	NA	NA	PBMC	CD4	NA	mTRAV14D-1	TRAJ15	TRBV6-1	NA	TRBJ1-5	32341563
