# Supplementary material for: CD4+ mucosal-associated invariant T cells express highly diverse T cell receptors
Source: J Immunol. 2025 Nov 9;214(12):3260–72. doi: 10.1093/jimmun/vkaf260 (PMC12726071; doi:10.1093/jimmun/vkaf260)
Supplement: vkaf260_Supplementary_Data [file vkaf260_supplementary_data.zip › vkaf260_Supplementary_Data/JI_Supplementary_Table1.pdf]

Supplementary Table 1: Reagents and Resources

REAGENT or RESOURCE	SOURCE	IDENTIFIER
**Antibodies (anti-human)**		
Fc Receptor Binding Inhibitor Polyclonal Antibody	eBioscience	Cat#14-9161-73; RRID:AB_468582
Zombie red Fixable viability kit	BioLegend	Cat# 423110
**Sorting fluorophore antibodies**		
APC CD161 (clone: DX12)	BD Pharmingen	Cat# 550968; RRID:AB_398482
PE MR1 tetramer	PE	
Alexa Fluor 700 CD3 (clone: UCHT1)	BD Biosciences	Cat# 557943; RRID:AB_396952
BV480 CD14 (clone: M5E2)	BD Pharmingen	Cat# 746304; RRID:AB_2743629
PE-Cy5 CD19 (clone: HIB19)	Biolegend	Cat# 302210; RRID: AB_314240
FITC CD16 (clone: B73.1)	Biolegend	Cat# 360716; RRID: AB_2563071
PE-Cy7 CD56 (clone: CMSSB)	Fisher	Cat# 25-0567-42; RRID:AB_11041529
BV650 TCRgd (clone: B1.1)	BD Pharmingen	Cat# 564156; RRID:AB_2738628
BV510 TCRVa24 (iNKT) (clone: 6B11)	Biolegend	Cat# 342918; RRID: AB_2564006
**Hashtag antibodies**		
**Antibody (Sequence) (Clone)**		
TotalSeq-C0251 MB_Exvivo (GTCAACTCTTTAGCG) (LNH-94;2M2)	BioLegend	Cat# 394661; RRID:AB_2801031
TotalSeq-C0252 MB_Rest (TGATGGCCTATTGGG) (LNH-94;2M2)	BioLegend	Cat# 394663; RRID:AB_2801032
TotalSeq-C0253 MB_Mtblysate (TTCCGCCTCTCTTTG) (LNH-94;2M2)	BioLegend	Cat# 394665; RRID:AB_2801033
TotalSeq-C0254 HD_073409_Exvivo (AGTAAGTTCAGCGTA) (LNH-94;2M2)	BioLegend	Cat# 394667; RRID:AB_2801034
TotalSeq-C0255 HD_073409_Rest (AAGTATCGTTTCGCA) (LNH-94;2M2)	BioLegend	Cat# 394669; RRID:AB_2801035
TotalSeq-C0256 HD_073409_Mtblysate (GGTTGCCAGATGTCA) (LNH-94;2M2)	BioLegend	Cat# 394671; RRID:AB_2801036
**TotalseqC antibodies**		
TotalSeq-C0072 CD4 (TGTTCCCGCTCAACT) (RPA-T4)	BioLegend	Cat# 300567; RRID:AB_2800725
TotalSeq-C0390 CD127 (GTGTGTTGTCCTATG) (A019D5)	BioLegend	Cat# 351356; RRID:AB_2800937
TotalSeq-C0081 CD14 (TCTCAGACCTCCGTA) (M5E2)	BioLegend	Cat# 301859; RRID:AB_2800736
TotalSeq-C0396 CD26 (GGTGGCTAGATAATG) (BA5b)	BioLegend	Cat# 302722; RRID:AB_2810435
TotalSeq1-C0084 CD 56 (NCAM) (TTCGCCGCATTGAGT) (QA17A16)	BioLegend	Cat# 392425; RRID:AB_2801024
TotalSeq1-C0050 CD19 (CTGGGCAATTACTCG) (HIB19)	BioLegend	Cat# 302265; RRID:AB_2800741
TotalSeq-C0147 CD62L (GTCCCTGCAACTTGA) (DREG-56)	BioLegend	Cat# 304851; RRID:AB_2800770
TotalSeq-C0155 CD107a (LAMP-1) (CAGCCCACTGCAATA) (H4A3)	BioLegend	Cat# 328649; RRID:AB_2800854
TotalSeq-C0149 CD161 (GTACGCAGTCCTTCT) (HP-3G10)	BioLegend	Cat# 339947; RRID:AB_2810532
TotalSeq-C0158 CD134 (OX40) (AACCCACCGTTGTTA) (Ber-ACT35 (ACT35)	BioLegend	Cat# 350035; RRID:AB_2800932
TotalSeq-C0101 CD335 (NKg46) (ACAATTTGAACAGCG) (9 E2)	BioLegend	Cat# 331941; RRID:AB_2800874
TotalSeq-C0171 CD278 (ICOS) (CGCGCACCCATTAAA) (C398.4A)	BioLegend	Cat# 313553; RRID:AB_2800874
TotalSeq-C0034 CD3 (CTCATTGTAACTCCT) (UCHT1)	BioLegend	Cat# 300479; RRID:AB_2800823
TotalSeq-C0146 CD69 (GTCTCTTGGCTTAAA) (FN50)	BioLegend	Cat# 310951; RRID:AB_2800810
TotalSeq-C0046 CD8 (GCGCAACTTGATGAT) (SK1)	BioLegend	Cat# 344753; RRID:AB_2800922
TotalSeq-C0159 HLA-DR (AATAGCGAGCAAGTA) (L243)	BioLegend	Cat# 307663; RRID:AB_2800795
TotalSeq-C0153 KLRG-1 (MAFA) (CTTATTTCCTGCCCT) (SA231A2)	BioLegend	Cat# 367737; RRID:AB_2904401
TotalSeq-C0063 CD45RA (TCAATCCTTCCGCTT) (HI100)	BioLegend	Cat# 304163; RRID:AB_2800764
TotalSeq-C0080 CD8a (GCTGCGCTTTCCATT) (RPA-T8)	BioLegend	Cat# 301071; RRID:AB_2800730
TotalSeq-C0007 CD274 (B7-H1, PD-L1) (GTTGTCCGACAATAC) (29E.2A3)	BioLegend	Cat# 329751; RRID:AB_2800860
TotalSeq-C0032 CD154 (GCTAGATAGATGCAA) (24-31)	BioLegend	Cat# 310849; RRID:AB_2800808
TotalSeq-C0053 CD11c (TACGCCTATAACTTG) (S-HCL-3)	BioLegend	Cat# 371521; RRID:AB_2801018
TotalSeq-C0083 CD16 (AAGTTCACTCTTTGC) (3G8)	BioLegend	Cat# 302065; RRID:AB_2800738
TotalSeq-C0867 CD94 (CTTTCCGGTCCTACA) (DX22)	BioLegend	Cat# 305523; RRID:AB_2814143
TotalSeq-C0420 CD158 (KIR2DL1/S1/S3/S5) (TATCAACCAACGCTT) (HP-MA4)	BioLegend	Cat# 339517; RRID:AB_2814252
TotalSeq-C0592 CD158b (KIR2DL2/L3, NKAT2 (GACCCGTAGTTTGAT) (DX27)	BioLegend	Cat# 312619; RRID:AB_2819944
TotalSeq-C0156 CD96 (Fas) (CCAGCTCATTAGAGC) (DX2)	BioLegend	Cat# 305651; RRID:AB_2800787

TotalSeq-C0047 CD56 (NCAM) (TCCTTTCCTGATAGG) (5.1H11)	BioLegend	Cat# 362559; RRID:AB_2801002
TotalSeq-C0151 CD152 (CTLA-4) (ATGGTTCACGTAATC) (BNI3)	BioLegend	Cat# 369621; RRID:AB_2801015
TotalSeq-C0165 CD314 (NK2D) (CGTGTTTGTTCCTCA) (1D11)	BioLegend	Cat# 320837; RRID:AB_2800844
TotalSeq-C0145 CD103 (Integrin aE) (GACCTCATTGTGAAT) (Ber-ACT8)	BioLegend	Cat# 350233; RRID:AB_2800933
TotalSeq-C0161 CD11b (GACAAGTGATCTGCA) (ICRF44)	BioLegend	Cat# 301359; RRID:AB_2800732
TotalSeq-C0168 CD57 (AACTCCCTATGGAGG) (QA17A04)	BioLegend	Cat# 393321; RRID:AB_2801030
TotalSeq-C0154 CD27 (GCACTCCTGCATGTA) (O323)	BioLegend	Cat# 302853; RRID:AB_2800747
TotalSeq-C0599 CD158e1 (GGACGCTTTCCTTGA) (DX9)	BioLegend	Cat# 312725; RRID:AB_2814161
TotalSeq-C0087 CD45RO (CTCCGAATCATGTTG) (UCHL1)	BioLegend	Cat# 304259; RRID:AB_2800766
TotalSeq-C0391 CD45 (TTTGTCCTGTACGCC) (HI30)	BioLegend	Cat# 304068; RRID:AB_2800762
TotalSeq-C0085 CD25 (TGCAATTACCCGGAT) (BC96)	BioLegend	Cat# 302649; RRID:AB_2800745
**Fc Block**		
Human Trustain FcX	BioLegend	Cat#422301; RRID: AB_2818986
**Chemicals, peptides, and recombinant proteins**		
Cytiva Ficoll-Paque™ PREMIUM	Cytiva	Cat#45001751
Ficoll Paque Plus	GE Healthcare	Cat#17144002
Fetal Bovine Serum (FBS)	Gibco	Cat#10437028
Bambanker Serum-free cell freezing media	Lymphotec Inc.	Cat#9582225
Brefeldin A solution (1000x)	Biolegend	Cat#420601
Recombinant human IL2	PeproTech	Cat#200-02
Penicillin/Streptomycin	Gibco	Cat#15-140-122
RPMI 1640	Gibco	Cat#21870092
L-glutamine	Gibco	Cat#25030149
Fetal Bovine Serum	Gibco	Cat#26140079
HEPES	Gibco	Cat#15630080
Sodium pyruvate	Gibco	Cat#11360070
MEM Nonessential amino acids	Gibco	Cat#11140050
Flow Cytometry Staining Buffer	eBioscience	Cat#00-4222-26
Fixation/Permeabilization concentrate	eBioscience	Cat#00-5123-43
Permeabilization Buffer	eBioscience	Cat#00-8333-56
2-mercaptoethanol	Sigma	Cat#M6250-250ML
**Biological samples**		
Healthy human PBMCs	NYBC, SBU	N/A
Healthy human TB contact/control PBMCs	GHESKIO Centers	N/A
**Software and algorithms**		
Cellranger	v3.0.2	https://github.com/10XGenomics/cellranger
Seurat	v2.3.4	https://satijalab.org/seurat/articles/install.html
R	V4.3.1	https://www.r-project.org/
FCS express v7	DeNovo Software	https://denovosoftware.com/
