# Supplementary material for: CD4+ mucosal-associated invariant T cells express highly diverse T cell receptors
Source: J Immunol. 2025 Nov 9;214(12):3260–72. doi: 10.1093/jimmun/vkaf260 (PMC12726071; doi:10.1093/jimmun/vkaf260)
Supplement: vkaf260_Supplementary_Data [file vkaf260_supplementary_data.zip › vkaf260_Supplementary_Data/Supplemental_Data_09-05-2025.docx]

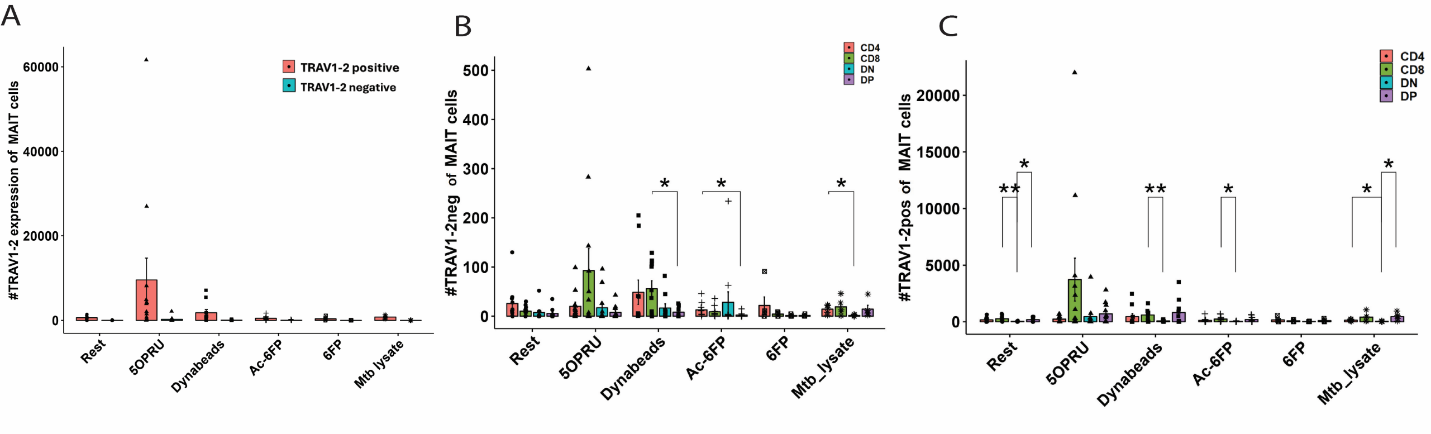


**Supplemental Figure 1: Absolute normalized counts of MAIT cells with TRAV1-2^+^ and negative TCRs. A.** MAIT cell absolute numbers stratified by TRAV1-2 staining after 7 days of incubation in vitro with various stimuli. **B.** TRAV1-2^+^ and **C**. TRAV1-2 negative MAIT cell subset absolute normalized counts. Statistical comparisons made by unpaired t-test. *p<0.05 **p<0.005


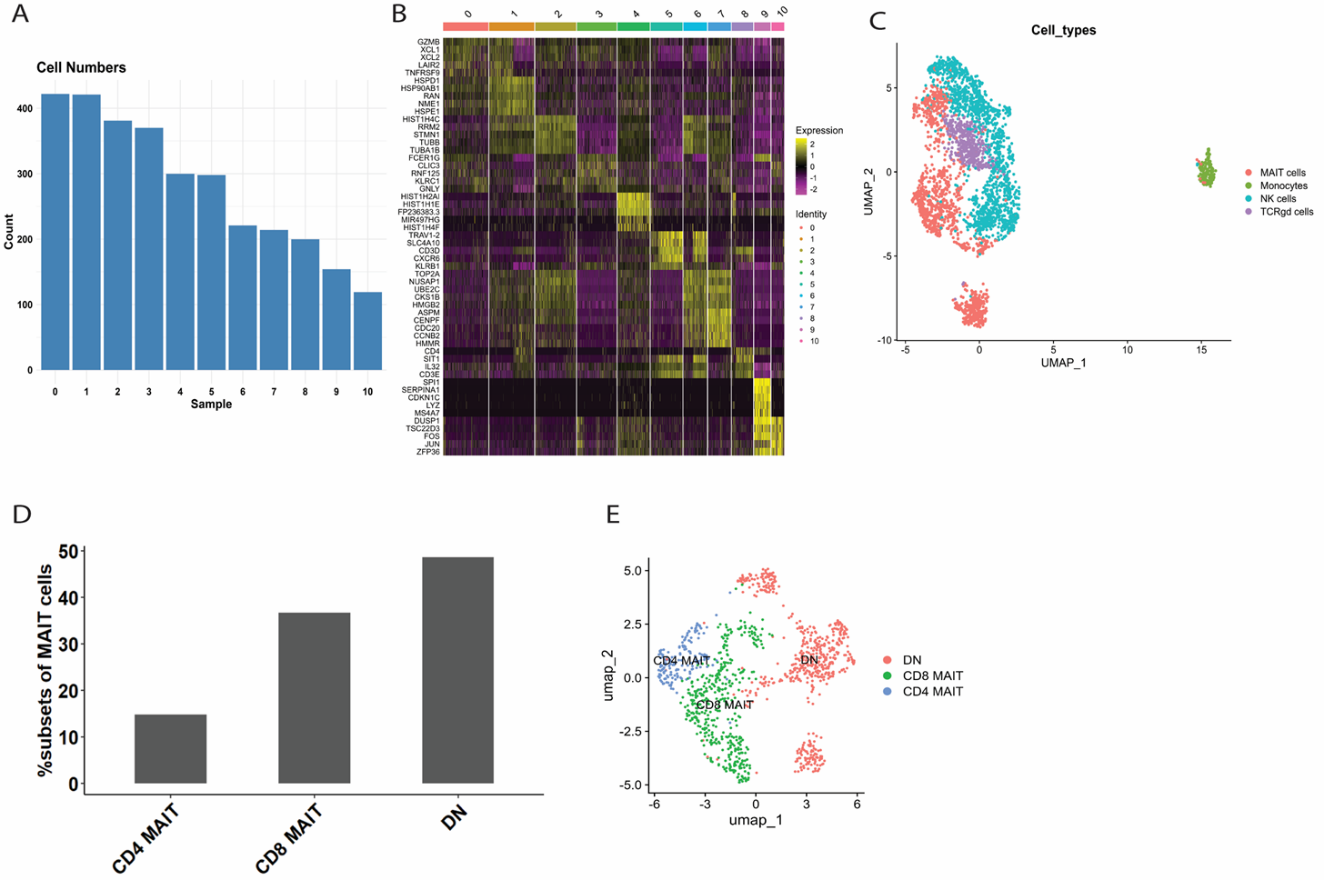


**Supplemental Figure 2: CITE-Seq analysis and visualization.** Cell count and visualization of all sequenced cells. **A**. Absolute cell count in each cluster (C0-10). **B**. Heat map displaying the top differentially expressed genes in Seurat clusters (C0-10). **C**. UMAP visualization of four cell populations found in CITE-Seq. **D**. The frequency of MAIT cell subsets in MAIT cell cluster. **E**. UMAP visualization of MAIT cell subsets.


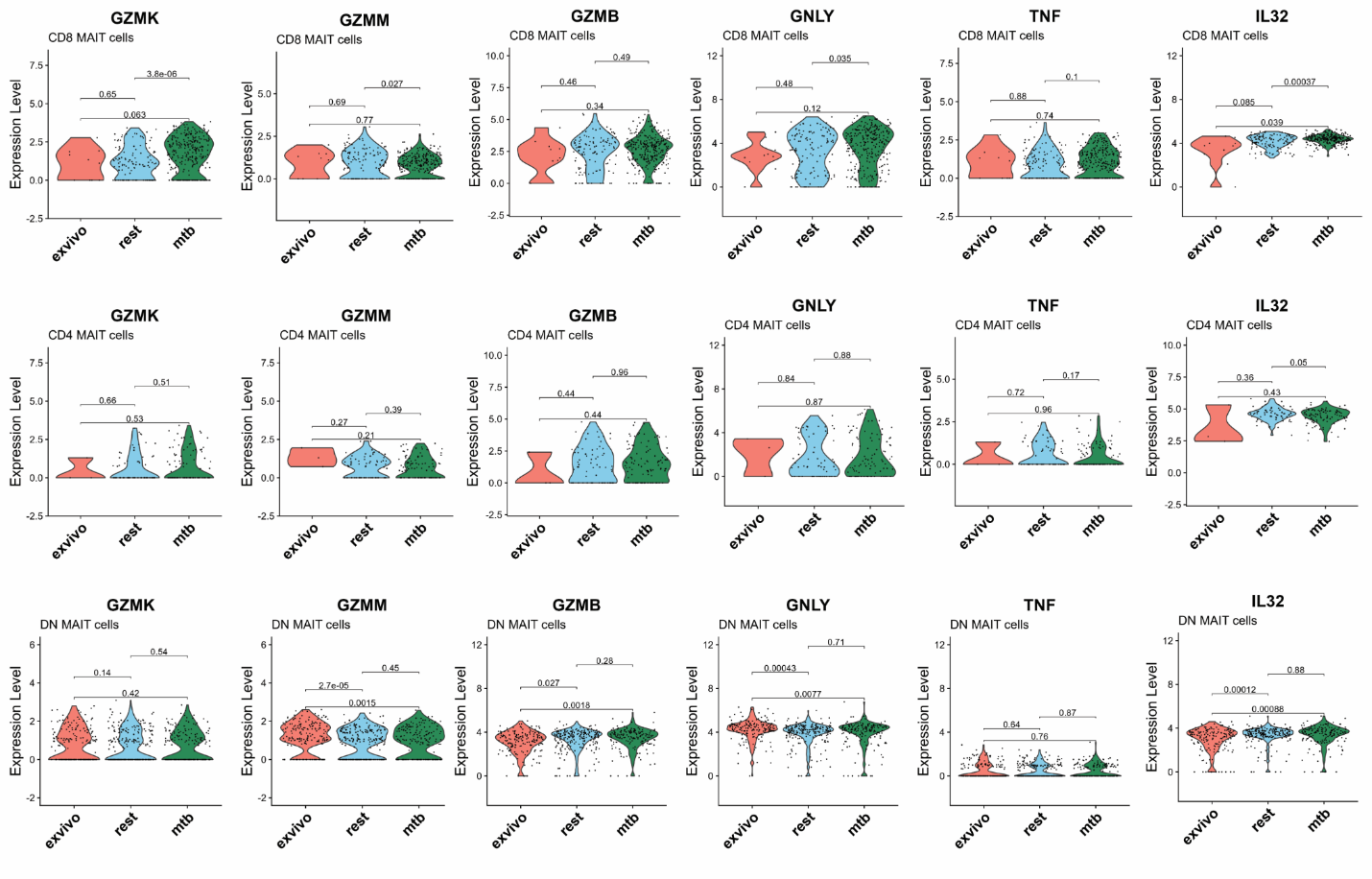


**Supplemental Figure 3: Select gene expression analysis after *Mtb* lysate induction.** Violin plots displaying the expression levels of of select genes ex vivo or after co-incubation for 7 days with IL2 + *Mtb* lysates relative to IL2 alone (rest). Statistical comparisons made by unpaired Wilcox test with reported adjusted p-values and significance level of p<0.05.


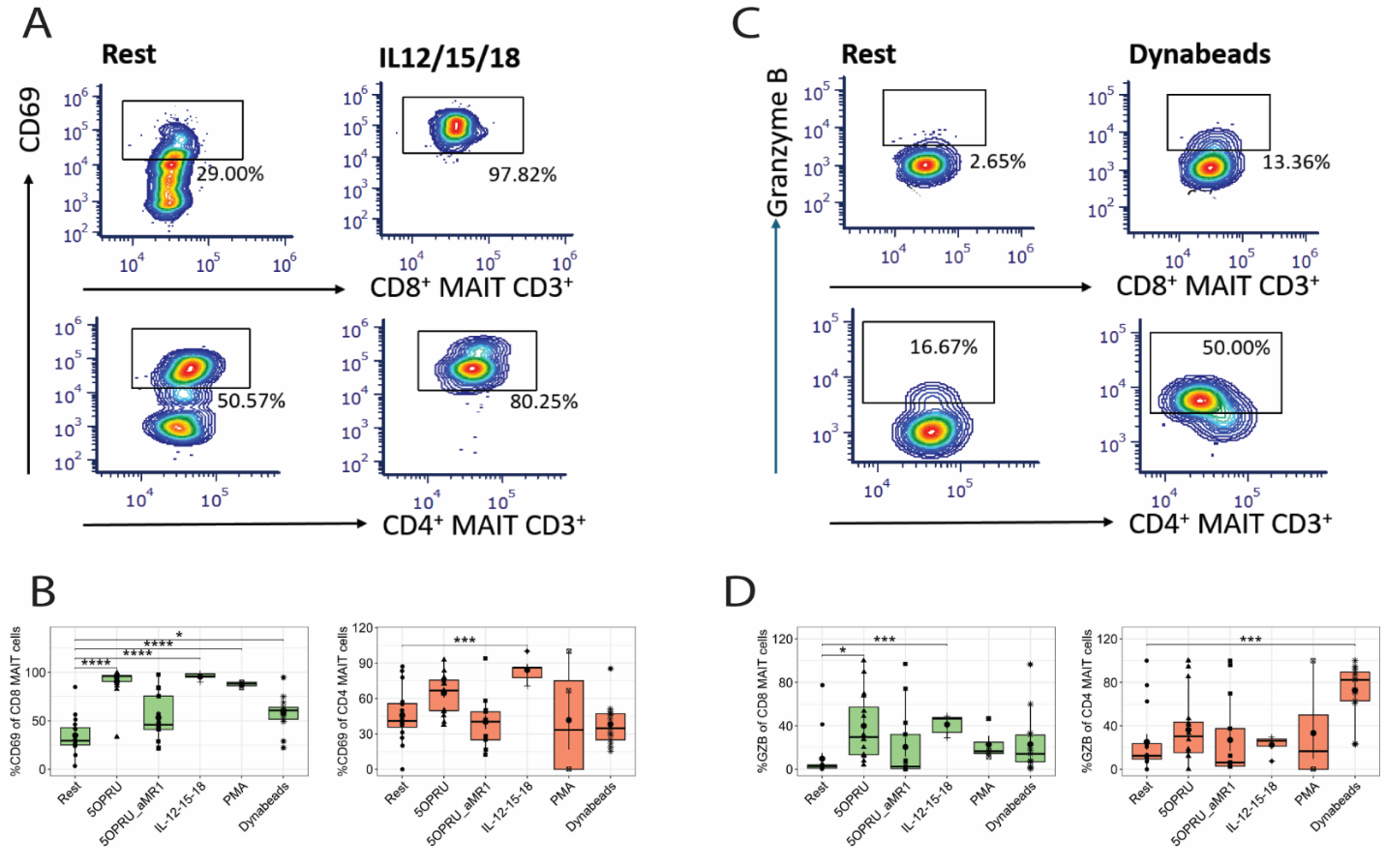


**Supplemental Figure 4: CD8^+^ and CD4^+^ MAIT cell activation and granzyme production after TCR-dependent and independent stimuli.** Representative flow contour plots demonstrating the gating strategy for CD8^+^ and CD4^+^ MAIT cells for **A.** CD69 staining with or without IL12/15/18 stimulation. **B.** Cumulative bar plots displaying the percent expression of CD69 across stimulation conditions. **C.** Representative flow contour plots demonstrating intracellular Granzyme B staining with or without anti-CD3/CD28 Dynabeads stimulation. **D.** Cumulative bar plots displaying the percent expression of Granzyme B across stimulation conditions. Statistical comparisons made by unpaired t-test. *p<0.05, ***p<0.001, ****p<0.0001


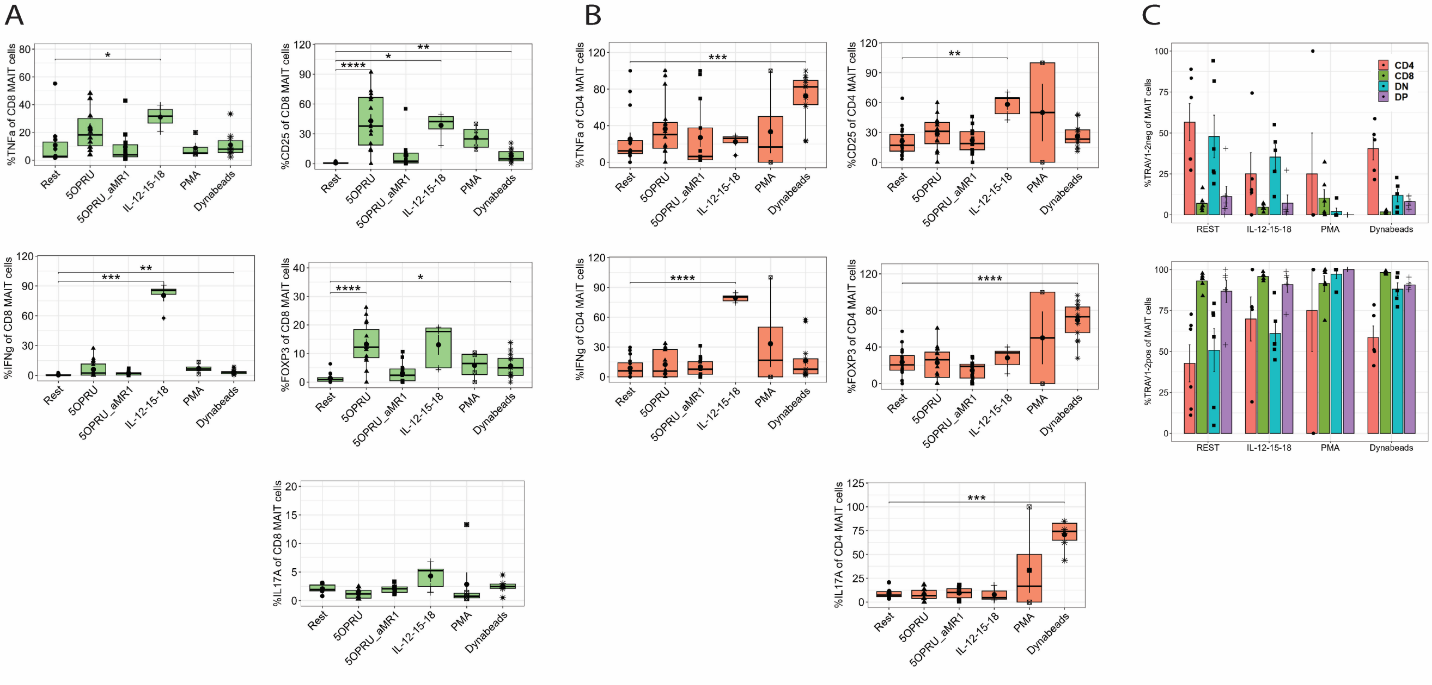


**Supplemental Figure 5: CD8^+^ and CD4^+^ MAIT cell effector function and expansion after TCR-dependent and independent stimulation.** Bar plots displaying the percent expression of CD25, IFNg, TNFα, IL17A and FOXP3 in **A.** CD8^+^ MAIT cells and **B.** CD4^+^ MAIT cells after 16 hours incubation with various stimulation conditions. **C.** TRAV1-2^+/-^ MAIT cell expansion with various stimulation conditions for 7 days. Statistical comparisons made by Wilcoxon test. *p<0.05, **p<0.005, ***p<0.001, ****p<0.0001


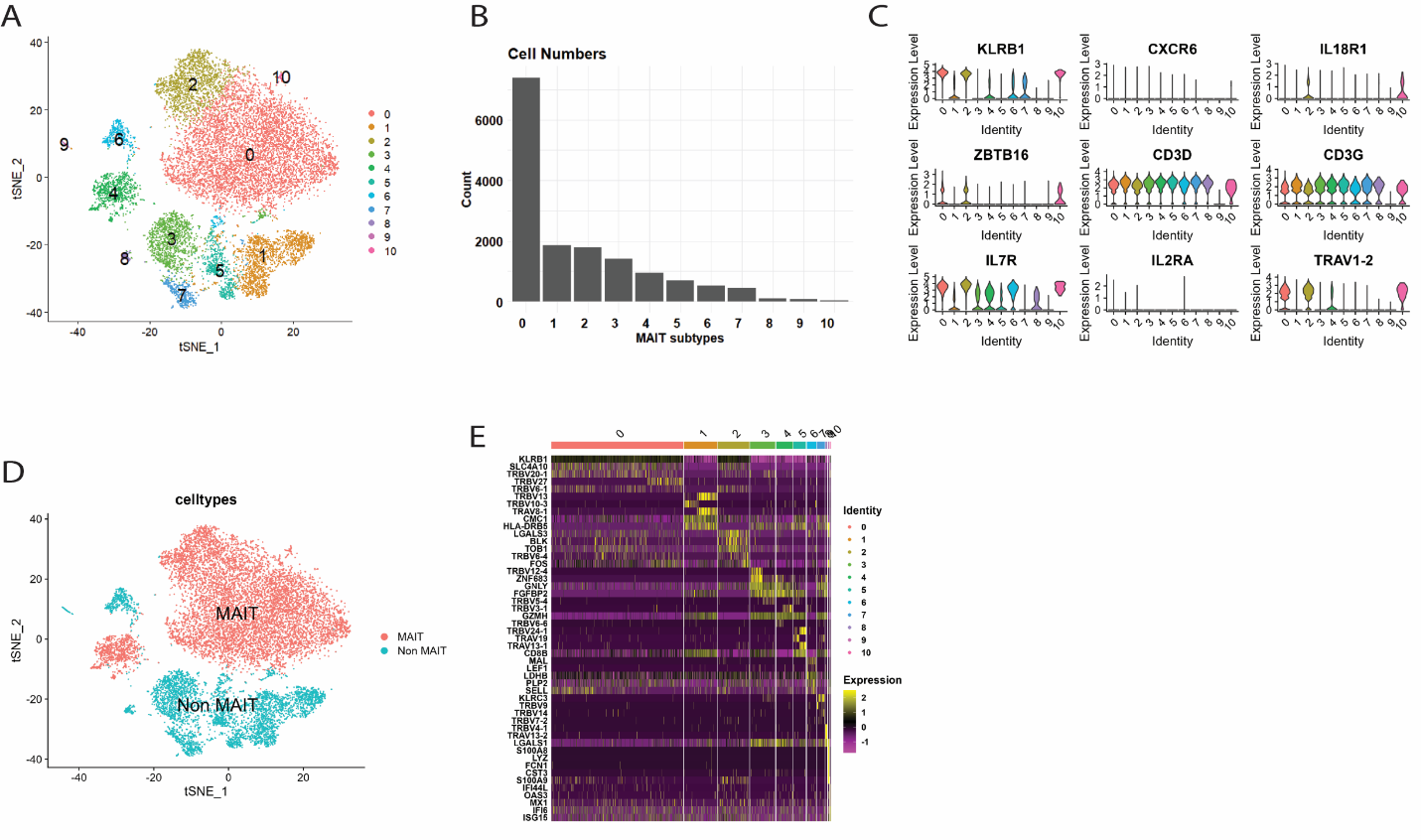


**Supplemental Figure 6:** **Visualization and gene expression profiling of cells from *Garner* et al., 2023**. **A.** TSNE visualization of all sequenced cells in experiment 1. **B.** Absolute cell count in each cluster. **C.** Gene expression of CD3 and MAIT cell-specific genes across different clusters. **D.** TSNE visualization to MAIT and Non-MAIT cells identified in *Garner* et al (2023) experiment 1 data. **E.** Heat map displaying the differential gene analysis across between clusters.


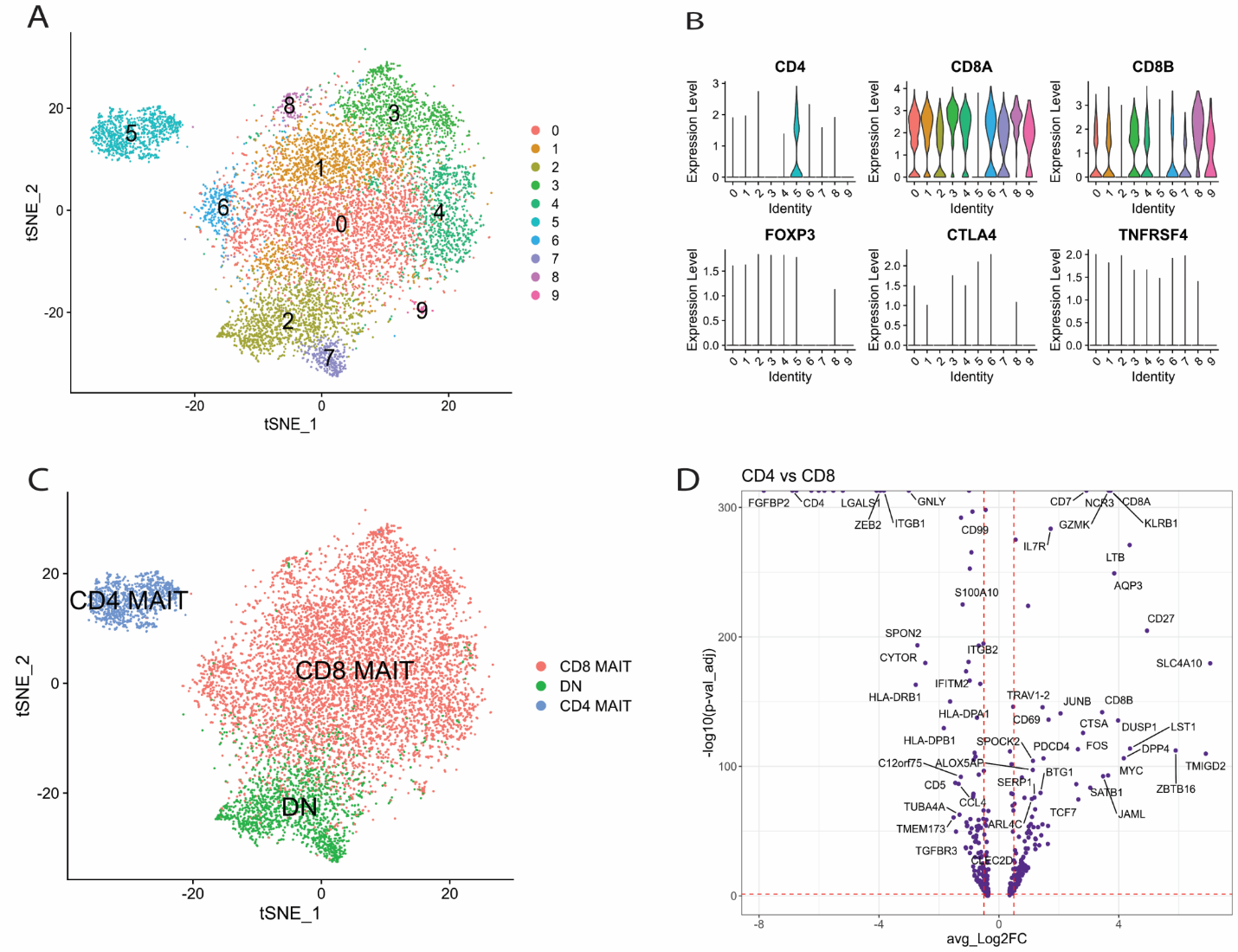


**Supplemental Figure 7: Gene expression analysis of identified MAIT cells (*Garner* et al 2023)**. **A** TSNE visualization of MAIT cell sub-clusters. **B.** CD8 and CD4 co-receptor gene expression in MAIT cell subclusters. **C.** TSNE visualization of MAIT cell subsets **D.** Volcano plot displaying differential gene expression between CD4^+^ (-log fold change) and CD8^+^ (+log fold change) MAIT cells.


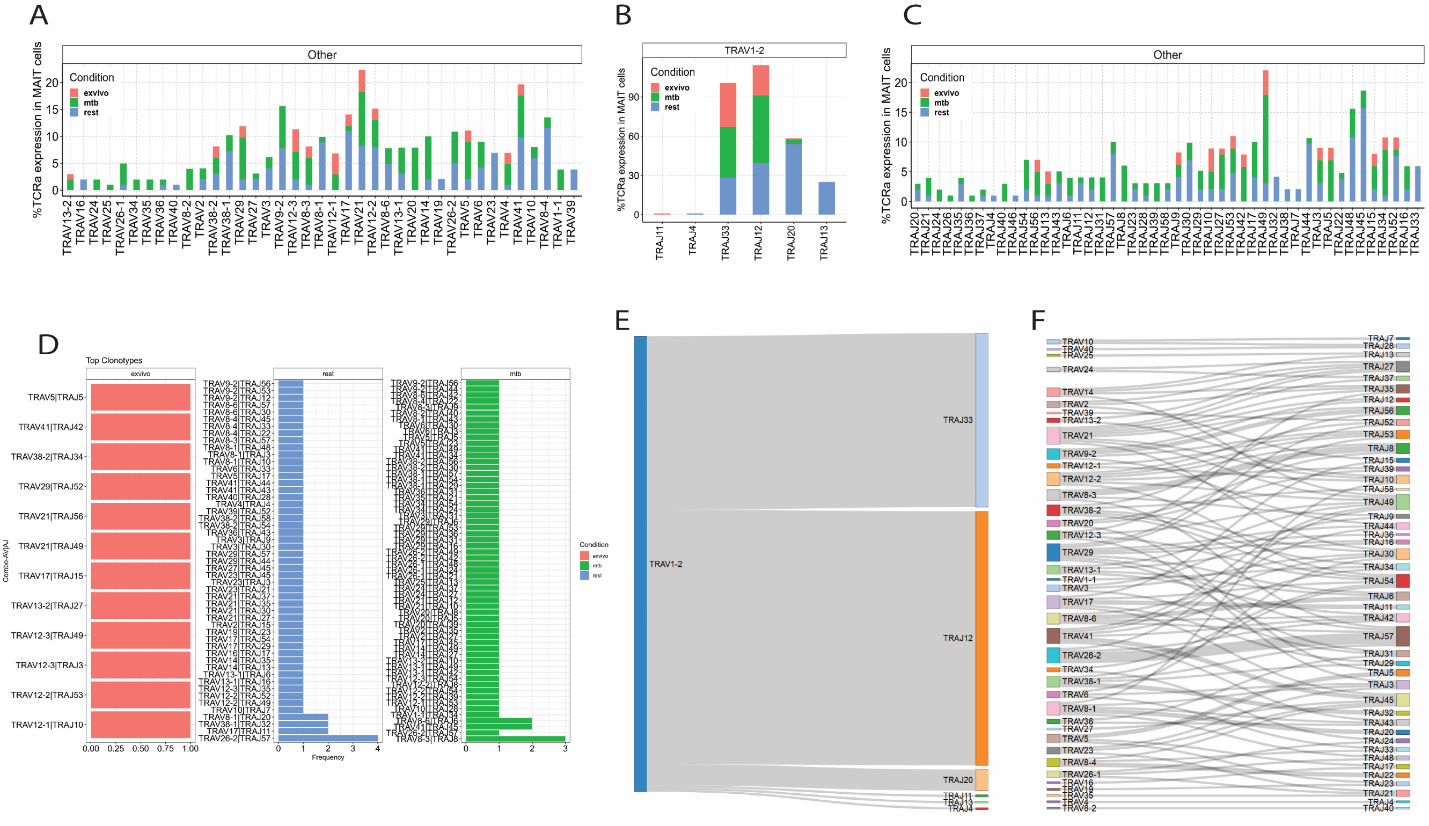


**Supplemental Figure 8: TCR𝛼 diversity in MAIT cells sequenced in *Kaur* et al.** Bar plots stratified by stimulation condition displaying **A**. Variable 𝛼 chain diversity in TRAV1-2 negative MAIT cells. **B**. J segment 𝛼 chain diversity in TRAV1-2^+^ MAIT cells and **C**. TRAV1-2 negative MAIT cells. **D.** Bar plots displaying the diversity of Variable and J-segments of 𝛼 chain in different experimental conditions. **E**. Sankey plot displaying TRAJ chain diversity in TRAV1-2^+^ and **F.** TRAV1-2 negative MAIT cells. Color codes:- **orange=ex vivo, no incubation; green=IL2+*Mtb* lysate incubation; blue=IL2 alone incubation (rest).**


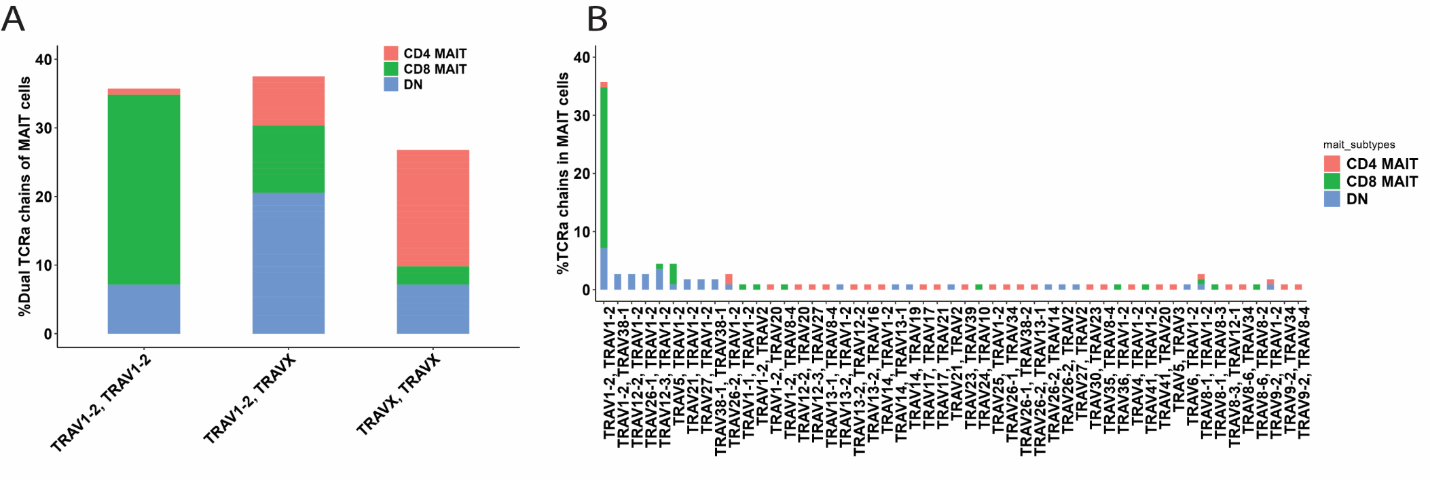


**Supplemental Figure 9: Dual TCR expression across MAIT cell subsets. A.** Bar plot displaying the frequency of α chain combinations in dual TCR-expressing MAIT cell subsets defined by TRAV1-2**^+^** or TRAVX, representing TRAV1-2 negative private α chains. Color legend applies to both panels (orange=CD4, green=CD8, blue=DN). **B.** Frequency of dual TCR combinations stratified by MAIT cell subset.


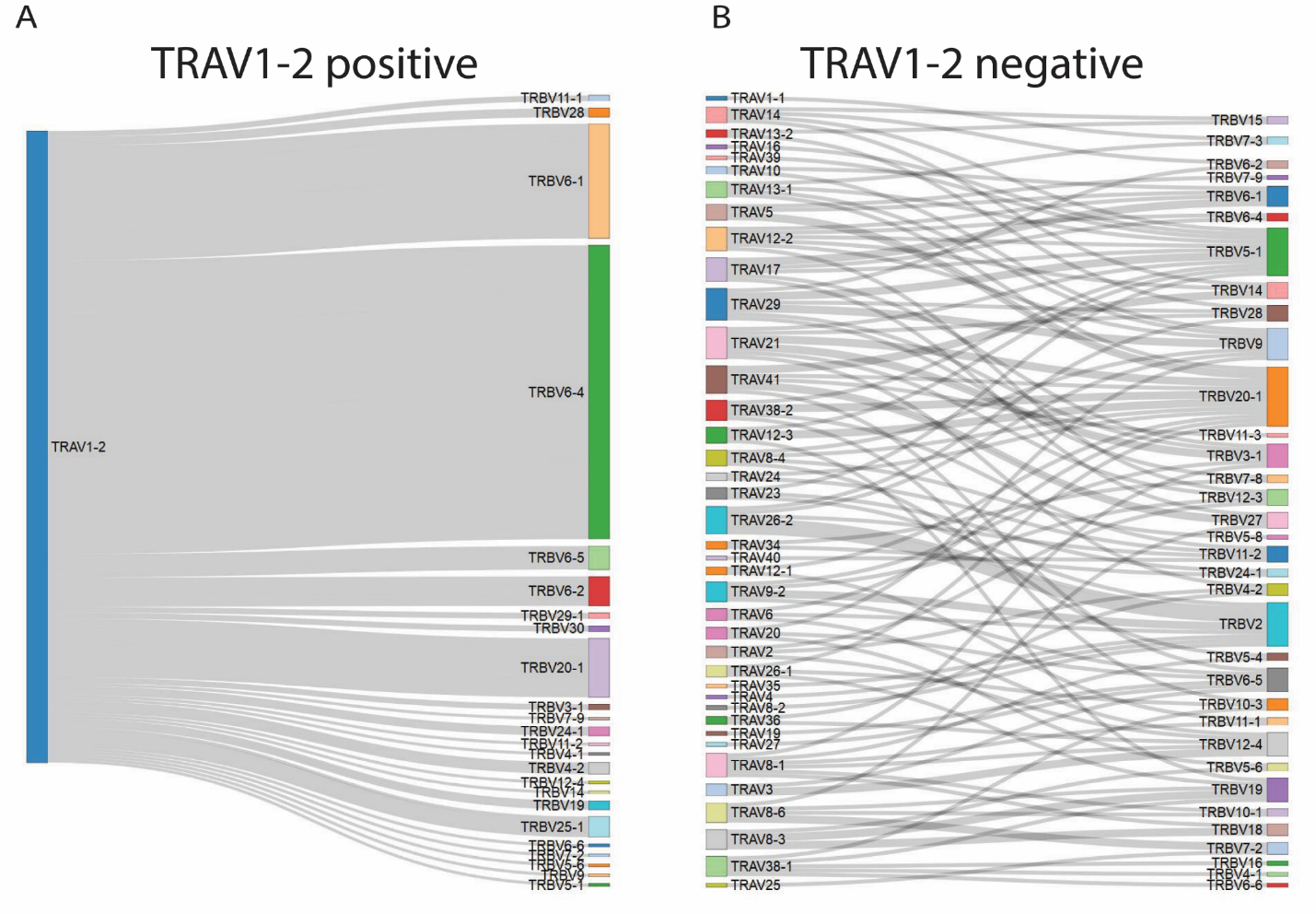


**Supplemental Figure 10: Sankey plot displaying the pairing of TRAV1-2^+/-^ MAIT cell TCRs with TRBV chains**. TRBV chain pairing with **(A)** TRAV1-2^+^ and **(B)** TRAV1-2 negative 𝛼 chains.


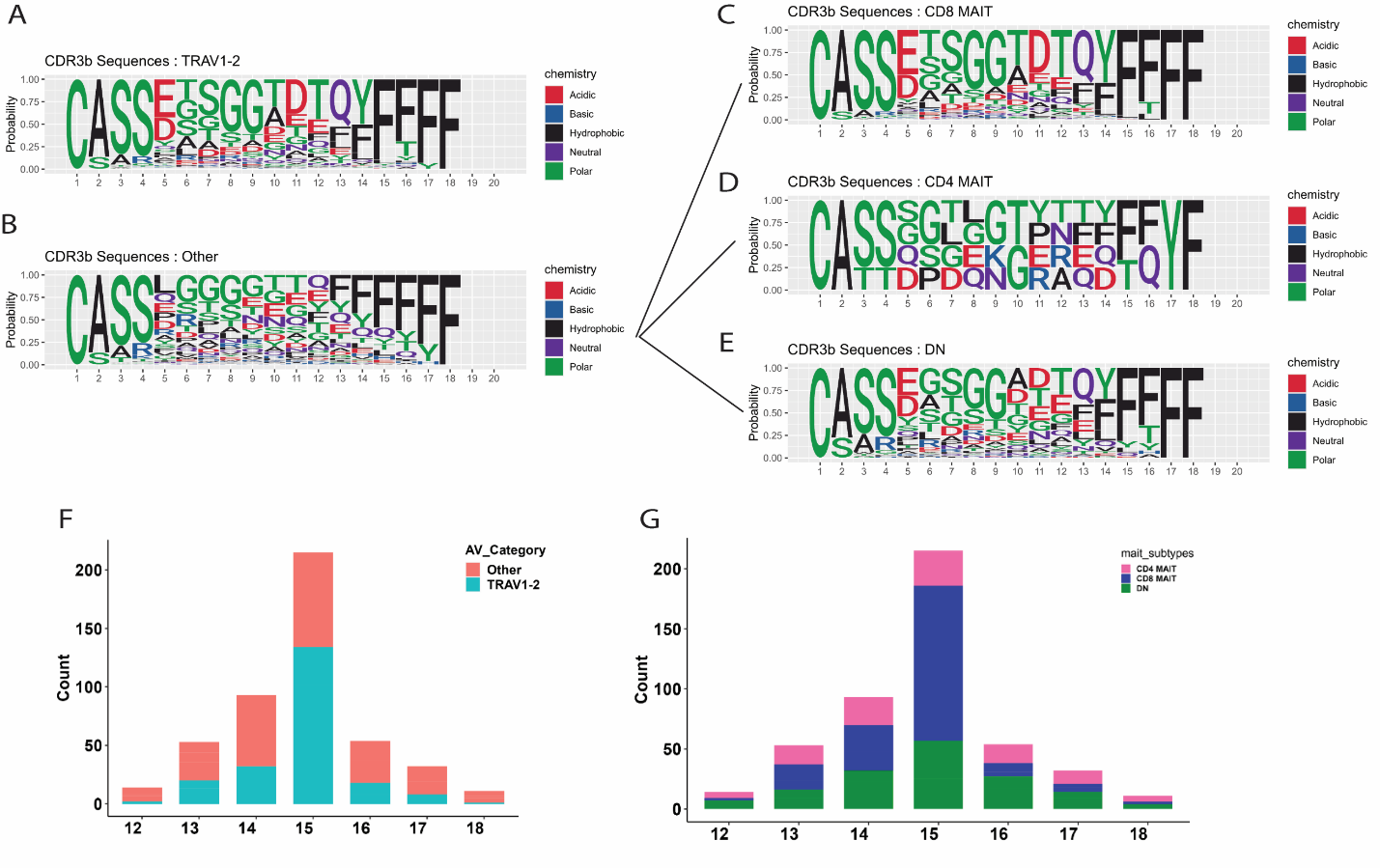


**Supplemental Figure 11: TRAV1-2^+/-^ CDR3β sequence diversity in *Kaur et al*.** Sequence logo plots displaying the CDR3β sequences expressed by **A.** TRAV1-2^+^ MAIT cells, **B.** TRAV1-2 negative MAIT cells and **C-E** MAIT cell subsets **F, G.** Bar plot displaying the amino acid length of CDR3β sequence stratified by TRAV1-2 usage **(F)** and MAIT cell subset **(G)** in *Kaur et al*.


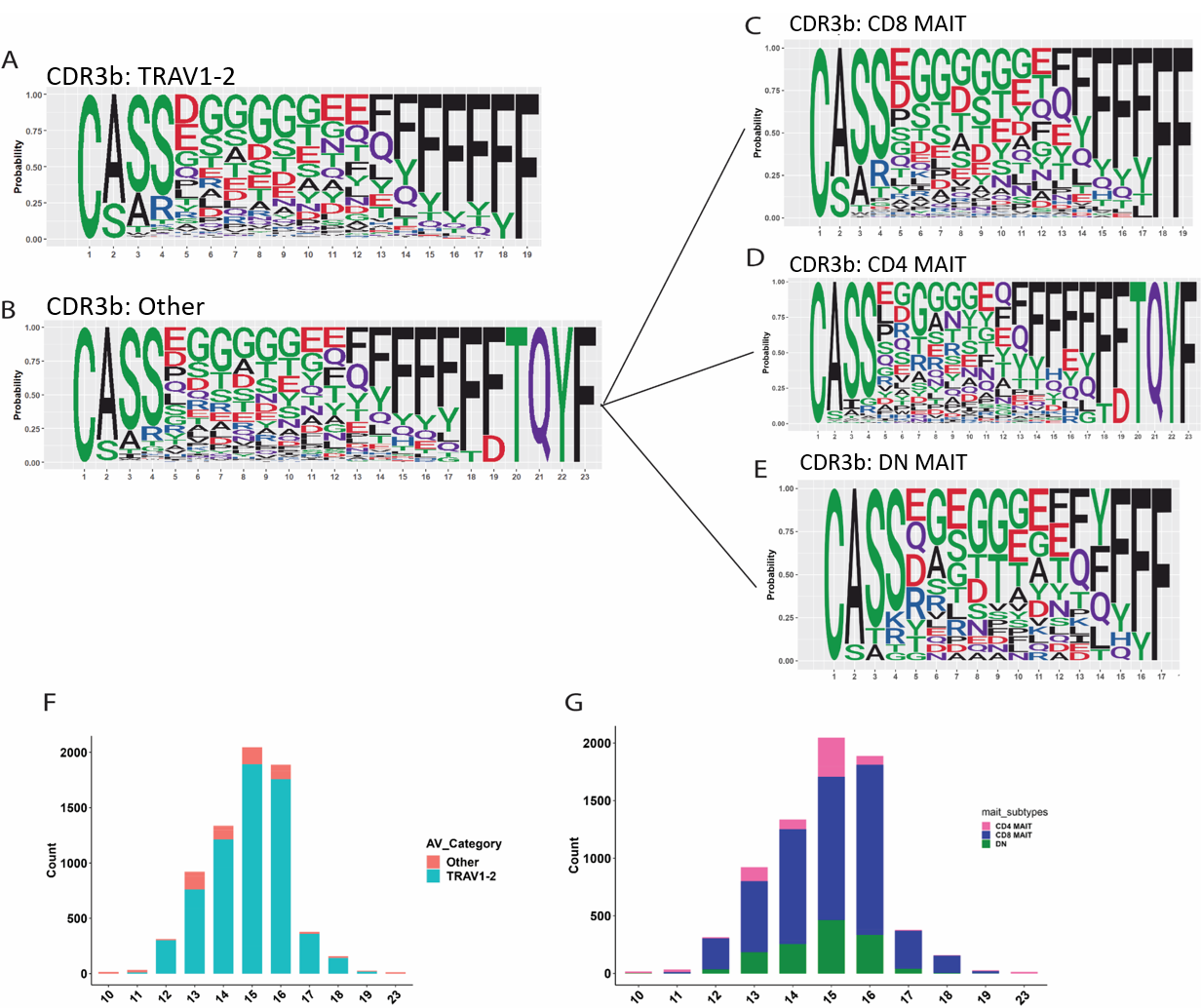


**Supplemental Figure 12: TRAV1-2^+/-^ CDR3β sequence diversity in *Garner* et al.** Sequence logo plots displaying the CDR3β sequences expressed by **A.** TRAV1-2^+^ MAIT cells, **B.** TRAV1-2 negative MAIT cells and **C-E** MAIT cell subsets in *Garner* et al. **F, G.** Bar plot displaying the amino acid length of CDR3β sequence stratified by TRAV1-2 usage **(F)** and MAIT cell subset **(G)** *in Garner* et al.
