# Supplementary material for: CD4+ mucosal-associated invariant T cells express highly diverse T cell receptors
Source: J Immunol. 2025 Nov 9;214(12):3260–72. doi: 10.1093/jimmun/vkaf260 (PMC12726071; doi:10.1093/jimmun/vkaf260)
Supplement: vkaf260_Supplementary_Data [file vkaf260_supplementary_data.zip › vkaf260_Supplementary_Data/Supplemental_Data_10-24-2025.docx]

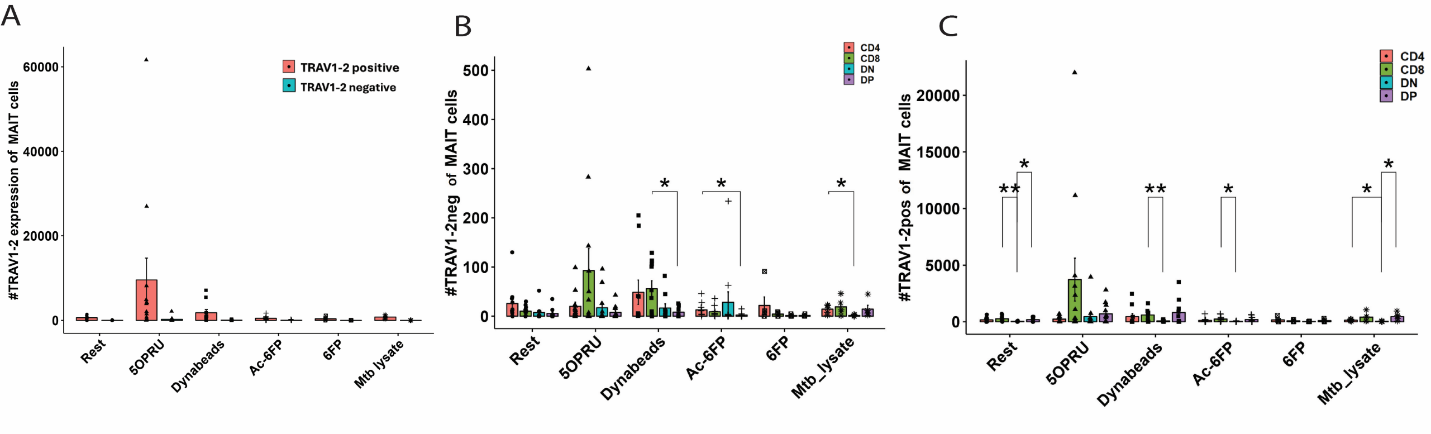


**Supplemental Figure 1: Absolute normalized counts of MAIT cells with TRAV1-2^+/-^ TCRs. A.** MAIT cell absolute normalized counts stratified by TRAV1-2 staining after 7 days of incubation in vitro with various stimuli. Normalized counts in **B.** TRAV1-2^+^ and **C**. TRAV1-2^-^ MAIT cell subsets. Absolute MAIT cell counts were normalized per 10,000 live cells. Statistical comparisons made by unpaired t-test. *p<0.05 **p<0.005


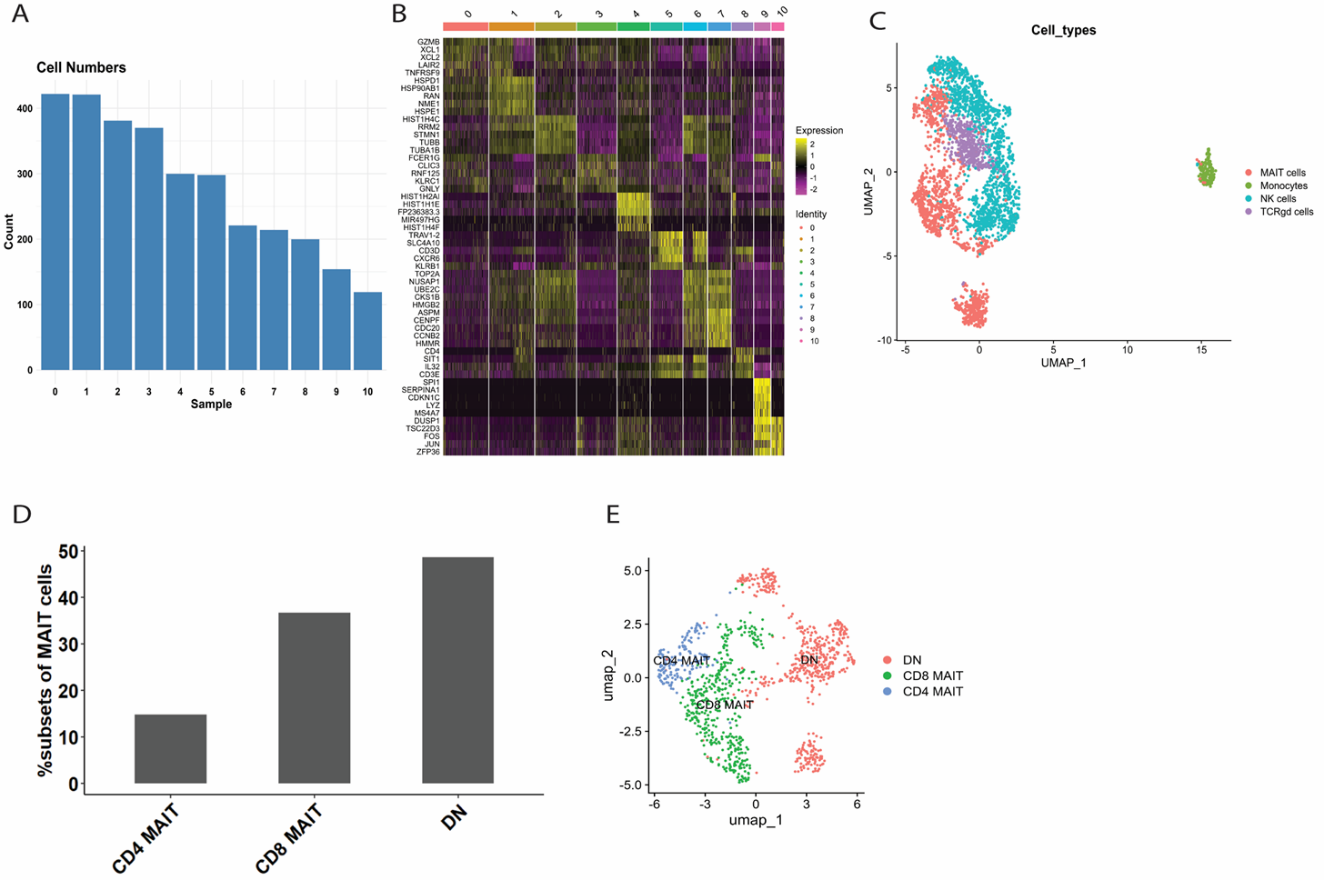


**Supplemental Figure 2: CITE-Seq analysis and visualization.** Cell count and visualization of all sequenced cells. **A**. Absolute cell count in each cluster (C0-10). **B**. Heat map displaying the top differentially expressed genes in Seurat clusters (C0-10). **C**. UMAP visualization of four cell populations found in CITE-Seq. **D**. The frequency of MAIT cell subsets in MAIT cell cluster. **E**. UMAP visualization of MAIT cell subsets.


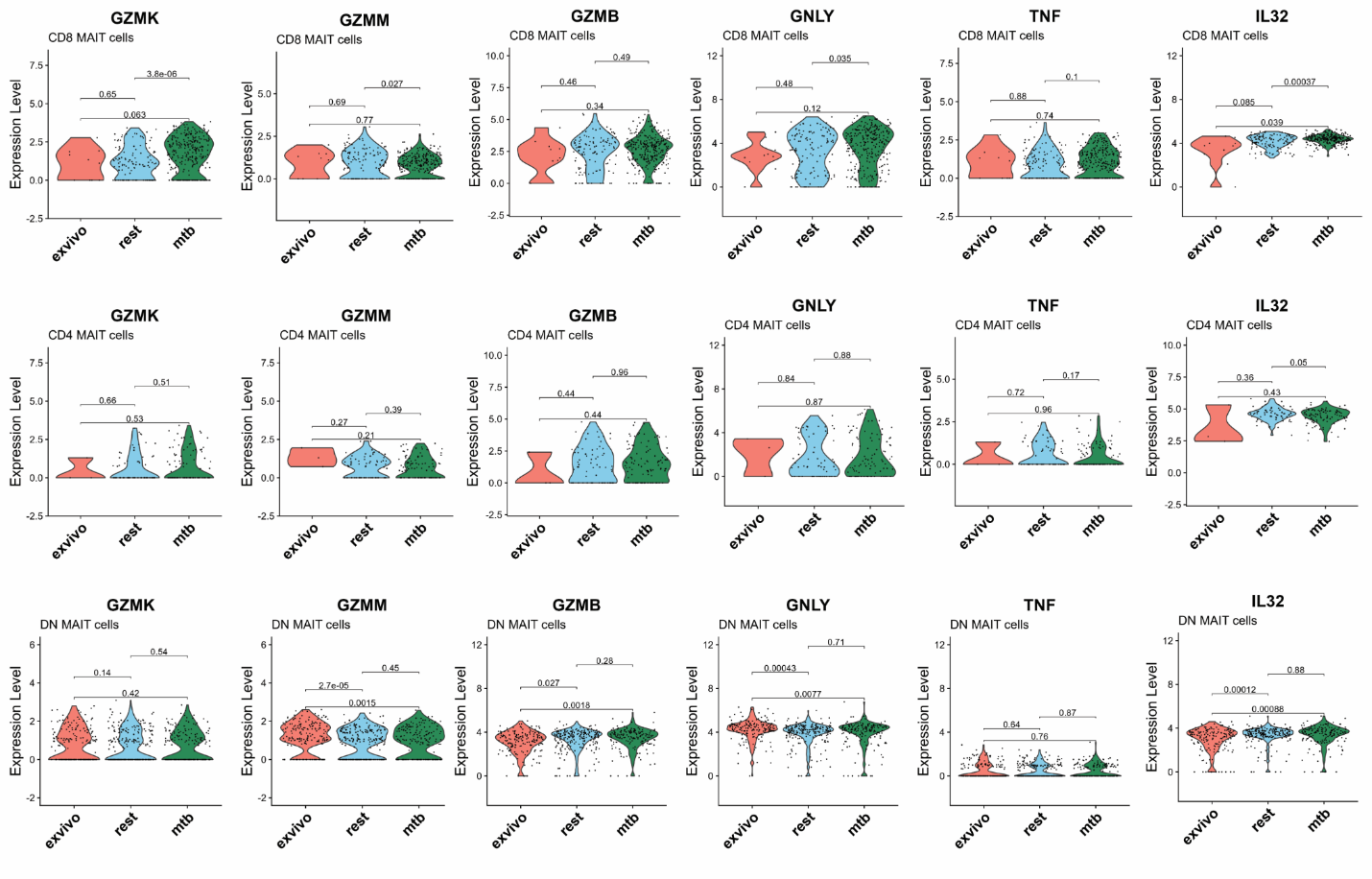


**Supplemental Figure 3: Select gene expression analysis after *Mtb* lysate induction.** Violin plots displaying the expression levels of select genes ex vivo or after coincubation for 7 days with IL2 + *Mtb* lysates relative to IL2 alone (rest). Statistical comparisons made by unpaired Wilcoxon test with reported adjusted p-values and significance level of p<0.05.


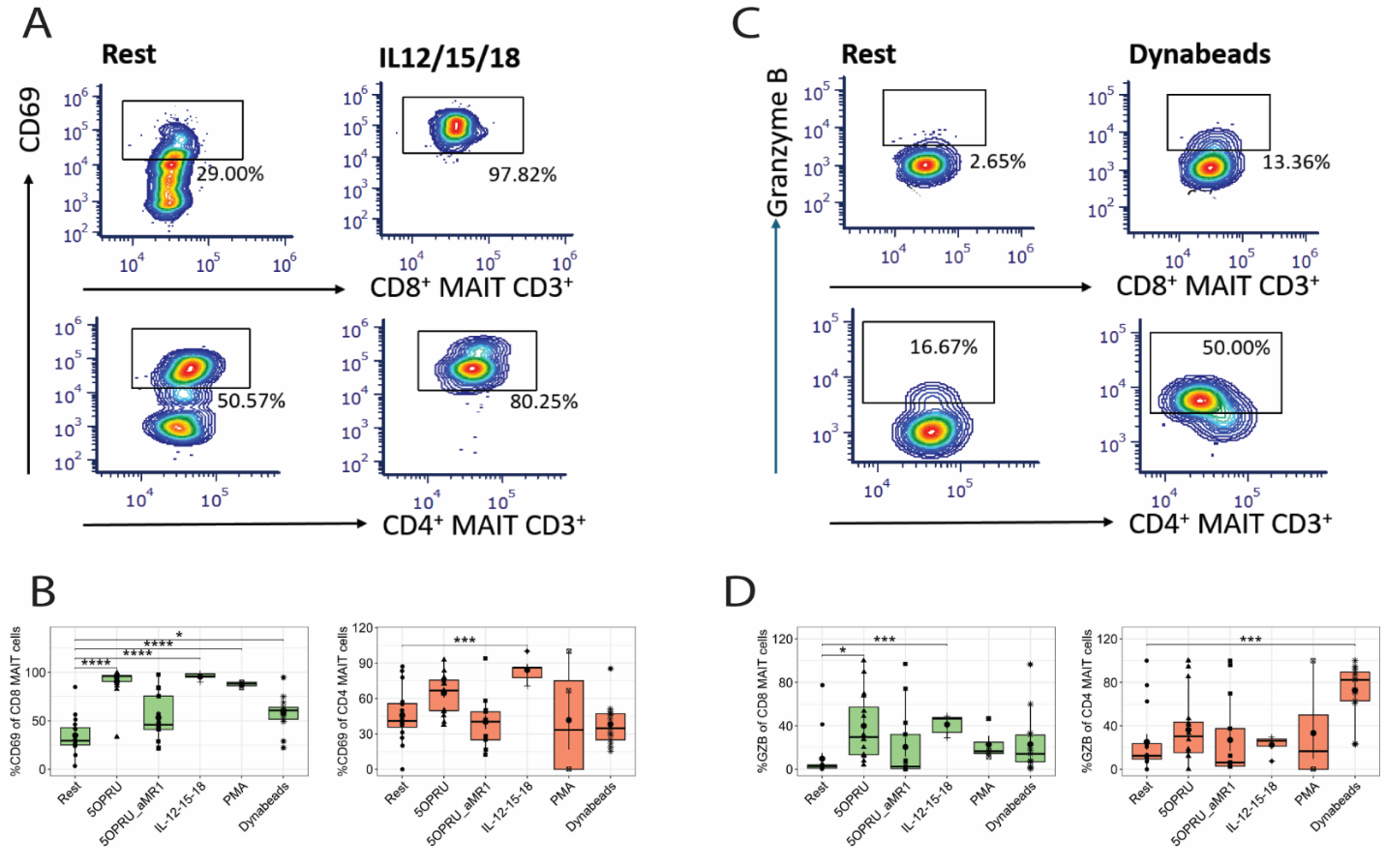


**Supplemental Figure 4: CD8^+^ and CD4^+^ MAIT cell activation and granzyme production after TCR-dependent and independent stimuli.** **A.** Representative flow contour plots demonstrating the gating strategy for CD8^+^ and CD4^+^ MAIT cells for CD69 staining with or without IL12/15/18 stimulation. **B.** Cumulative bar plots displaying the percent expression of CD69 across stimulation conditions. **C.** Representative flow contour plots demonstrating intracellular Granzyme B staining with or without anti-CD3/CD28 Dynabeads stimulation. **D.** Cumulative bar plots displaying the percent expression of Granzyme B across stimulation conditions. Statistical comparisons made by unpaired t-test. *p<0.05, ***p<0.001, ****p<0.0001


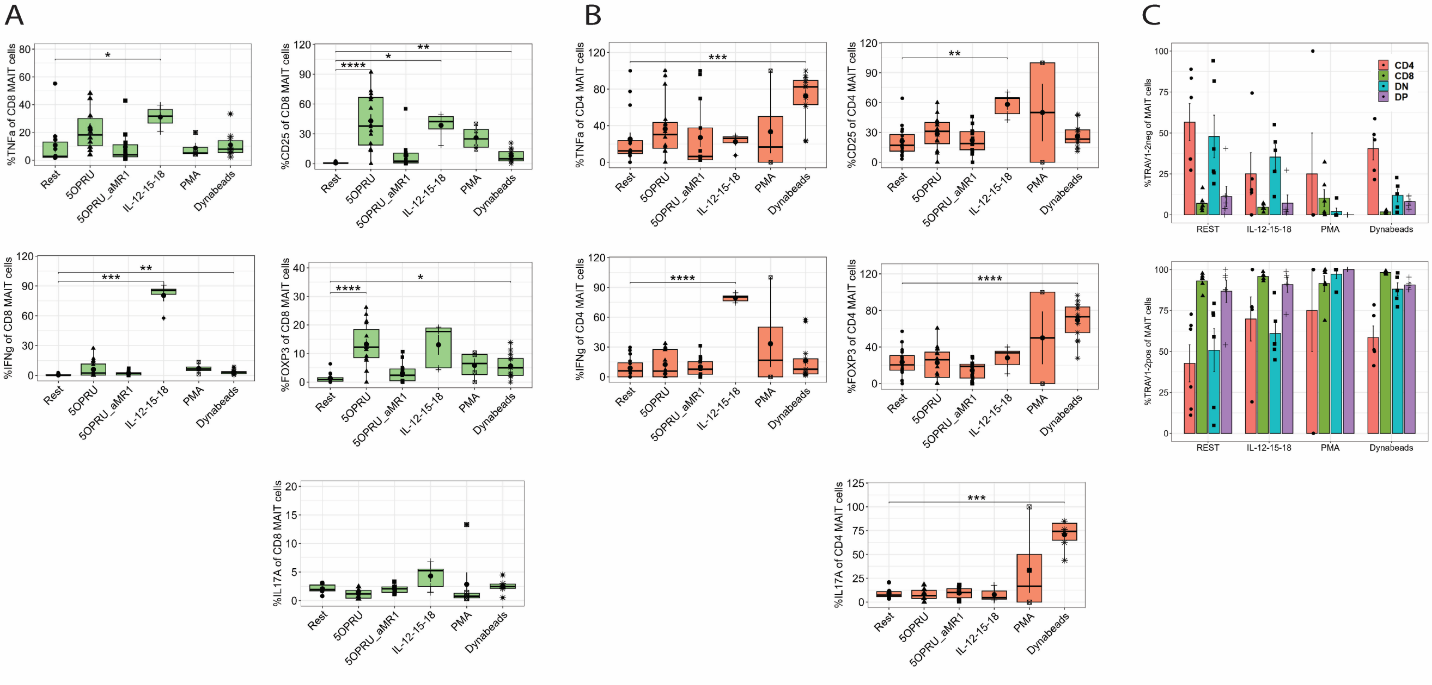


**Supplemental Figure 5: CD8^+^ and CD4^+^ MAIT cell effector function and expansion after TCR-dependent and independent stimulation.** Bar plots displaying the percent expression of CD25, IFNγ, TNFα, IL17A and FOXP3 in **A.** CD8^+^ MAIT cells and **B.** CD4^+^ MAIT cells after 16 hour incubation with various stimulation conditions. **C.** TRAV1-2^+/-^ MAIT cell expansion with various stimulation conditions for 7 days. Statistical comparisons made by Wilcoxon test. *p<0.05, **p<0.005, ***p<0.001, ****p<0.0001


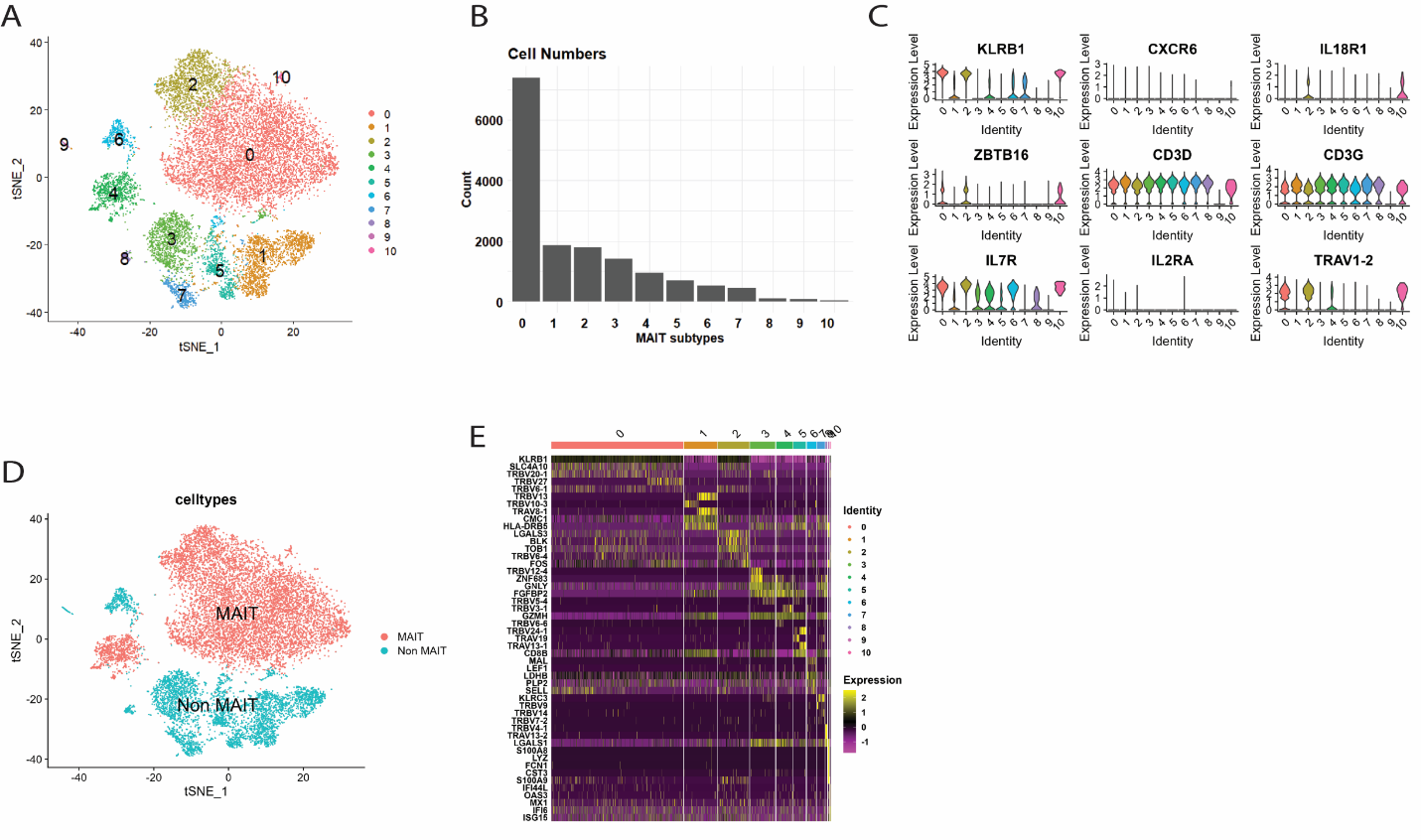


**Supplemental Figure 6:** **Visualization and gene expression profiling of cells from *Garner* et al., 2023**. **A.** t-SNE visualization of all sequenced cells in experiment 1. **B.** Absolute cell count in each cluster. **C.** Gene expression of CD3 and MAIT cell-specific genes across different clusters. **D.** t-SNE visualization of MAIT and non-MAIT cells identified in experiment 1. **E.** Heat map displaying differential gene analysis between clusters.


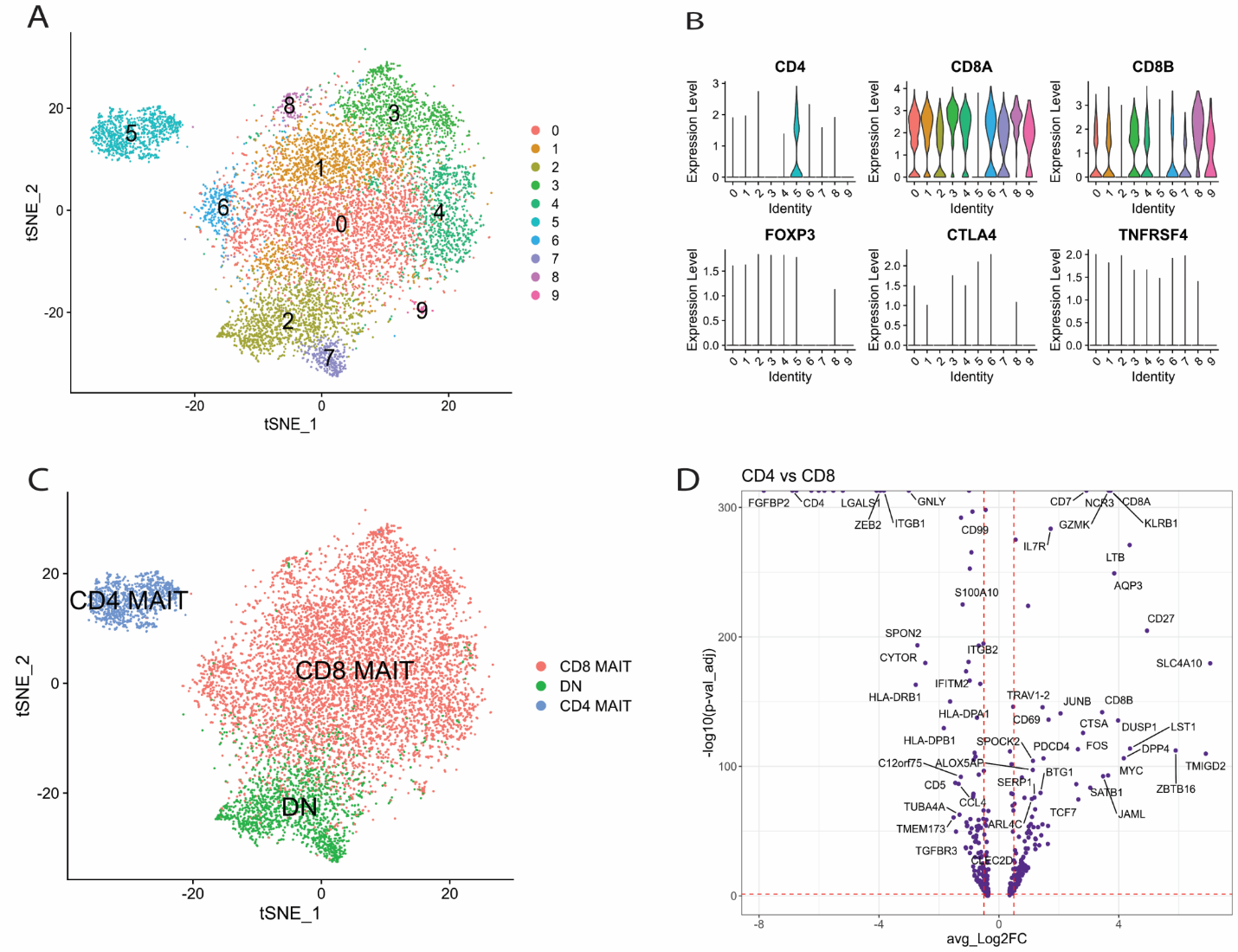


**Supplemental Figure 7: Gene expression analysis of identified MAIT cells (*Garner* et al 2023)**. **A** t-SNE visualization of MAIT cell subclusters. **B.** CD8 and CD4 coreceptor gene expression in MAIT cell subclusters. **C.** t-SNE visualization of MAIT cell subsets **D.** Volcano plot displaying differential gene expression between CD4^+^ (-log fold change) and CD8^+^ (+log fold change) MAIT cells.


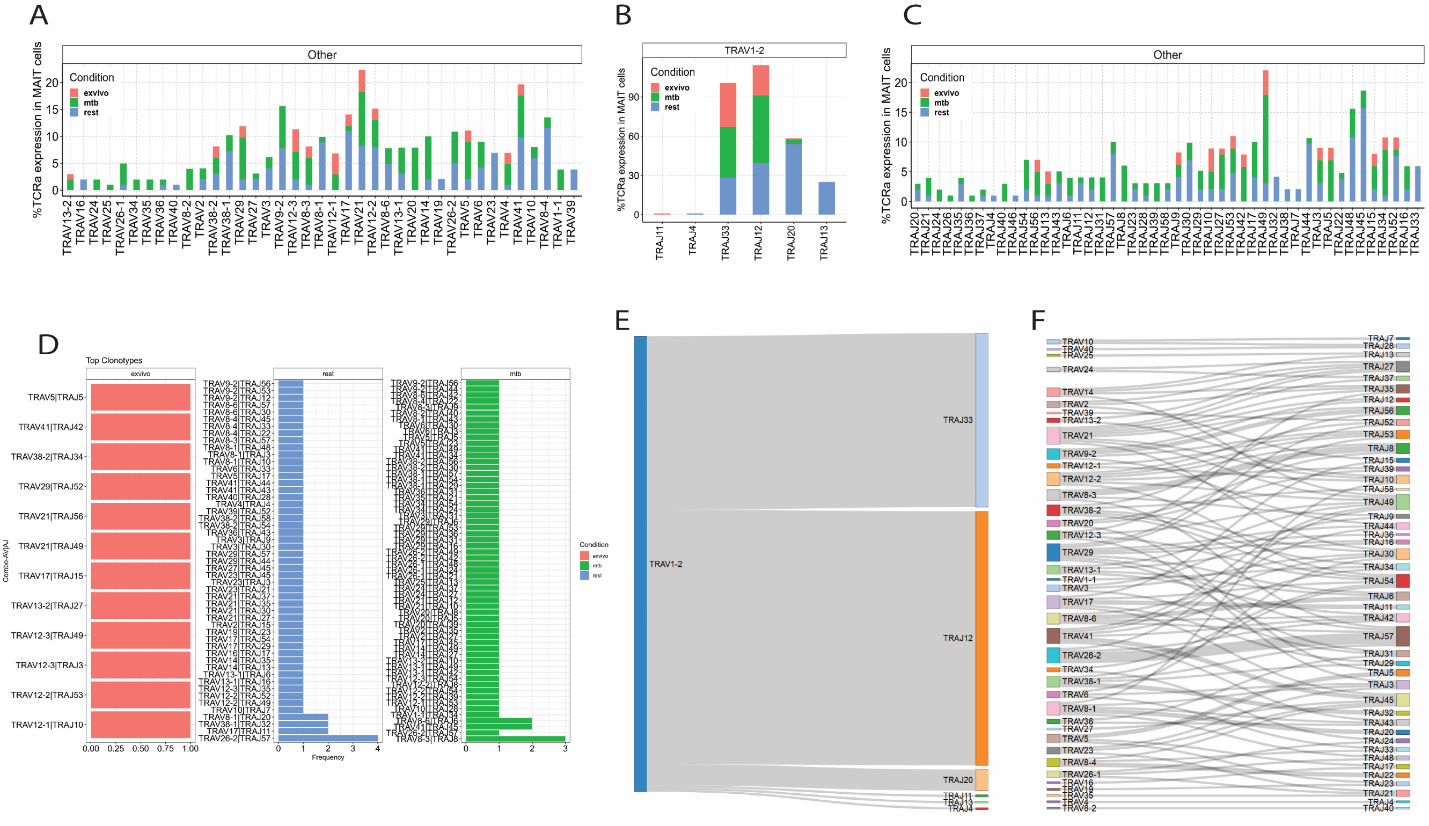


**Supplemental Figure 8: TCR𝛼 diversity in MAIT cells sequenced in *Kaur* et al.** Bar plots stratified by stimulation condition displaying **A**. Variable 𝛼 chain diversity in *TRAV1-2*^-^ MAIT cells. **B**. J segment 𝛼 chain diversity in *TRAV1-2*^+^ MAIT cells and **C**. *TRAV1-2*^-^ MAIT cells. **D.** Bar plots displaying the diversity of Variable and J segments of 𝛼 chain in different experimental conditions. **E**. Sankey plot displaying TRAJ chain diversity in *TRAV1-2*^+^ and **F.** *TRAV1-2*^-^ MAIT cells. Color codes: orange=ex vivo, no incubation; green=IL2+*Mtb* lysate incubation; blue=IL2 alone incubation (rest).


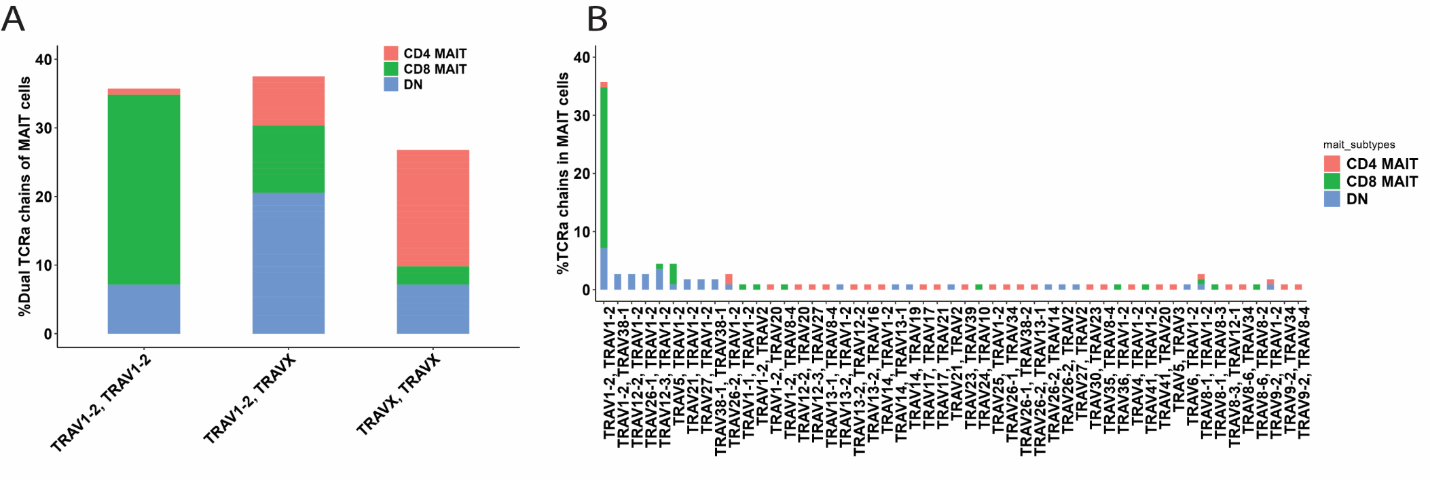


**Supplemental Figure 9: Dual TCR expression across MAIT cell subsets. A.** Bar plot displaying the frequency of α chain combinations in dual TCR-expressing MAIT cell subsets defined by *TRAV1-2***^+^** or *TRAVX*, representing *TRAV1-2*^-^ private α chains. Color legend applies to both panels (orange=CD4, green=CD8, blue=DN). **B.** Frequency of dual TCR combinations stratified by MAIT cell subset.


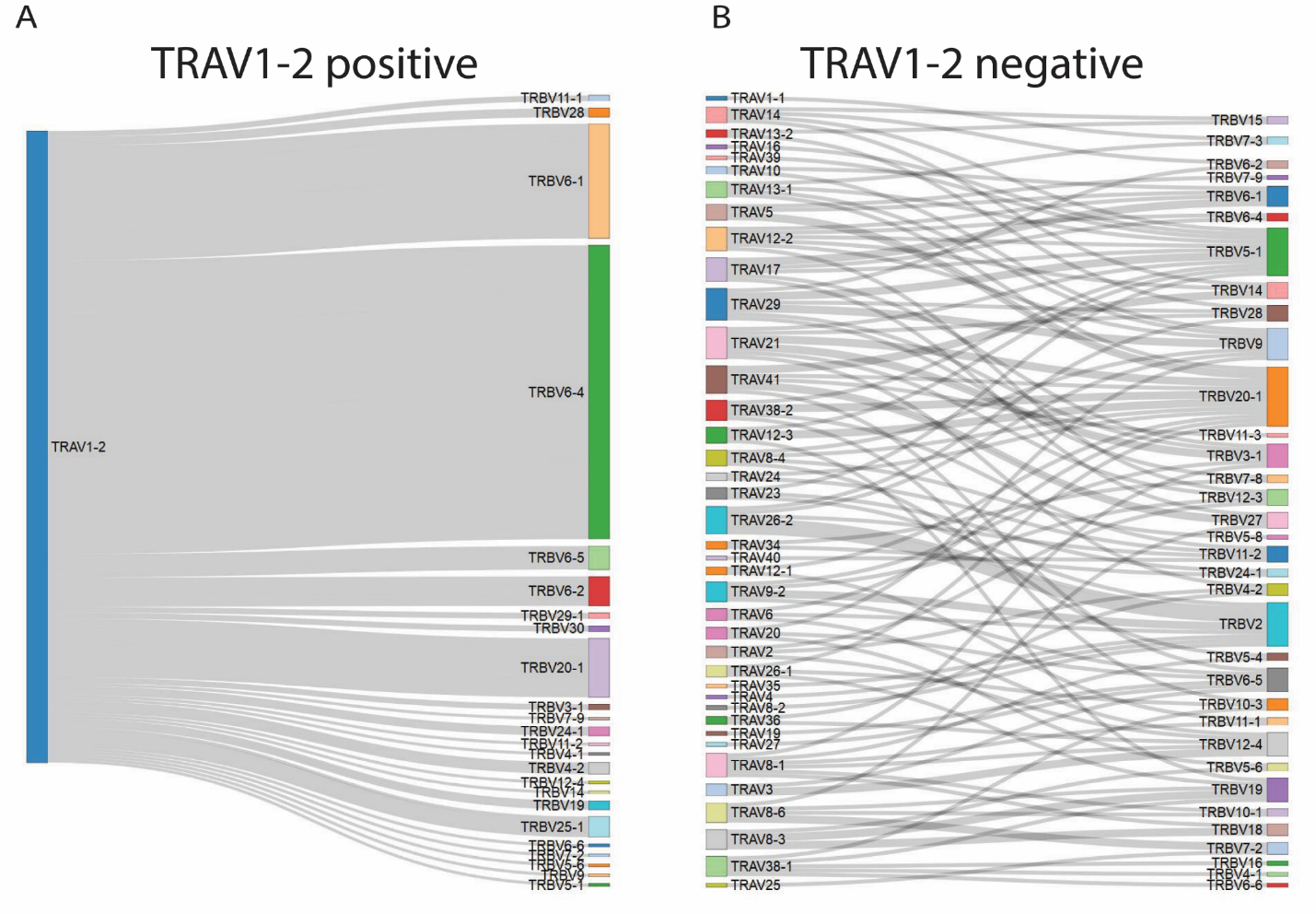


**Supplemental Figure 10: Sankey plot displaying the pairing of *TRAV1-2*^+/-^ MAIT cell TCRs with TRBV chains**. TRBV chain pairing with **(A)** *TRAV1-2*^+^ and **(B)** *TRAV1-2*^-^ 𝛼 chains.


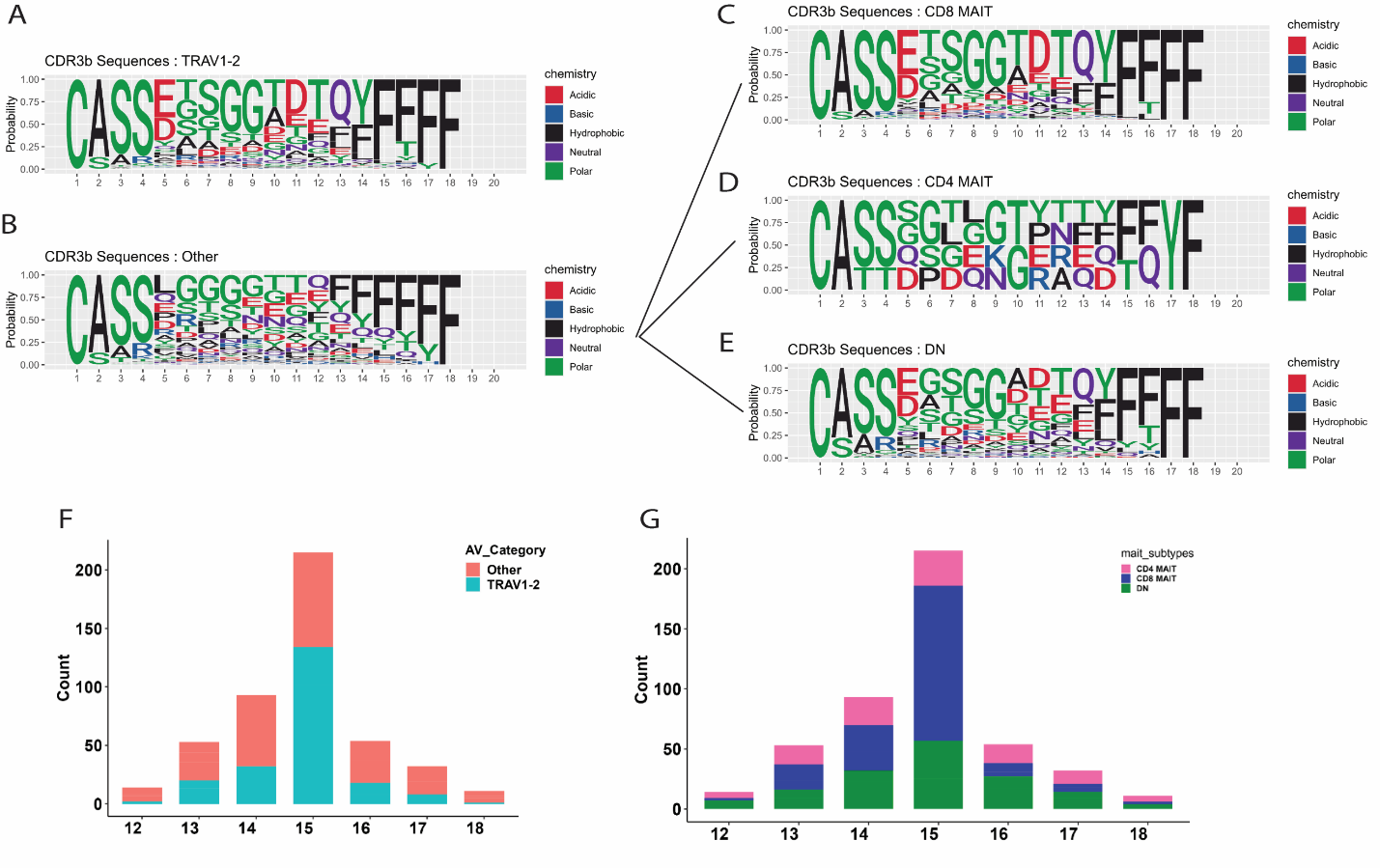


**Supplemental Figure 11: *TRAV1-2*^+/-^ CDR3β sequence diversity in *Kaur et al*.** Sequence logo plots displaying the CDR3β sequences expressed by **A.** *TRAV1-2*^+^ MAIT cells, **B.** *TRAV1-2^-^* MAIT cells and **C-E** MAIT cell subsets. **F, G.** Bar plot displaying the amino acid length of CDR3β sequence stratified by *TRAV1-2* usage **(F)** and MAIT cell subset **(G)** in Kaur et al.


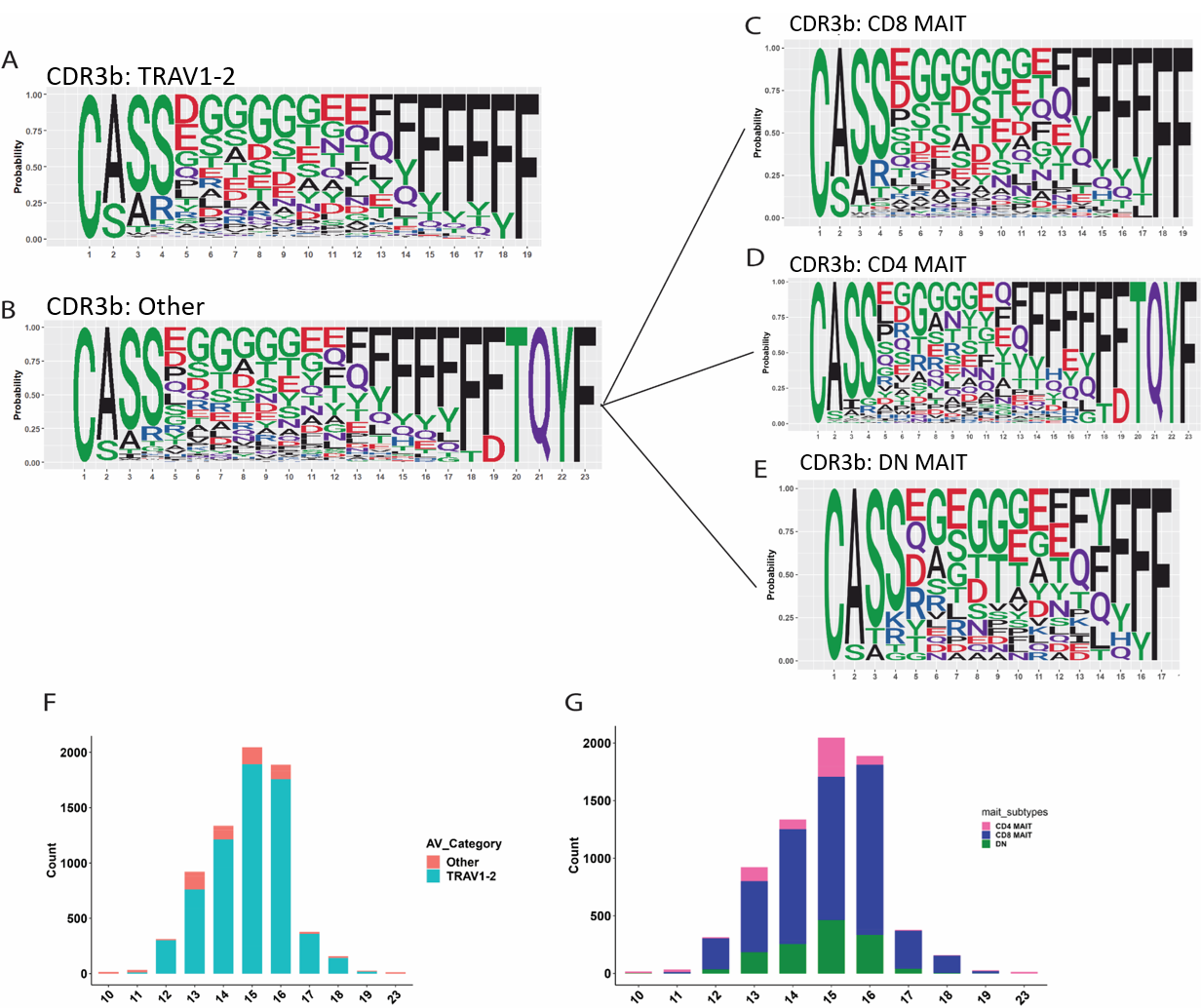


**Supplemental Figure 12: *TRAV1-2*^+/-^ CDR3β sequence diversity in *Garner* et al.** Sequence logo plots displaying the CDR3β sequences expressed by **A.** *TRAV1-2*^+^ MAIT cells, **B.** T*RAV1-2*^-^ MAIT cells and **C-E** MAIT cell subsets in *Garner* et al. **F, G.** Bar plot displaying the amino acid length of CDR3β sequence stratified by *TRAV1-2* usage **(F)** and MAIT cell subset **(G)** *in Garner* et al.
